# Why POSS-Type Compounds Should Be Considered Nanomodifiers, Not Nanofillers—A Polypropylene Blends Case Study

**DOI:** 10.3390/polym13132124

**Published:** 2021-06-28

**Authors:** Dariusz Brząkalski, Robert E. Przekop, Bogna Sztorch, Miłosz Frydrych, Daria Pakuła, Marek Jałbrzykowski, Grzegorz Markiewicz, Bogdan Marciniec

**Affiliations:** 1 Faculty of Chemistry, Adam Mickiewicz University in Poznan, 61-614 Poznan, Poland; dariusz.brzakalski@amu.edu.pl (D.B.); frydrych@amu.edu.pl (M.F.); darpak@amu.edu.pl (D.P.); 2 Centre for Advanced Technologies, Adam Mickiewicz University in Poznan, 61-614 Poznan, Poland; bogna.sztorch@amu.edu.pl; 3 Faculty of Mechanical Engineering, Bialystok University of Technology, Wiejska 45 C, 15-351 Bialystok, Poland; m.jalbrzykowski@pb.edu.pl (M.J.); g.markiewicz@doktoranci.pb.edu.pl (G.M.)

**Keywords:** nanomodifiers, additives, composites, polypropylene (PP), processing, thermal properties, low concentration, POSS, silsesquioxanes, spherosilicates

## Abstract

In this work, a series of silsesquioxanes (SSQ) and spherosilicates (SS), comprising a group of cage siloxane (CS) compounds, was tested as functional additives for preparation of isotactic polypropylene (iPP)-based nanocomposites and discussed in the aspect of their rationale of applicability as such additives. For this purpose, the compounds were prepared by condensation and olefin hydrosilylation reactions. The effect of these cage siloxane products on properties of obtained CS/iPP nanocomposites was analyzed by means of mechanical, microscopic (scanning electron microscopy-energy dispersive spectroscopy), thermal (differential scanning calorimetry, thermogravimetry), thermomechanical (Vicat softening point) analyses. The results were compared with the previous findings on CS/polyolefin composites. The role of CS compounds was discussed in terms of plastic processing additives.

## 1. Introduction

In the study of polymer systems with practical application, the cognitive value of the conducted research should be seen on par with the rationality of their use in the light of the final effect. Compounds of the CS type, commonly known under their trade name POSS^®^ (Polyhedral Oligomeric Silsesquioxanes, trademark registered by Hybrid Plastics Inc.), due to the specificity of their structure and methods of obtaining, are not the cheapest modifiers. Their market price ranges from 200 to 3000 USD per 1 kg [1]. Their price is also strongly correlated with the price of simple organosilicon compounds—organofunctional silanes commonly used as adhesion promoters in polymer composite systems [2]. Bearing in mind the above, we would like to discuss the areas in which these compounds can really be modifiers compared to known and existing solutions. Simple and cheap materials such as polyolefins (polyethylene, polypropylene) are very well-studied systems for which changes in mechanical or thermal parameters, and then in processing properties, may be caused by many fillers or modifiers [3,4].

So why try to use POSS compounds as a modifier of the properties of such materials? The study attempts to find convincing thermal and mechanical effects of polypropylene modified with a number of structurally diverse POSS compounds in the low concentration range (0.1–1% *w*/*w*). A very important problem in the use of POSS compounds is their solubility/dispersal under processing conditions (polymer melt); many of the POSS compounds do not show a sharp melting point, and their basic phase transformation is sublimation at temperatures above 250 °C [5,6]. Such POSS compounds under processing conditions will have considerable difficulty in dispersing in the polymer matrix due to their crystalline form and low solubility/non-fusibility. Therefore, their use will be limited by the concentration limit, above which the role of the nanomodifier will be reduced due to formation of polycrystalline agglomerates, similarly to inorganic nanofillers. An important critical statement was made by Herbert et al. that many studies including silsesquioxane-based composites were concerned around high concentrations of the organosilicon additives used (even exceeding 10% *w*/*w*), while these compounds should be tested at loadings within the limits of their effective dispersion and compatibility with the polymer [7]. From a different point of view, more and more often, the term ‘nanofillers’ is used in the literature in regard to POSS compounds. According to the definition, fillers are added to plastics to reduce their cost per unit volume and/or to improve such mechanical properties as hardness or Young’s/flexural modulus of a given material. Further speaking, a filler may be ‘a relatively non-adhesive substance added to an adhesive to improve its working properties, permanence, strength, and other qualities; any compounding ingredient, usually in dry, powder form, added to rubber in substantial amount to improve quality of lower cost’ [8]. According to this definition, fillers include china clay, wood flour, silica, silicates, carbon black, fibrous materials, or aluminum powder that markedly enhance the performance of a polymer, and their cost is reasonably low, either lower than that of the neat polymer or close to it. It should be noted that there is a clear difference between additives that cause changes in the material (fillers) and additives that affect the processing properties of the material (modifiers). On the basis of the conducted research, we suggest that in the case of polyolefin systems and other polymer systems in which no unequivocal effects such as permanence, strength, or cost reduction occurred, the term nanofiller in relation to compounds of the POSS type should not be used.

In recent years, there has been a growing interest in the use of polyhedral oligomeric silsesquioxanes (SSQ) or mono- or octa-functional spherosilicates (SS) as modifiers of properties of various classes of materials, especially of organic polymer origin. Silsesquioxanes and their derivatives, which are hybrid compounds of inorganic–organic structure (inorganic core and organic functional groups connected with silicon atoms at the corners of the core) [9] have been introduced into the polymer matrix by several means. The reported methods include reactive processes (copolymerization reaction, chemical grafting, reactive extrusion, irradiation) or traditional processing methods common for thermoplastics, such as injection molding, extrusion, and calendaring [10,11,12]. Such modification enables the improvement of the physicochemical, rheological, and/or mechanical properties of the resulting (nano)composite, allowing for targeting of a given product to a specific area of (potential) application. The most important feature of silsesquioxanes and spherosilicates is the wide possibility of their functionalization by introducing functional side groups responsible for giving the materials specific properties and allowing the modifier to chemically interact with the polymer matrix, as well as tailoring the physicochemical character of a cage siloxane itself. The use of silsesquioxane or spherosilicate derivatives as modifiers of composite materials can significantly change the properties of the material, e.g., increased corrosion resistance [13], improvement of mechanical properties [14], crystallization behavior [15], surface properties (hydrophilic–hydrophobic character) [16,17], thermal stability [18], flame resistance [19,20,21], or processability, mainly melt rheology [22]. They may also be used as antioxidants [23] or nanoparticle dispersants for plastics [24,25]. Cage siloxanes have been studied as functional and processing additives for polyolefins, e.g., polyethylene or polypropylene [26], as well as other thermoplastics, including PES [27], PPS [28], PEO [29], or different grades of polyamide (PA) [30]. The effect of these additives on the properties of the base polymer is highly dependent on the level of dispersion of the additive within the polymer matrix and the CS–matrix interactions (either macroscopic or molecular level ones). For this reason, it should be distinguished if the introduction of a given additive to the base polymer results in formation of a composite or a nanocomposite. In our earlier works, we presented the influence of silsesquioxane- and spherosilicate-based additives on the properties of polyethylene- [31,32] and PLA-based [33] composites. For polypropylene, silsesquioxane-doped composites thereof were studied towards their crystallization behavior, mechanical properties, processing rheology, and thermal stability [34]. Fu presented the crystallization behavior of silsesquioxane-doped PP under different conditions, including shear-induced process [35]. Fina et al. described the influence of octamethyl-, octaisobutyl- and octaisooctyl-SSQs on the thermal and morphological characteristics of the prepared composites [36]. Kamyab et al. applied glycidoxypropylhepta(*iso*butyl)silsesquioxane as a compatibilizer for PCL/PP blends characterized by shape memory properties [37]. Zaharescu et al. reported improved gamma radiation resistance of PP modified with a series of functionalized hepta(*iso*butyl)silsesquioxanes [38]. Zhang et al. presented a synergistic effect of octamethylsilsesquioxane as a support for NA-40 nucleating agent [39]. Polypropylene, due to its satisfactory mechanical strength, moderate hardness and acceptable impact resistance, hydrophobic properties, as well as very good chemical resistance against several agents (including salt solutions, strong non-oxidizing acids, bases, alcohols, fats, oils, esters, ketones) and low production costs, is one of the thermoplastics of high industrial importance [40]. A particularly important feature from the point of view of designing new or improved materials is the ease of processability, either in the injection molding process, or different variants of extrusion process, including extrusion blow molding or blown film/fiber production. Currently, PP, due to its properties, is used as an alternative to materials based on metal, glass, or natural materials such as wood, which allows it to be used in many industries, including transport, construction, electronics, medicine, and the packaging market [41,42,43,44,45,46]. Despite good physicochemical properties, when compared to numerous other plastics, it has lower Young’s modulus, hardness, or softening temperature, which introduces significant limitations in the applications for this material [47]. Therefore, the process of PP modification arouses more and more interest in both the scientific and industrial areas [48].

In this work, the effect of cage siloxanes with different functional groups (including vinyl, alkyl, chloroalkyl, oxirane) as functional additives to iPP is described, including the compatibility of modifiers with the polymer matrix. In order to obtain homogeneous batches, the modifiers were incorporated into the polymer matrix by a melt blending process. The obtained (nano)composites were tested in terms of thermal stability; mechanical properties, rheological properties, and phase transformations (melting and crystallization points) were also determined.

## 2. Materials and Methods

### 2.1. Materials

Isotactic polypropylene (iPP), Moplen HP456J grade, was purchased from BasellOrlen Polyolefins (Poland). The chemicals were purchased from the following sources: Tetraethoxysilane (TEOS), chlorodimethylsilane, chlorodimethylvinylsilane, isobutyltrimethoxysilane, tetramethylammonium hydroxide (TMAH) 25% methanol solution from ABCR, (R)-(+)-limonene, allyl-glycidyl ether, toluene, chloroform-d, Karstedt’s catalyst xylene solution from Aldrich, P_2_O_5_ from Avantor Performance Materials Poland S.A. (Gliwice, Poland). Toluene was degassed and dried by distilling it from P_2_O_5_ under argon atmosphere. Silsesquioxane and spherosilicate compounds were prepared according to literature reports provided in Table 1.

### 2.2. Analyses

^1^H, ^13^C, and ^29^Si nuclear magnetic resonance (NMR) spectra were recorded at 25 °C on a Bruker Ascend 400 and Ultra Shield 300 spectrometers using CDCl_3_ as a solvent. Chemical shifts are reported in ppm with reference to the residual solvent (CHCl_3_) peak for ^1^H and ^13^C. Fourier transform-infrared (FT-IR) spectra were recorded on a Nicolet iS50 Fourier transform spectrophotometer (Thermo Fisher Scientific, Waltham, MA, USA) equipped with a diamond ATR unit with a resolution of 0.09 cm^−1^. Thermogravimetry (TG) was performed using a NETZSCH 209 F1 Libra gravimetric analyzer. Samples of 5 ± 0.2 mg were placed in Al_2_O_3_ crucibles. Measurements were conducted under air atmosphere (flow of 20 mL/min) in the range of 30 ÷ 800 °C and a 10 °C/min heating rate. Differential scanning calorimetry (DSC) was performed using a NETZSCH 204 F1 Phoenix calorimeter. Samples of 6 ± 0.2 mg were placed in an aluminum crucible with a punctured lid. The measurements were performed under nitrogen in the temperature range of −30 ÷ 200 °C and at a 5 °C/min heating rate, and T_m_ was measured from the second heating cycle. SEM/EDS analyses were recorded on a Quanta FEG 250 (FEI) instrument; SEM at 5 kV and EDS at 30 kV, respectively. The samples were frozen in liquid nitrogen and fractured with pliers to reveal a surface satisfactory for an analysis. The samples were taken from the extrudate obtained during preparation of the desired final concentration composites (see Section 2.3). For flexural and tensile strength tests, the obtained materials were printed into type 1B dumbbell specimens in accordance with EN ISO 527:2012 and EN ISO 178:2006. Tests of the obtained specimens were performed on a universal testing machine INSTRON 5969 with a maximum load force of 50 kN. The traverse speed was set at 50 mm/min for tensile strength measurements, at 1 mm/min for the determination of Young’s modulus, and at 1 mm/min for flexural strength. For all the series, six measurements were performed. For tribological tests, samples in the shape of mandrels of φ6 × 20 mm dimensions were used. A dial made of 316LV steel was used as a counter-sample. The tests were performed using a pin on a disc tribological tester. The tests were carried out with a unit pressure p = 2 MPa, sliding speed v = 0.25 m/s, and a friction time t = 30 min. The signal was recorded with a Hottinger bridge (Hottinger Baldwin Messtechnik) and processed in QuantumX + CatmanEasy software. The obtained data were processed using the Statistica 13 PL program. Each measurement was repeated three times. Each test was performed on a fresh disc surface (by changing the friction radius) with its initial roughness Ra = 0.3 µm. Due to the variable friction radius, the rotational speed of the disc was controlled so as to obtain the same linear speed v and the same friction path L in each test cycle. Vicat measurements were performed in accordance with ISO 306, B50 method (50 N load, 50 °C/h heating rate). Tests were performed on an Instron CEAST HV3 Vicat tester.

### 2.3. Preparation of (Nano)Composites

In a typical procedure, about 200 g of iPP was rolled on a two-roll mill until complete melt, after which the chosen modifier was added in a quantity corresponding to 5% of the final masterbatch content, and the composition was rolled together at 190 °C until it became completely homogeneous or until no more improvement of homogeneity was observed. After that, the composition was taken off the rolls and set to cool down. It was ground in the low-speed mill and the obtained masterbatch granulate was then diluted to 1% by mixing it with the granulate of neat iPP and extruding it on a single-screw extruder at 30 RPM, the extrudate being simultaneously granulated. Temperature zones for extrusion were as follows (from feed to die): 80 °C, 180 °C, 190 °C, 170 °C. Subsequently, 0.1%, 0.25%, and 0.5% concentration composites were obtained by diluting 1% granulate with neat iPP in a proper proportion in a similar fashion. The obtained granulates were then measured by TG, DSC, and SEM-EDS techniques and processed into standard dumbbell specimens by injection molding. For tribological tests, samples were injection molded as mandrels with dimensions of φ6 × 20 mm. For injection molding, the following parameters were applied: temperature zones of the plastifying unit (from feed to die): 190 °C, 200 °C, 190 °C, 180 °C, injection pressure 50 bar, holding pressure 55 bar, holding time 10 s, cooling time 18 s. The parameters of the injection process were developed based on the visual quality of the molded parts and were kept the same for all injection tests.

## 3. Results and Discussion

### 3.1. Characterization of the Obtained Modifiers

**Figure 1:**
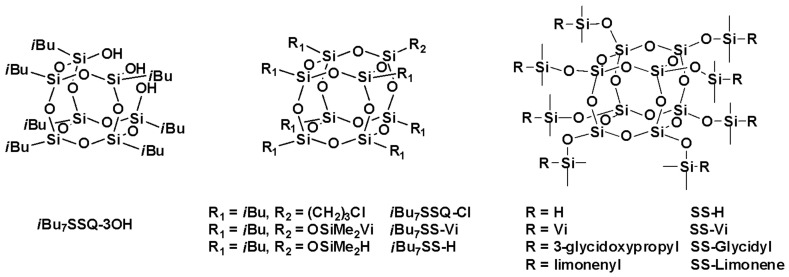
Structures of the cage siloxane compounds studied in this work.

In Table 1, a series of silsesquioxane and spherosilicate compounds obtained according to the literature procedures and used to prepare iPP-based nanocomposites are collected. SS-H was applied only to prepare SS-Glycidyl and SS-Limonene. The synthesis of the used modifiers was reported elsewhere, which is given in the Table 1. Figure 1 presents their structures together with the compound codes used throughout the whole manuscript.

The silsesquioxane and spherosilicate compounds were investigated by ^1^H, ^13^C, and ^29^Si NMR and FT-IR spectroscopy to prove their purity and structure and completion of hydrosilylation reactions (~99% for all examples, see Supplementary Information).

### 3.2. SEM and EDS Imaging

**Figure 2:**
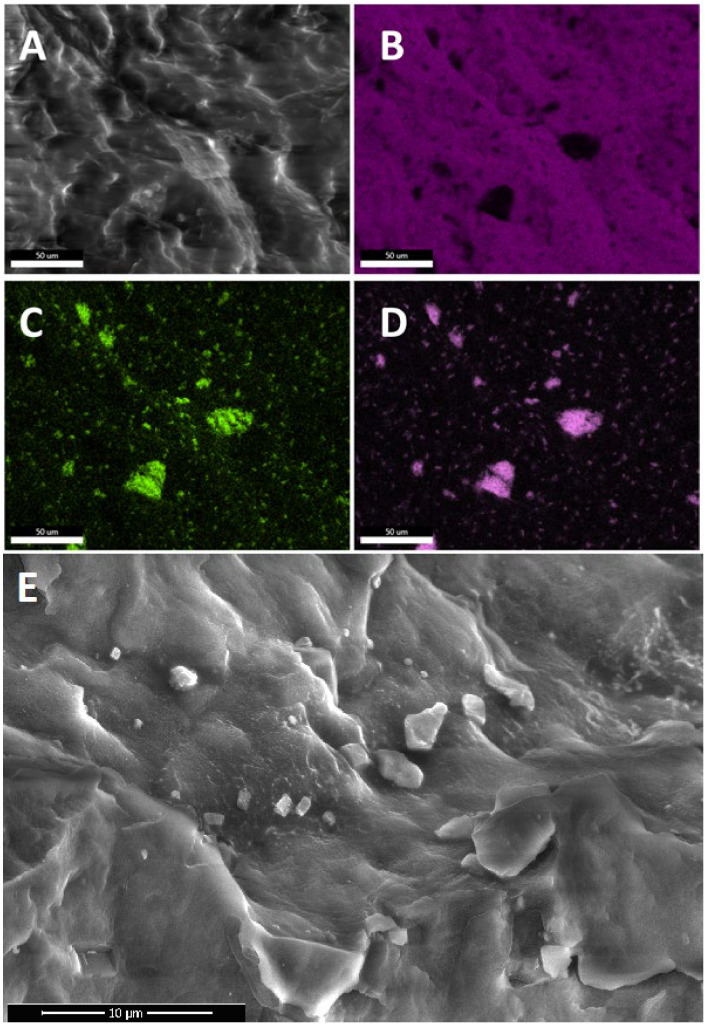
SEM (**A**,**E**) and EDS (**B**–**D**) images of SS-Vi/iPP 5% masterbatch. (**A**)—field of view SEM, (**B**)—carbon EDS, (**C**)—oxygen EDS, (**D**)—Silicon EDS, (**E**)—high resolution SEM.

**Figure 3:**
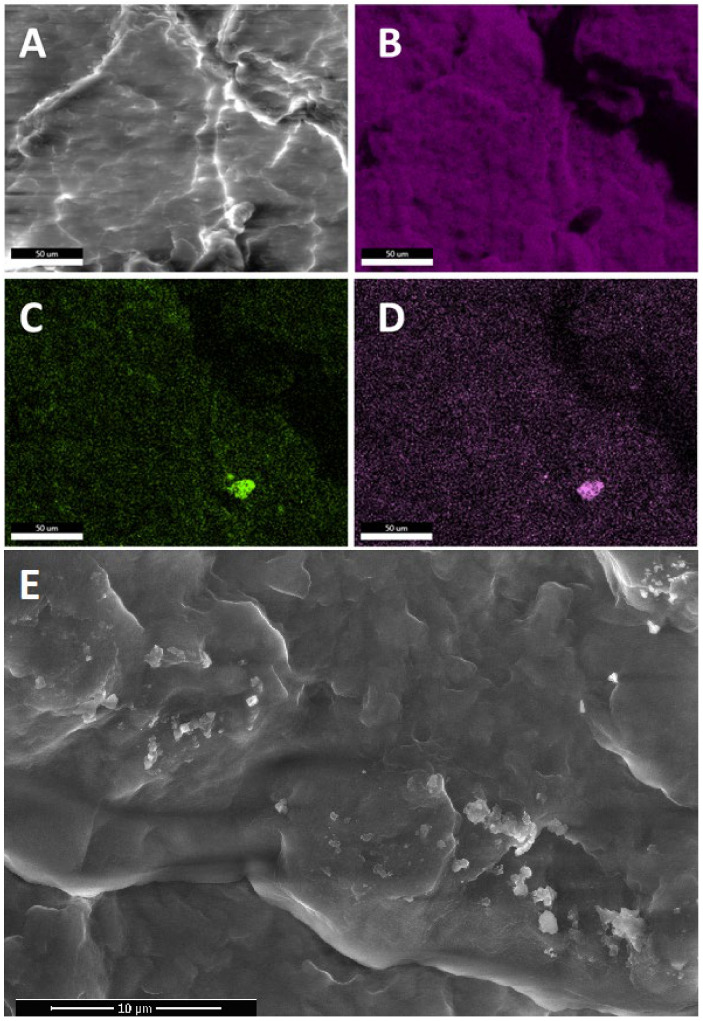
SEM (**A**,**E**) and EDS (**B**–**D**) images of 1% SS-Vi/iPP composite. (**A**)—field of view SEM, (**B**)—carbon EDS, (**C**)—oxygen EDS, (**D**)—Silicon EDS, (**E**)—high resolution SEM.

Scanning electron microscopy combined with energy dispersive spectroscopy was applied to analyze the dispersion/phase separation of the CS additives within the polymer matrix and compatibility of the components. EDS allowed for confirmation of the chemical structure of the observed agglomerates/particles to be of organosilicon origin, as well as detection of agglomerates under the polymer surface. In the study of organosilicon-modified polymer systems, microscopic analysis coupled with X-ray spectroscopy (EDS) shows unique advantages. Very often, during assessment of the homogeneity of the sample, the interpretation of the scanning image alone may be ambiguous, as the morphology of the matrix polymer itself may hinder detection of the particles of the additive. The use of elemental mapping allows us to obtain unambiguity in the interpretation of the observed images and to avoid errors. The oxygen and silicon EDS maps highlight the presence of CS particles in a similar manner, while carbon maps are shadowed in the areas corresponding to CS presence as these compounds have significantly lower carbon content (as a mass percentage) when compared to iPP (see Figure 2B–D and Figure 3B–D). The conducted research allowed us to unequivocally state which of the modifiers are well-dispersed or even dissolved in the polymer matrix and which, despite mixing and dilution, still do not show satisfactory interaction with the matrix.

The analysis of the obtained materials revealed that for most of the systems prepared, the CS additives exist in both nano- and microdispersed states, with a varying fraction of the latter, depending on the additive structure and loading. On the basis of this observation, the term ‘nanocomposite’ should be used carefully, especially when CS compounds are used in high concentration (many literature reports show materials with 3% up to 20% loading). Tang et al. reported how, due to limited compatibility with iPP and iPP-g-MA, octaisobutylsilsesquioxane (*i*Bu_8_SSQ) underwent migration towards the polymer surface and secondary self-aggregation during the composite annealing, despite the use of a high-shear melt-blending method for preparation of the composite [55]. Brabender internal mixer was utilized, which may be considered one of the most effective means for preparation of low amounts of highly dispersed and thoroughly mixed samples of thermoplastic materials and to study the components miscibility. From a practical point of view, it proves that at high loadings, SSQs and iPP may not form stable compositions with CS additives remaining in the state of highly dispersed (nano)particles, even if proper compounding procedures are applied.

**Figure 4:**
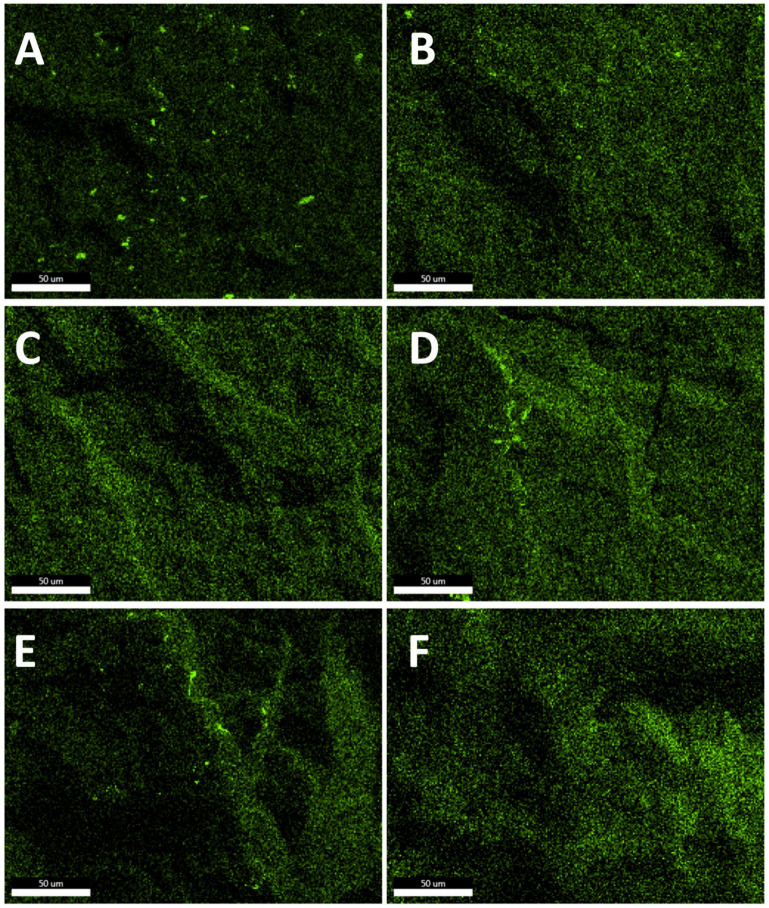
Oxygen EDS images of 1% CS/iPP composites. (**A**)—SS-Glycidyl, (**B**)—SS-Limonene, (**C**)—*i*Bu_7_SSQ-3OH, (**D**)—*i*Bu_7_SSQ-Cl, (**E**)—*i*Bu_7_SS-Vi, (**F**)—*i*Bu_7_SS-H.

**Figure 5:**
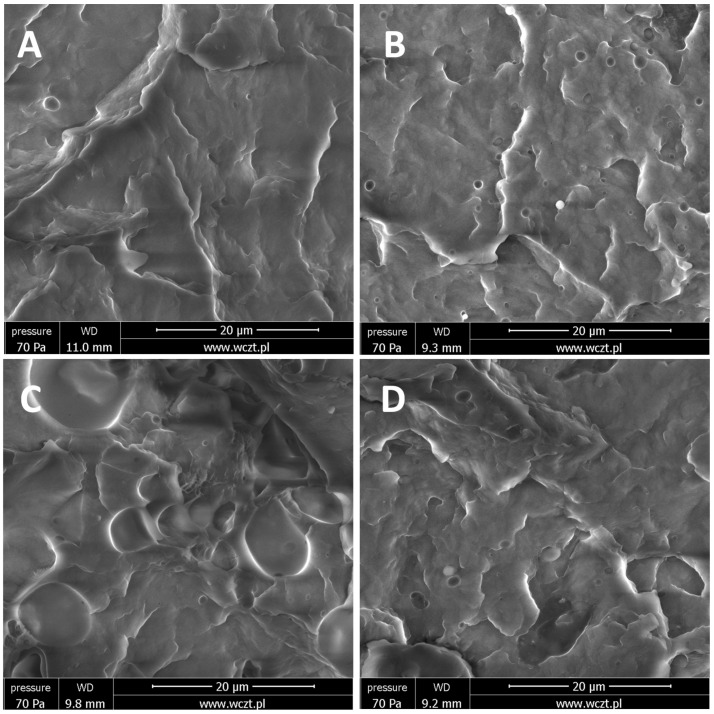
SEM images SS-Glycidyl/iPP composites. (**A**)—0.1%, (**B**)—0.25%, (**C**)—0.5%, (**D**)—1%.

In general, the observed dispersion of the CS additives was considerably better than for PE-based composites containing similar additives, as studied in our previous works [31,32]. It can be explained on the basis of a higher Hildebrandt solubility parameter of iPP than that of PE [56] (or the dispersive parameter according to Hansen model [57]), which better matches the slightly polar character of silsesquioxanes and spherosilicates, induced by the presence of an electronegative oxygen atom. An important work on this subject has been reported by Milliman et al. [58]. Also, as expected, when compared to SS-Vi (Figure 3) or SS-Glycidyl (Figure 4A), the alkylated additives (SS-Limonene and isobutyl SS/SSQ compounds, Figure 4B–F) showed much better dispersion when compared to, as the alkyl substituents provide higher compatibility with iPP than the ether-type glycidyl group or the small vinyl group, providing little steric hindrance for the polar Si-O-Si framework. At the highest loading (1%), all the additives showed some tendency towards agglomeration (Figure 4), which was clearly visible at masterbatch concentrations of all compositions, while further dilution facilitated improved dispersion, as less multimicron-sized aggregates are visible (see Supplementary Information). It was unequivocally found that the crystalline derivative SS-Vi does not show the dispersibility in polypropylene, similarly to SS-H/PE system studied earlier (see Figure 2 and Figure 3). The SS-Glycidyl derivative, although under normal conditions a highly viscous liquid, does not tend to disperse, forming vesicle structures inside the matrix (Figure 5). The limited compatibility of this derivative with PP is already visible at the lowest concentration (0.1%).

The effect of alkyl substituent bulkiness on the dispersion and compatibility of SSQs with iPP was well-presented by Fina et al. and discussed in the terms of SSQ particles acting as nucleants for the growth of iPP [36]. The loading of CS additives had an impact on the dispersion level; as for the lower loadings, less agglomerates were visible, and the obtained materials were more suitable for the term ‘nanocomposites’ as the additive was difficult to observe under SEM or visible mostly as sub-micron particles.

### 3.3. Thermal Analysis Results

Thermal effects for compositions were measured by differential scanning calorimetry (DSC) and thermogravimetric analysis (TGA). The DSC measurements allowed us to determine the effect of the CS additives on the crystallization behavior of the obtained materials, while TG analyses were made to assess the impact of these compounds on the thermal stability of the compositions. For DSC, all the analyzed compositions showed increased crystallization temperatures when compared to neat iPP (see Table 2), which presents their nucleating properties. Interestingly, the highest T_c_ were recorded for SS-Glycidyl/SS compositions, where the additive did not show any crystallization properties in the temperature range of iPP melting and crystallization. Therefore, the accelerated nucleation must be induced by iPP-chains–SS-additive interaction, possibly by the increased void volume or reduced polymer melt viscosity (see Section 3.7) giving polymer chains more freedom for organizing into spherulites (self-nucleating) [59]. SS-Limonene showed comparably good nucleating properties; however. this additive was proven to polymerize under the temperatures of polymer processing (above 100 °C), and the explanation cannot be based on the solvent-like action of the additive resulting in the viscosity reduction, but rather on the nucleating effect of nanoparticles thereof formed upon heat-induced polymerization. Moreover, all the compositions were characterized by a slightly increased T_m_ point (by 1–3 °C), which may be linked to formation of larger polymer spherulites. Butola et al. observed that octamethyl- and octaphenylsilsesquioxanes affected the T_m_ in the range of 1 °C (besides one odd result) over a wide range of loadings (0.1–10% *w*/*w*), and this subtle change may be attributed to poor miscibility of SSQ/iPP systems and poor SSQ–iPP interaction due to low compatibility of the chosen SSQ compounds [60]. The pristine iPP was characterized by a single melting peak of 162.7 °C, rather common for ɑiPP. On the other hand, samples containing either SS-Vi or any of the *i*Bu_7_SS/*i*Bu_7_SSQ additives showed a small, residual endothermic peak at around 150 °C, associated with melting of βiPP and visible only during the first heating. It proves that these additives show a mild β-nucleating character, being revealed during rapid sample cooling; however, during slow cooling (the DSC measurements being recorded at 5 °C/min heating/cooling rate), the β phase either does not form or it recrystallizes into ɑ phase, as β phase is characterized by higher growth rate, but lower stability [61]. This β-nucleating effect, however, was not significant enough to give the CS/iPP composites the traits of typical βiPP materials (see Section 3.4) [62]. For comparison, Pracella et al. also observed formation of βiPP upon addition of octaisobutylsilsesquioxane when studying iPP composites with octaalkyl SSQs, as well as provided micrographs clearly presenting the process of spherulite growth on the surface of SSQ particles [63]. Moreover, Barczewski et al. reported a novel type of β-nucleating silsesquioxane agent derived from NJSTAR NU-100, the addition of which resulted in over 80% selectivity of βiPP crystallites obtained, which was comparable with action of base NJSTAR NU-100 [64]. Also, for most of the additives, the concentration did not play a very significant role, despite SS-Vi being a less effective nucleating agent at 1% loading due to agglomeration (the highest T_c_ of all SS-Vi/PP compositions), and a similar effect was observed for *i*Bu_7_SS-Vi, while the nucleating action of *i*Bu_7_SSQ-3OH was increasing with the concentration of the additive. The other compounds showed a ‘saturation effect’, where the smallest amount of additive used caused the strongest effect on crystallization temperature, while at higher loadings, the difference was close to negligible. Barczewski et al. observed that addition of SS-Vi and vinylhepta(*iso*butyl)silsesquioxane caused a similar saturation effect, and the increase of T_c_ was comparable to results discussed here [65]. Bouza et al. observed that aminopropylhepta(*iso*butyl)silsesquioxane acted as a nucleating agent at 2%, but at 10% it actually hindered PP crystallization, probably by disturbing the polymer chain packing or the particles of agglomerated additive physically blocking the spherulite growth [66]. According to the study by Chen et al., isobutyl-substituted SSQs increased the T_c_ of iPP by up to 1 °C, and a much more common nucleating agent, 1,3:2,4-bis(3,4-dimethylbenzylidene)sorbitol (DMDBS), was proven a superior nucleant, increasing T_c_ by ~12 °C [67].

For characterization of thermal stability and some mechanisms of thermal degradation of the obtained materials, TGA analysis was performed. The data are collected in Table 3 and presented in Figure S1 (see Supplementary Materials). Interestingly, in all cases, a drop of onset temperature (T_onset_) was observed, which proved the discussed additives ability to reduce thermal stability of the obtained CS/iPP composites. This is contrary to a report by Carniato et al., where *i*Bu_7_SSQ-3OH was shown to have a slightly stabilizing effect on the polymer matrix; however, the additive was tested at a loading exceeding the concentration range applied for this study (3%) [18]; or by Fina, where octamethyl-, octaisobutyl-, and octaisooctylsilsesquioxanes induced a slightly stabilizing effect at high loadings (3% and 10% *w*/*w*) [36]. Bouza et al. also observed reduced stability of the SSQ/iPP system containing aminopropylhepta(*iso*butyl)silsesquioxane [66]. On the other hand, Zhou et al. reported a decrease of thermal stability of octavinylsilsesquioxane/PP composites for physically blended samples [68]. A free radical mechanism may be speculated, that is, formation of free radicals originating from decomposing CS molecules, which undergo intermolecular reactions with iPP chains and accelerate their scission. It may occur on the basis of relatively low Si-C bond energy, resulting in elimination of CS side groups [69]. This is supported by the fact the DTG curves are of a different shape in the onset region (280–300 °C) than that of neat iPP, showing higher decomposition rates than the pristine polymer. The effect was also concentration- and dispersion-dependent for the majority of the studied compounds. For example, SS-Vi (proven to be rather poorly miscible within iPP by SEM-EDS) accelerates iPP degradation the strongest when at 0.1% loading (T_onset_ = 294.4) and then at 1%, (T_onset_ = 290.1). At the lowest loading, the additive is most effectively dispersed, whereas at the higher ones, the saturation effect takes place due to the amount of the additive within the matrix. At the moderate loadings, however, agglomeration tends to slightly lower the effect of the additive, directly lowering the effective contact area between iPP and the CS particles, which are no longer abundant in nanosized form and mostly aggregated. The same observation may be done for SS-Glycidyl forming vesicles of separated additive. At the same time, the other additives tend to accelerate the decomposition of iPP more effectively at higher loadings. Similar conclusions on the correlation between the additive loading and the composite behavior were drawn previously for the CS/PE composites, but on the contrary, a stabilizing effect was observed, which shows a great difference between degradation mechanisms of PE and iPP and the CS composites thereof [31,32]. This suggests that small additions of CS compounds might be helpful for catalysis/promoters of iPP cracking, if pyrolytic recycling of polypropylene-based composites was considered [70].

### 3.4. Mechanical Properties

**Figure 6:**
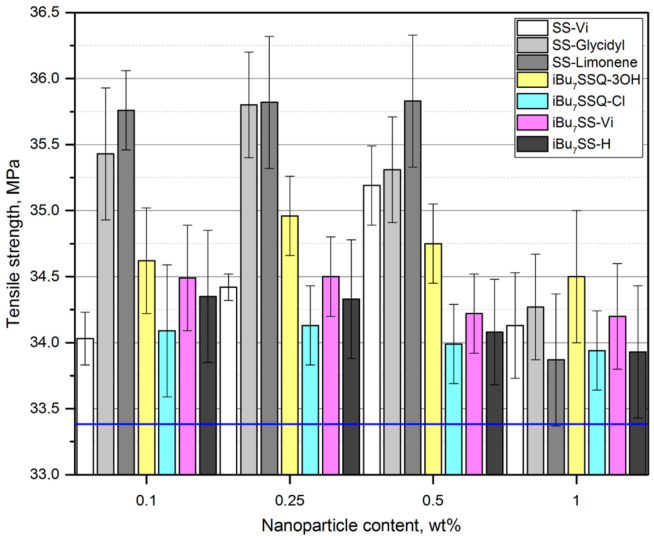
Tensile strength of the CS/iPP composites.

**Figure 7:**
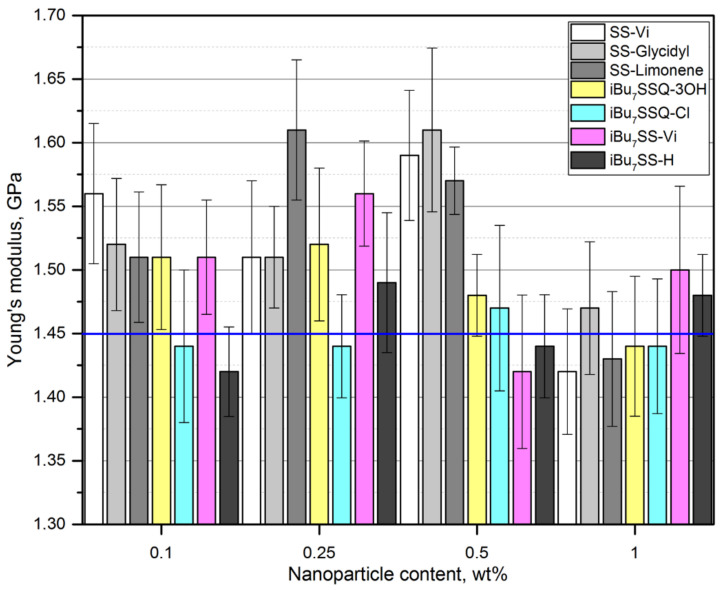
Young’s modulus of the CS/iPP composites.

Mechanical analysis allowed for observation of a reinforcing effect of the additives on the obtained (nano)composites. When studying tensile strength (Figure 6), a general trend was observed for all the additives studied—the results fall into a curve, with the highest values of tensile strength obtained for the composition with lower additive loadings of 0.1–0.5%, the values dropping down for the highest loading (1% *w*/*w*). However, none of the examples showed a drop in tensile strength below that of the reference (neat iPP). When Butola et al. studied SSQ/iPP composites, a similar trend was observed, but for high loadings (up to 5% *w*/*w*), the mechanical parameters were declining below those of the pristine polymer [60]. From this point of view, an optimal loading may be identified for each system where the highest increase of tensile strength was recorded. Additionally, a saturation effect may be observed, especially for SS-Glycidyl, where the maximum increase was obtained already at the 0.1% loading and remained virtually unchanged up to 0.5%. This is due to limited miscibility of the additive with the polymer matrix, which was confirmed by SEM-EDS imaging (critical concentration reached for 0.1% loading). The effect of SS-Limonene may be explained similarly to our previous reports on spherosilicate/polyethylene and spherosilicate/PLA composites, where SS-Limonene was proven to undergo polymerization under high temperatures of polymer processing, which, in case of PE, resulted in formation of a polymer blend of improved mechanical properties [31,33]. For SS-Glycidyl, this explanation is unsuitable as no such polymerization was observed. Rather, molecular level interactions may be considered, where molecules of the additive occupy the polymer void volume and reinforce it on the basis of weak intermolecular interactions between CS and iPP chains, as was speculated for the SS-Pinene/PE system in our previous work. The three isobutyl compounds, i.e., *i*Bu_7_SSQ-Cl, *i*Bu_7_SS-Vi, and *i*Bu_7_SS-H showed very similar patterns due to similarities in their structure. Application of *i*Bu_7_SSQ-3OH resulted in slightly higher improvement of mechanical properties than the other isobutyl derivatives. This result is similar to our previous findings on polyethylene-based composites, where it was proven that *i*Bu_7_SSQ-3OH underwent condensation to a series of amorphous products characterized by better dispersion properties than those of well-defined, cage compounds, like the abovementioned *i*Bu_7_SSQ-Cl, *i*Bu_7_SS-Vi, and *i*Bu_7_SS-H [32]. When studying Young’s modulus (Figure 7), it can be observed that all octaspherosilicate compounds, as well as *i*Bu_7_SSQ-3OH, increased stiffness of the samples at loadings up to 0.5%, with a drop at 1%. *i*Bu_7_SS-Vi showed an improvement of this trait at a concentration up to 0.25%, while *i*Bu_7_SSQ-Cl and *i*Bu_7_SS-H did not impart any statistically relevant change. Reduction of Young’s modulus at higher loadings may be caused by self-aggregation of the additives and lowered interaction with iPP. Additionally, SS-Glycidyl may work as a plasticizing agent, as a slight decrease of the coefficient of friction was observed (see Section 3.5).

**Figure 8:**
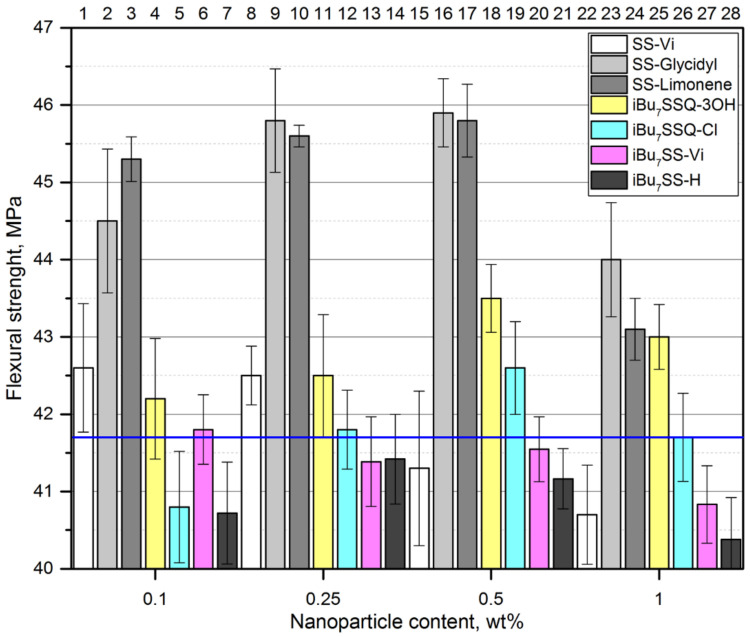
Flexural strength of the CS/iPP composites.

**Figure 9:**
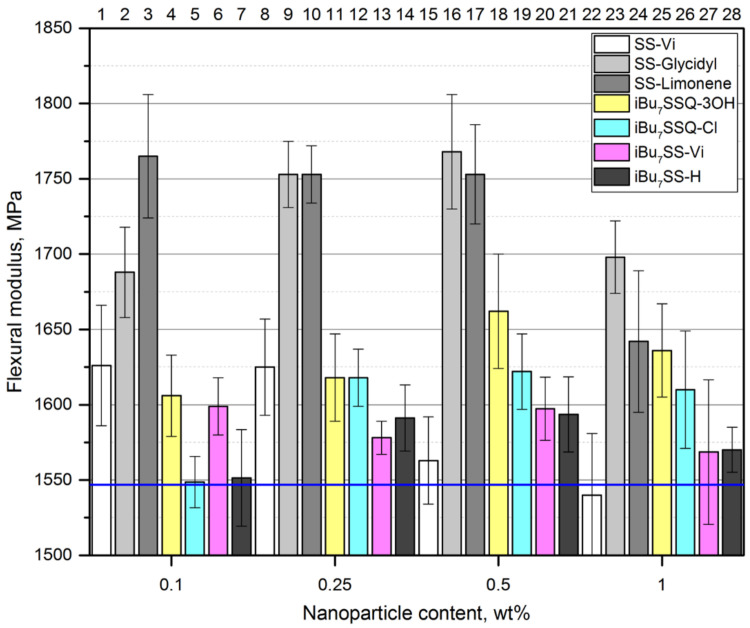
Flexural modulus of the CS/iPP composites.

When compared with tensile strength measurements, a similar trend may be observed for flexural strength (Figure 8) when studying iPP composites containing SS-Glycidyl, SS-Limonene, *i*Bu_7_SSQ-3OH, and *i*Bu_7_SSQ-Cl. The changes of flexural modulus (Figure 9) confirmed the improved toughness of the nanocomposites thereof. However, for the remaining compounds, as well as the lowest loading of *i*Bu_7_SSQ-Cl, the values were oscillating around or below that of the reference. As these compounds (that is, SS-Vi, *i*Bu_7_SSQ-Cl, *i*Bu_7_SS-H, and *i*Bu_7_SS-H) are crystalline solids (which was also visible on SEM as microcrystalline phases, see Section 3.2), these additives may serve as microcrack initiators or stress concentrators, which leads to faster failure of the material under flexural stress, which is known behavior for micrometric-sized fillers [71,72]. Also, Milliman et al. presented how mechanical stress exerted on iPP samples caused debonding of silsesquioxane microparticles from the polymer matrix on the example of Ph_7_SSQ-3OH [59].

### 3.5. Tribological Properties

Tribological properties were studied on the basis of the measurements of the coefficient of friction (µ) of the selected SS/iPP compositions. The additives for these tests were chosen on the basis of the mechanical tests’ performance (see Section 3.4). Two additives were chosen for this study, SS-Limonene and SS-Glycidyl. Moreover, SS-Limonene was selected due to its heat-polymerizing ability, which, in combination with its great dispersion properties, resulted in the most prominent improvement of the mechanical parameters of the studied CS/iPP composites. On the other hand, SS-Glycidyl, which was found to be an oil partially miscible with iPP (on the basis of SEM, see Figure 5), was chosen to be assessed as a potential slip agent. Such additives tend to form a film on the polymer surface or concentrate in the near-surface region of the polymer, significantly changing its physicochemical behavior, while the bulk material may remain unchanged to a certain degree [73]. It was observed that at lower loadings (0.1% and 0.25%), SS-Limonene did not affect the coefficient of friction, while at higher ones, it caused an increase of its value (Figure S2, Supplementary Materials). It supports the results of the mechanical tests that SS-Limonene may be considered a functional additive at lower loadings, however at concentration higher than 0.5% it would not be recommended for applications where material friction is occurring. On the other hand, SS-Glycidyl was found to work as a slip agent, as the obtained mean values of the friction coefficient for the tested compositions were about 5–7% lower than in the case of neat PP (Figure S3, Supplementary Materials). Although the mean values of the friction coefficient were only slightly reduced, the notable drop in the standard deviation thereof suggests that at 1% loading, a transition in motion to smoother sliding [74] occurs.

### 3.6. Vicat Softening Temperature

Vicat softening temperature measurements were performed to assess the impact of the CS additives on the thermomechanical properties of the obtained iPP composites, that is, the temperature of softening under static load (Figure S4, Supplementary Materials). The SS-Limonene/iPP compositions were virtually unaffected when compared to neat PP, contrary to mechanical analysis at ambient temperature, while the other compounds behaved as plasticizers, either due to increased polymer void volume or due to low adhesion of iPP to the (nano)particles thereof, resulting in CS-iPP debonding and accelerated composite failure. At 0.25% loading, SS-Glycidyl/iPP and *i*Bu_7_SS-Vi/iPP were characterized by increased VST values due to CS-iPP reinforcing interactions, which proves the importance of the additives being in a highly dispersed form rather than at high concentration.

### 3.7. Melt Flow Index

**Figure 10:**
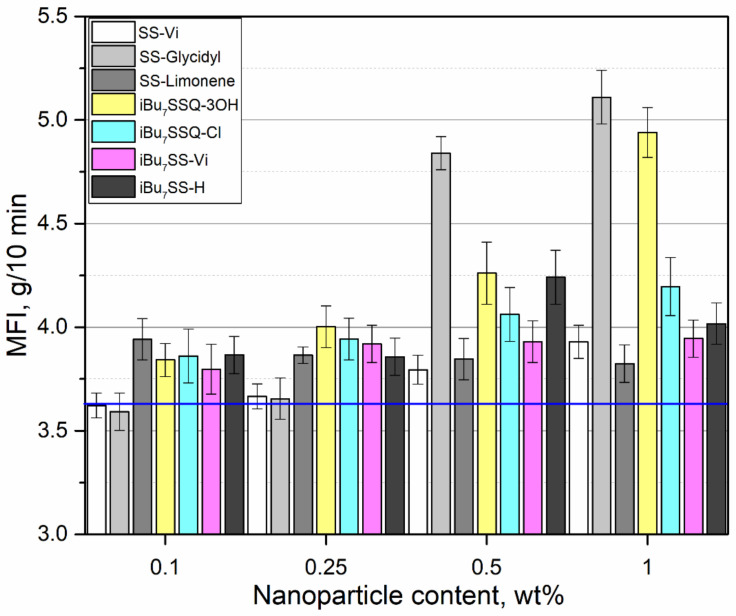
Melt flow index of CS/iPP compositions.

Melt flow index (MFI) measurements were performed to assess the flowability of the obtained materials in their molten form under conditions of static load, which is the most basic measure of the polymer melt viscosity, used as a standard in industrial practice of plastics processing. The results are presented in Figure 10. Interestingly, almost all the compositions showed at least a small increase in the MFI value. The effect could be most easily explained for SS-Glycidyl, which, as mentioned above, is an oily liquid partially miscible with iPP, providing additional lubrication to the flowing polymer, quite similarly to standard lubricants (e.g., silicone oils or synthetic waxes). A notable increase appeared at 0.5% loading, where a possible small phase separation occurred, and at 1% loading, it caused a 40% increase of MFI, which is beneficial for applications such as injection molding, as using a material of correct MFI is crucial for obtaining a product of satisfactory quality. It is a well-known fact that introducing fillers, especially ones of larger aspect ratio (notably reinforcing fibers) causes drastic increase of the flow viscosity [75]. Using proper lubricating additives allows us to minimize this effect. The effect of the other additives may be explained on the basis of intermolecular interactions, where CS molecules diffuse between polymer chains and reduce the polymer chain–chain interaction in favor of chain–CS molecule interaction, which also increases the polymer void volume. This hypothesis has been proposed in a number of other reports on silsesquioxane-containing polymer materials [27,76]. Also, *i*Bu_7_SSQ-3OH showed a strong lubricating effect at 1% loading, which confirms that the amorphous products of its thermal condensation are more susceptible towards interaction with the polymer matrix. Niemczyk et al. also observed that addition of alkyl-substituted octasilsesquioxanes caused increase of MFI (by up to over 80% at 10% loading). Perilla reported that addition of *i*Bu_8_SSQ and Ph_7_SSQ-3OH resulted in reduction of complex viscosity of iPP melt, but the effect was more visible at a very high loading (10% *w*/*w*) [77].

## 4. Practical Implementation

This study has been conducted to verify the applicability of cage siloxane compounds (often referred to as POSS) as functional additives for iPP. Bearing in mind that one of the most important tasks of a scientist is to care for the practical application of research results, we would like to draw attention to the possibility of practical application of research results by specialists working in this particular field. For this reason, a group of silsesquioxane- and spherosilicate-based compounds was selected and tested in a low concentration range to accommodate their relatively high price. The obtained materials were subjected to a comprehensive study of thermal, mechanical, and rheological properties to reveal the potential of CS compounds as functional or processing additives for polyolefins, which is a continuation of our previous work on CS/polyethylene composites. Additionally, it was important to clarify the terminology used in accordance with these systems. It should be noted that there is a clear difference between two types of additives, that is, fillers and modifiers. The application of the first one usually reduces the price of the composite and may bring about a secondary effect of changes in the material, while addition of the latter purposely influences the processing properties of the material. This work was meant to explain why the CS compounds should not be referred to as fillers or nanofillers, as it is misleading for the plastics processing/engineering community.

## 5. Conclusions

A following set of conclusions may be drawn from this study:

1. CS compounds show much better dispersion properties in an iPP matrix when compared to similar compositions prepared in a similar manner with PE serving as a matrix material. It can be explained on the basis of higher Hildebrandt solubility parameter or Hansen dispersive parameter of iPP than that of PE, matching the dipole character of cage siloxanes. It results in better improvement in performance of CS/iPP composites in comparison to similar CS/PE composites.
2. Two factors are critical for obtaining iPP-based nanocomposites containing silsesquioxanes and spherosilicates. One is the chemical structure of the compounds, which should match the character of iPP. The second aspect is dilution of the additive within the polymer-at the highest concentrations tested; most of the studied compounds had a tendency to form aggregates, which reduced their effectiveness as additives.
3. Among the tested CS compounds, functionalized spherosilicate (SS-Glycidyl, SS-Limonene) and silsesquioxane (*i*Bu_7_SSQ-3OH) additives may be considered valuable agents for improving mechanical properties of iPP, mainly tensile and flexural strength, with optimal loading not exceeding 0.5%. Crystalline CS, mainly the remaining silsesquioxanes, did not present beneficial effects on these properties.
4. SS-Glycidyl and *i*Bu_7_SSQ-3OH provide lubricating action, according to MFI, which is beneficial from the point of view of selected polymer processing techniques (e.g., injection molding, melt blowing).
5. CS compounds tend to reduce thermal stability of the obtained iPP compositions thereof (degradation promoters), which is contrary to the behavior observed by us for CS/PE composites studied earlier, and to the behavior of iPP composites containing high loadings of CS and reported in other sources (degradation inhibitors). As a result, they may be considered catalysts for pyrolytic decomposition/recycling of iPP-based materials.

On the basis of the conducted research, we suggest that in the case of polyolefin systems and other polymer systems used in bulk quantities, where no unequivocal effects such as permanence, strength, or cost reduction are observed upon addition of a given compound, the use of the term ‘nanofiller’ in relation to compounds of the CS type should not be considered. Due to agglomeration, the CS additives often do not meet the definition of ‘nano’ fillers (they form polycrystalline agglomerates). Moreover, due to their high cost, they considerably increase the price of the final composition if used in quantities exceeding a fraction of a single percent by mass. This is contradictory to the definition of a filler, as these are usually applied as a significant mass fraction of the composition. Therefore, these compounds should be used at low concentration and selected or designed in such a way that they indeed play a role of (nano)modifiers in order to be considered viable additives for polymer systems, justifying their cost.

## Figures and Tables

**Table 1:** Silsesquioxane and spherosilicate derivatives used in this study.

Name	Abbreviation	Literature Report
Octahydrospherosilicate	SS-H	[49]
Octavinylspherosilicate	SS-Vi	[49] (prepared analogically to SS-H)
Octaglycidylspherosilicate	SS-Glycidyl	[50]
Octalimonenespherosilicate	SS-Limonene	[31]
hepta(*iso*butyl)trisilanol silsesquioxane	*i*Bu_7_SSQ-3OH	[51]
chloropropylhepta(*iso*butyl)silsesquioxane	*i*Bu_7_SSQ-Cl	[52]
Monovinylhepta(*iso*butyl)spherosilicate	*i*Bu_7_SS-Vi	[53]
Monohydrohepta(*iso*butyl)spherosilicate	*i*Bu_7_SS-H	[54]

**Table 2:** Results of DSC analysis of CS/iPP composites.

Sample		T_m_ [°C]	T_c_ [°C]
Neat PP		162.7	117.2
**SS-Vi**	0.1%	164.2 *	119.0
	0.25%	164.1 *	119.3
	0.5%	164.5 *	119.4
	1%	164.2 *	118.6
**SS-Glycidyl**	0.1%	165.1	122.2
	0.25%	165.5	122.6
	0.5%	164.9	124.0
	1%	165.1	124.9
**SS-Limonene**	0.1%	165.0	123.1
	0.25%	164.9	123.2
	0.5%	165.1	124.0
	1%	165.3	124.6
***i*Bu_7_SSQ-3OH**	0.1%	165.2 *	119.9
	0.25%	164.4 *	120.8
	0.5%	164.9 *	121.7
	1%	164.6 *	122.0
***i*Bu_7_SSQ-Cl**	0.1%	163.5 *	119.3
	0.25%	163.3 *	119.7
	0.5%	165.0 *	119.7
	1%	165.0 *	119.9
***i*Bu_7_SS-Vi**	0.1%	164.3 *	119.1
	0.25%	164.7 *	119.2
	0.5%	164.8 *	120.5
	1%	164.3 *	119.3
***i*Bu_7_SS-H**	0.1%	164.6 *	119.4
	0.25%	164.6 *	119.7
	0.5%	165.1 *	119.1
	1%	163.8 *	119.4

* additional, residual β phase melting endotherm observed during the first heating cycle.

**Table 3:** Results of thermogravimetric analysis (air atmosphere).

Sample		T_5%_ [°C]	T_onset_ [°C]	T_DTG_ [°C]
Neat PP		283.3	312.2	341.4
**SS-Vi**	0.1%	274.3	294.4	325.5
	0.25%	282.8	306.1	349.9
	0.5%	280.8	302.2	333.7
	1%	274.7	290.1	321.9
**SS-Glycidyl**	0.1%	279.8	299.3	336.6
	0.25%	283.6	302	351.8
	0.5%	288.6	311.3	360.5
	1%	280.6	304.9	342.7
**SS-Limonene**	0.1%	274.5	293.6	343.8
	0.25%	271.0	297.3	332.8
	0.5%	274.8	295	328.2
	1%	278.4	299.7	336.0
***i*Bu_7_SSQ-3OH**	0.1%	283.9	307.4	348.9
	0.25%	276.5	297.6	342.9
	0.5%	274.5	309.6	333.9
	1%	274.6	297.0	334.6
***i*Bu_7_SSQ-Cl**	0.1%	284.6	306.1	349.1
	0.25%	271.8	289.8	320.5
	0.5%	275.8	294.3	338.8
	1%	279.3	312.2	350.1
***i*Bu_7_SS-Vi**	0.1%	279.8	300.7	344.0
	0.25%	278.7	301.4	329.7
	0.5%	275.5	291.5	328.4
	1%	274.9	296.2	331.4
***i*Bu_7_SS-H**	0.1%	279.5	301.4	339.8
	0.25%	277.3	296.4	334.7
	0.5%	271.0	281.9	308.4
	1%	278.2	302.4	341.7

## Supplementary Materials

The following are available online at https://www.mdpi.com/article/10.3390/polym13132124/s1, Figure S1: TGA thermograms of CS/iPP compositions, Figure S2: Coefficients of friction for SS-Limonene/iPP composites, Figure S3: Coefficients of friction for SS-Glycidyl/iPP composites, Figure S4: Vicat softening temperatures of the obtained CS/iPP composites, NMR and FT-IR spectra of the obtained CS compounds; SEM and EDS images of the obtained CS-iPP composites.
