# Supplementary material for: Why POSS-Type Compounds Should Be Considered Nanomodifiers, Not Nanofillers—A Polypropylene Blends Case Study
Source: Polymers (Basel). 2021 Jun 28;13(13):2124. doi: 10.3390/polym13132124 (PMC8271478; doi:10.3390/polym13132124)

Supplementary Materials for

Why POSS-type compounds should be considered nanomodifiers, not nanofillers - a polypropylene blends case study

Dariusz Brząkalski[1], Robert E. Przekop[2*], Bogna Sztorch[2], Miłosz Frydrych[1], Daria Pakuła[1], Marek Jałbrzykowski[3], Grzegorz Markiewicz[3], Bogdan Marciniec[1,2*]

[1] Faculty of Chemistry, Adam Mickiewicz University in Poznan, Poznan, Poland
[2] Centre for Advanced Technologies, Adam Mickiewicz University in Poznan, Poznan, Poland
[3] Bialystok University of Technology, Faculty of Mechanical Engineering, Wiejska 45 C, 15-351 Bialystok, Poland
* Correspondence: robert.przekop@amu.edu.pl (R.P.); bogdan.marciniec@amu.edu.pl (B.M.)

Table of contents:

1. Table of isolated compounds: .... - 2 -

2. Additional Figures 4

3. Spectroscopic characterization of the products: 7

3. SEM and EDS images of the SS/PP composites 23

1. Table of isolated compounds:

Structure	Compound #	Code
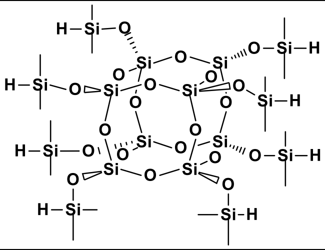	**1**	SS-H
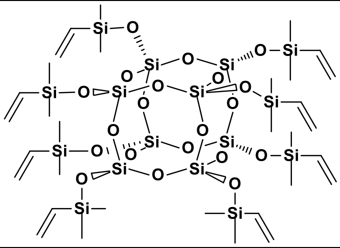	**2**	SS-Vi
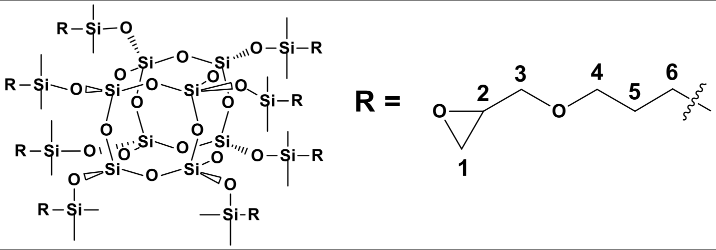	**3**	SS-Glycidyl
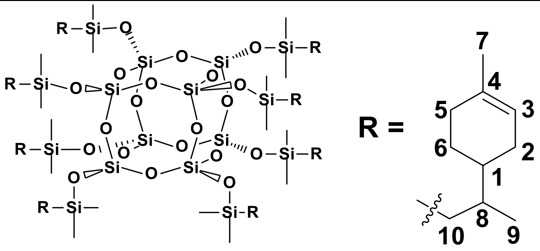	**4**	SS-Limonene
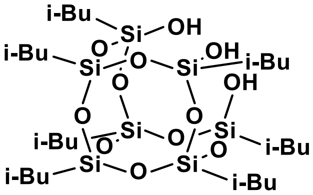	**5**	iBu_7SSQ-3OH
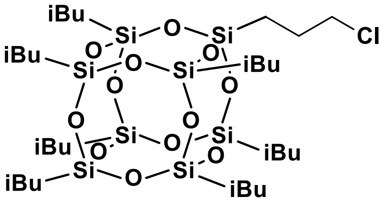	**6**	iBu_7SSQ-Cl
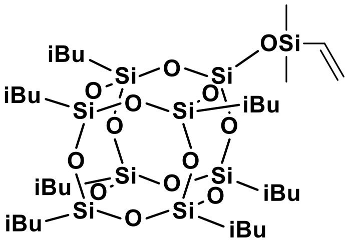	**7**	iBu_7SS-Vi

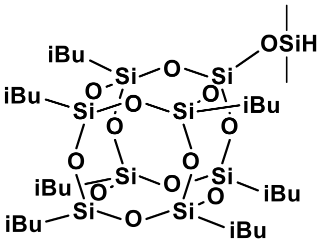	8	iBu_7SS-H

2. Additional Figures

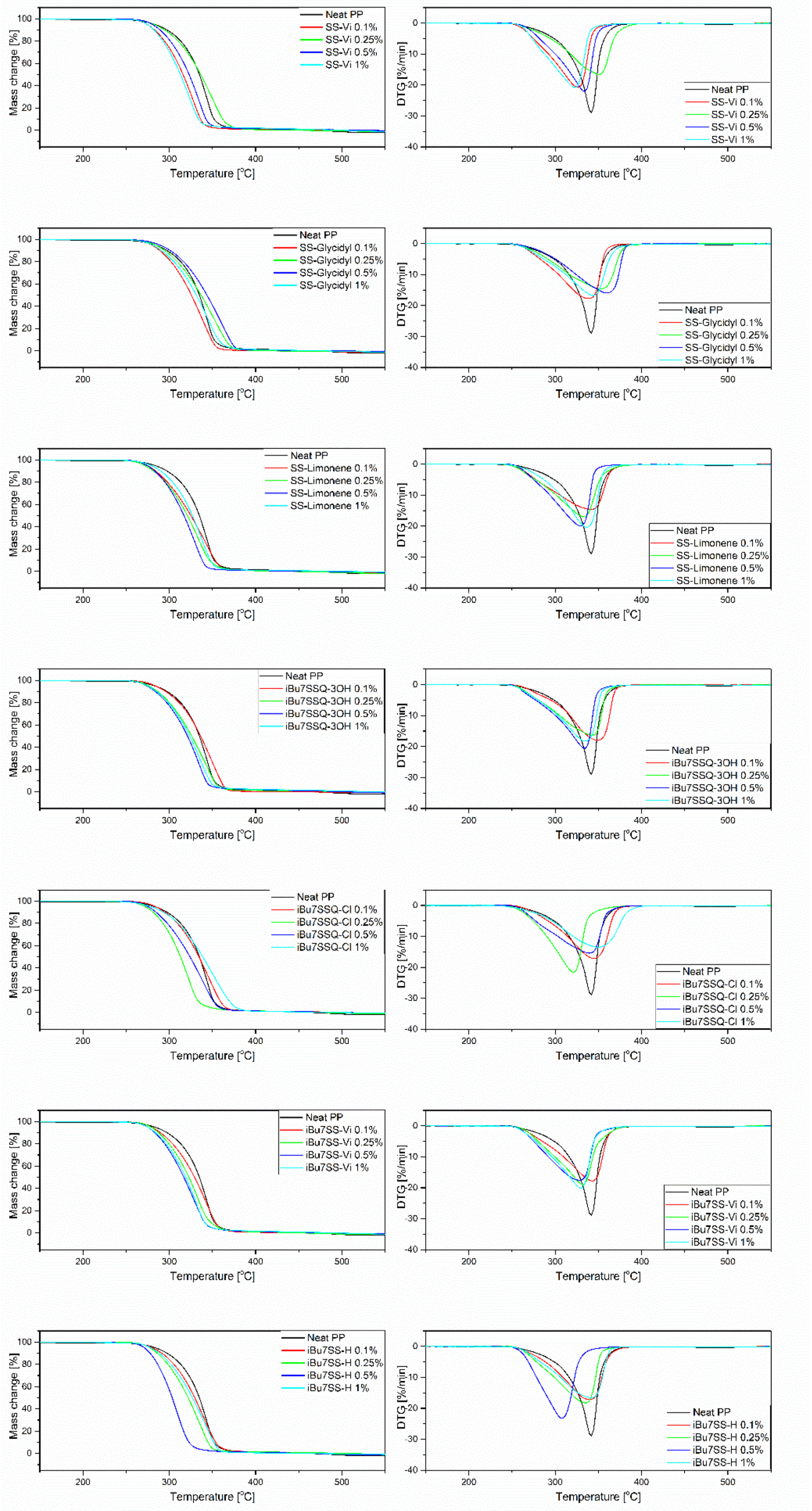

Figure S1: TGA thermograms of CS/iPP compositions.

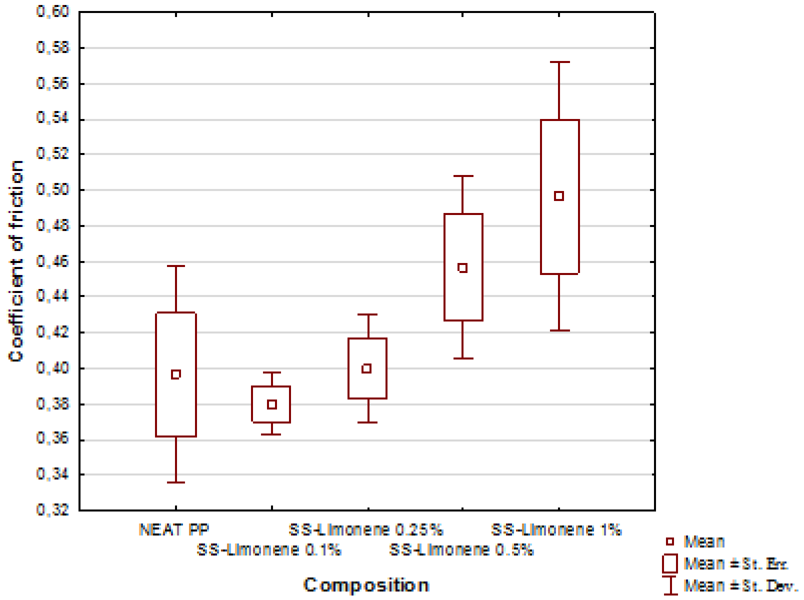

Figure S2. Coefficients of friction for SS-Limonene/iPP composites.

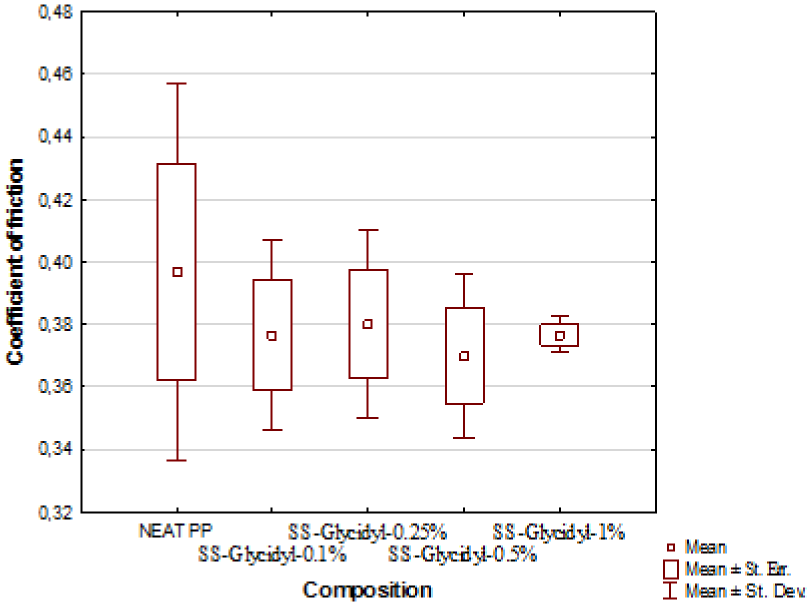

Figure S3. Coefficients of friction for SS-Glycidyl/iPP composites.

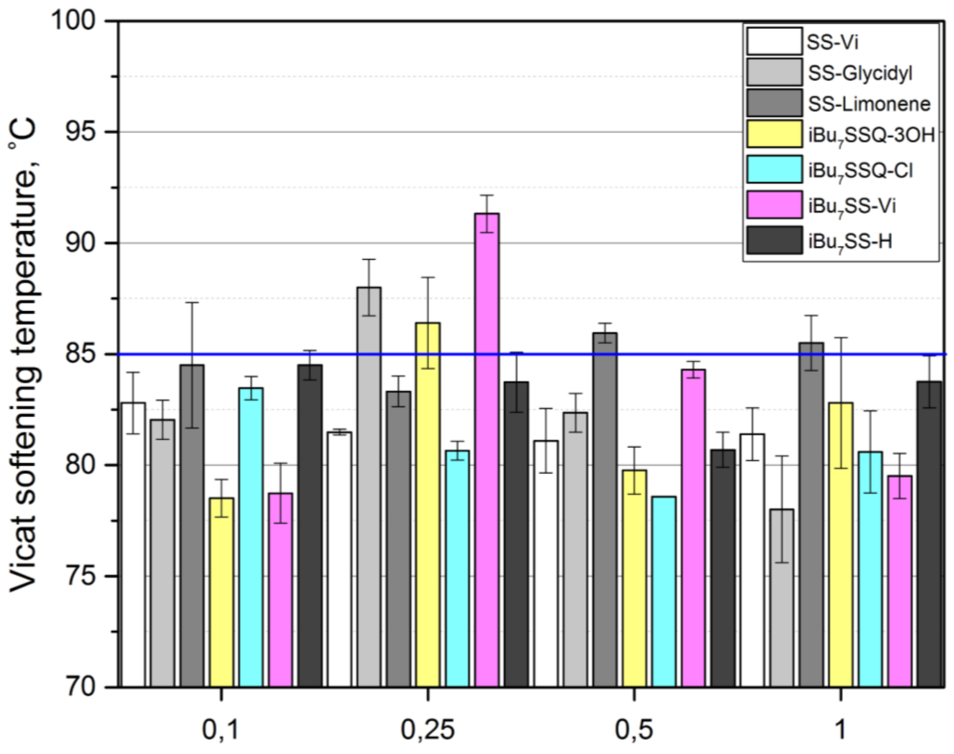

Figure S4. Vicat softening temperatures of the obtained CS/iPP composites.

3. Spectroscopic characterization of the products:

1,3,5,7,9,11,13,15-hepta(dimethylsiloxy)-pentacyclo[9.5.1.1^{3,9}.1^{5,15}.1^{7,13}]octasiloxane (1)

^{1}H NMR (400 MHz, $CDCl_3$): δ (ppm) = 4.73 (sep, J=2.8Hz, 8H, SiH), 0.26 (d, J=2.8Hz, 48H, $SiMe_2$);
^{13}C NMR (101 MHz, $CDCl_3$): δ (ppm) = 0.23 ($SiMe_2$);
^{29}Si NMR (79,5 MHz, $CDCl_3$): δ (ppm) = -1.41 ($SiMe_2$), -108.68 (core).
FT-IR (ATR): 2965, 2905, 2141, 1254, 1069, 889, 834, 769-726, 650, 628, 541.

^{1}H NMR ($CDCl_3$, 400MHz):

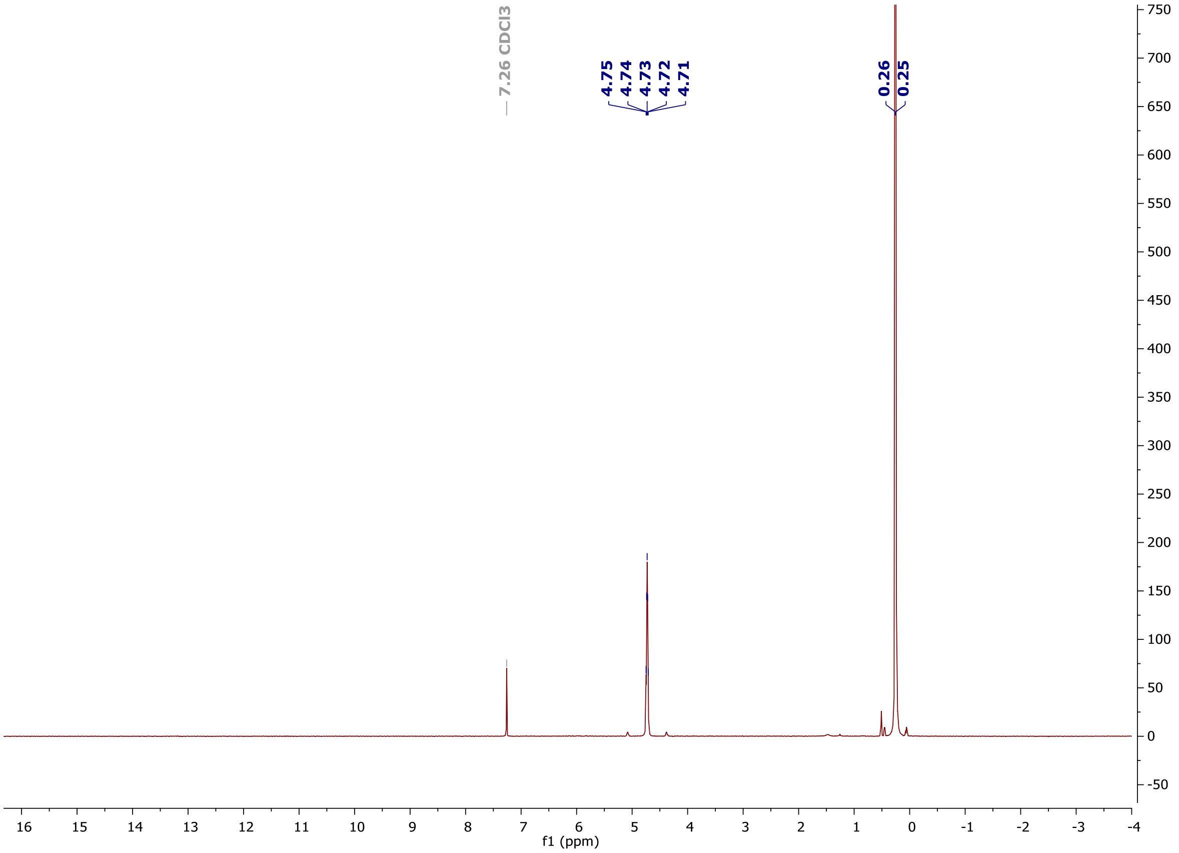

^{13}C NMR (101 MHz, $CDCl_3$)

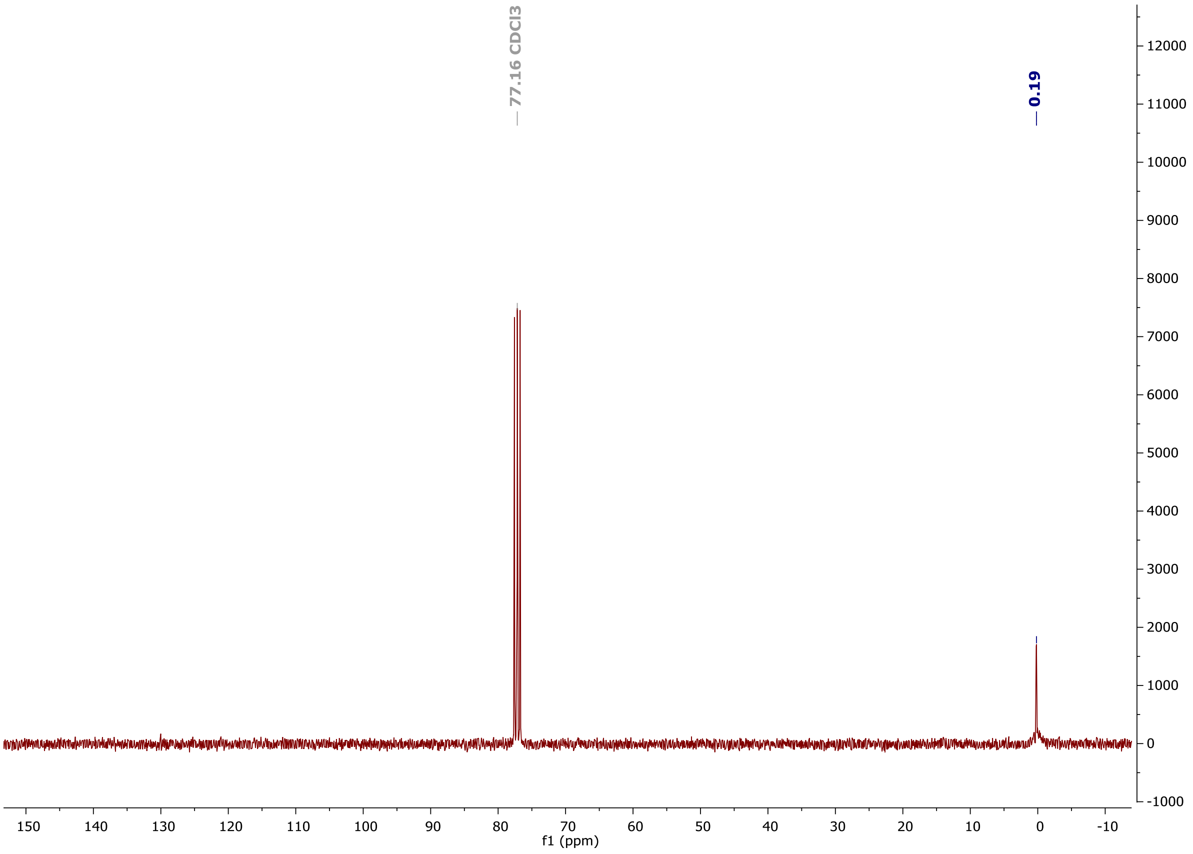

^{29}Si NMR (79,5 MHz, $CDCl_3$)

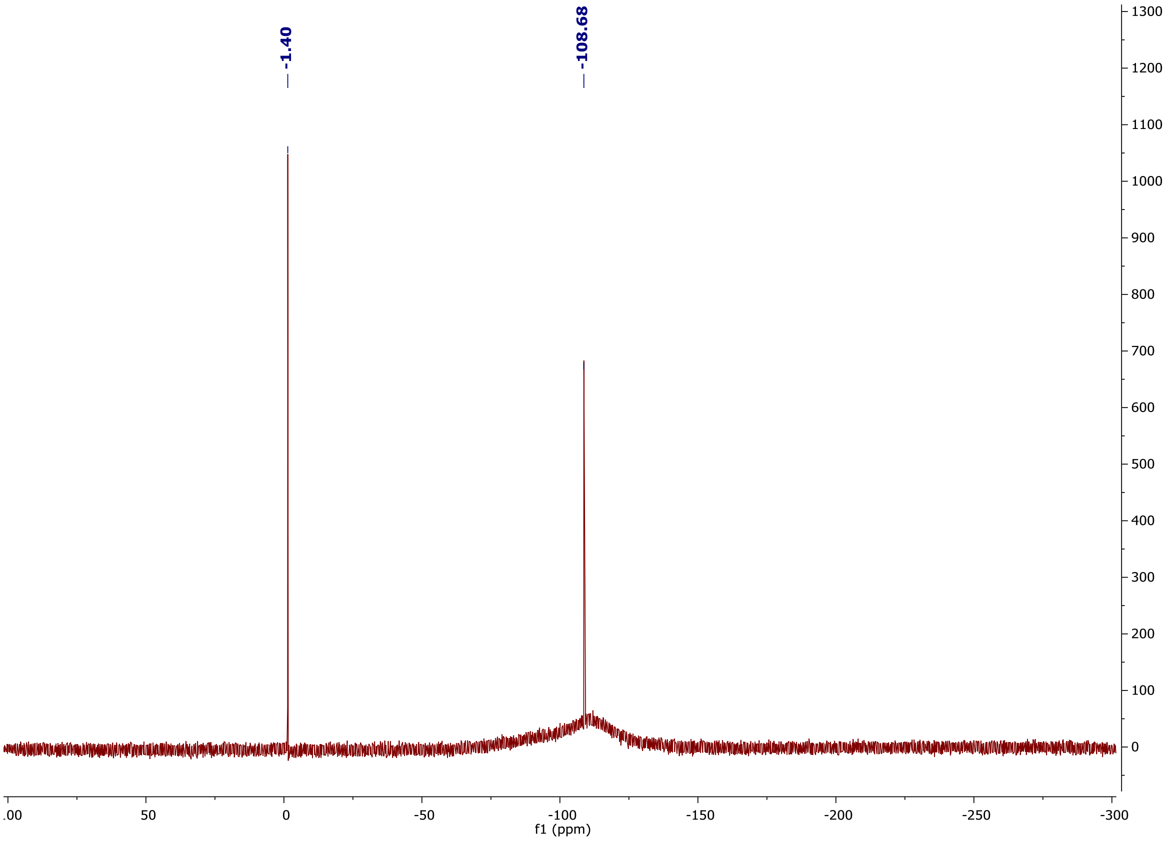

1,3,5,7,9,11,13,15-octa(dimethylvinylsiloxy)-pentacyclo[9.5.1.1^{3,9}.1^{5,15}.1^{7,13}]octasiloxane (2)

^{1}H NMR (400 MHz, $CDCl_3$): δ (ppm) = 6.13 (dd, J_1=20.2Hz, J_2=14.9Hz, 8H, -C<u>H</u>=CH_2), 5.96 (dd, J_1=14.9Hz, J_2=4.0Hz, 8H, -CH=C<u>H_2</u>), 5.79 (dd, J1=20.2Hz, J2=4.0Hz, 8H, -CH=C<u>H_2</u>), 0.21 (s, 48H, $SiMe_2$);
^{13}C NMR (101 MHz, $CDCl_3$): δ (ppm) = 138.09, 132.61 (-CH=CH_2), -00.06 ($SiMe_2$);
^{29}Si NMR (79,5 MHz, $CDCl_3$): δ (ppm) = 0.51 ($SiMe_2$), -109.14 (core).
FT-IR (ATR): 3068, 3027, 2988, 2962, 1603, 1408, 1276, 1081, 1002, 967, 775, 568.

^{1}H NMR ($CDCl_3$, 400MHz):

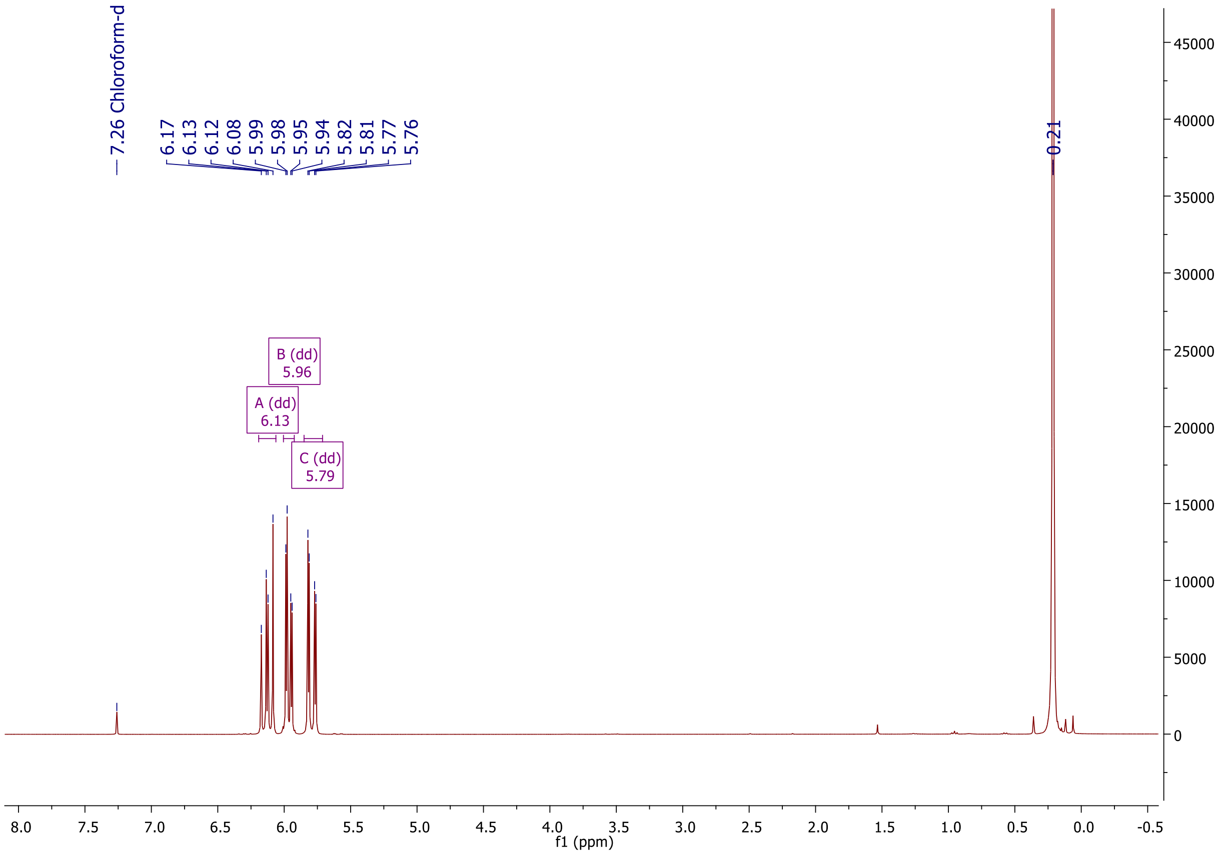

[13]**C NMR** *(CDCl$_3$, 101 MHz):*

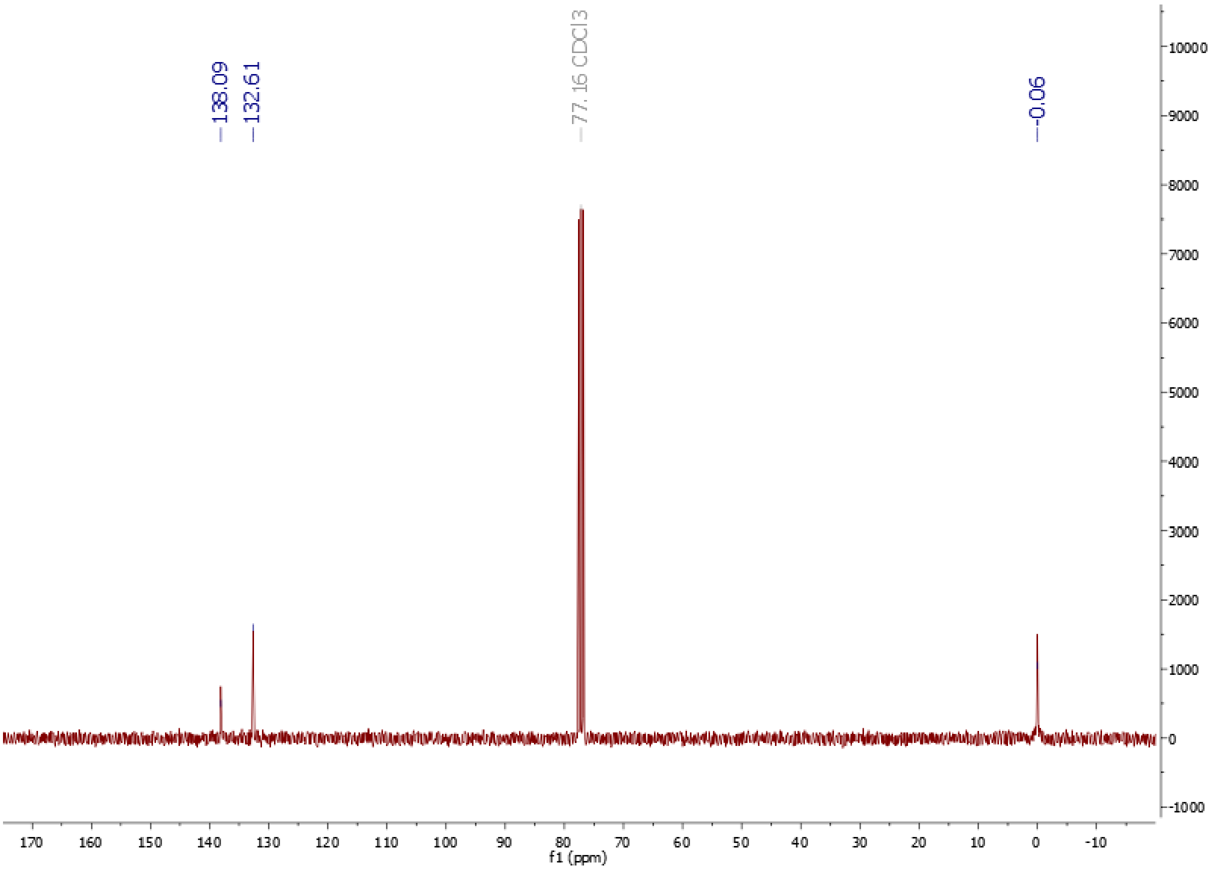

[29]**Si NMR** *(CDCl$_3$, 79.5 MHz):*

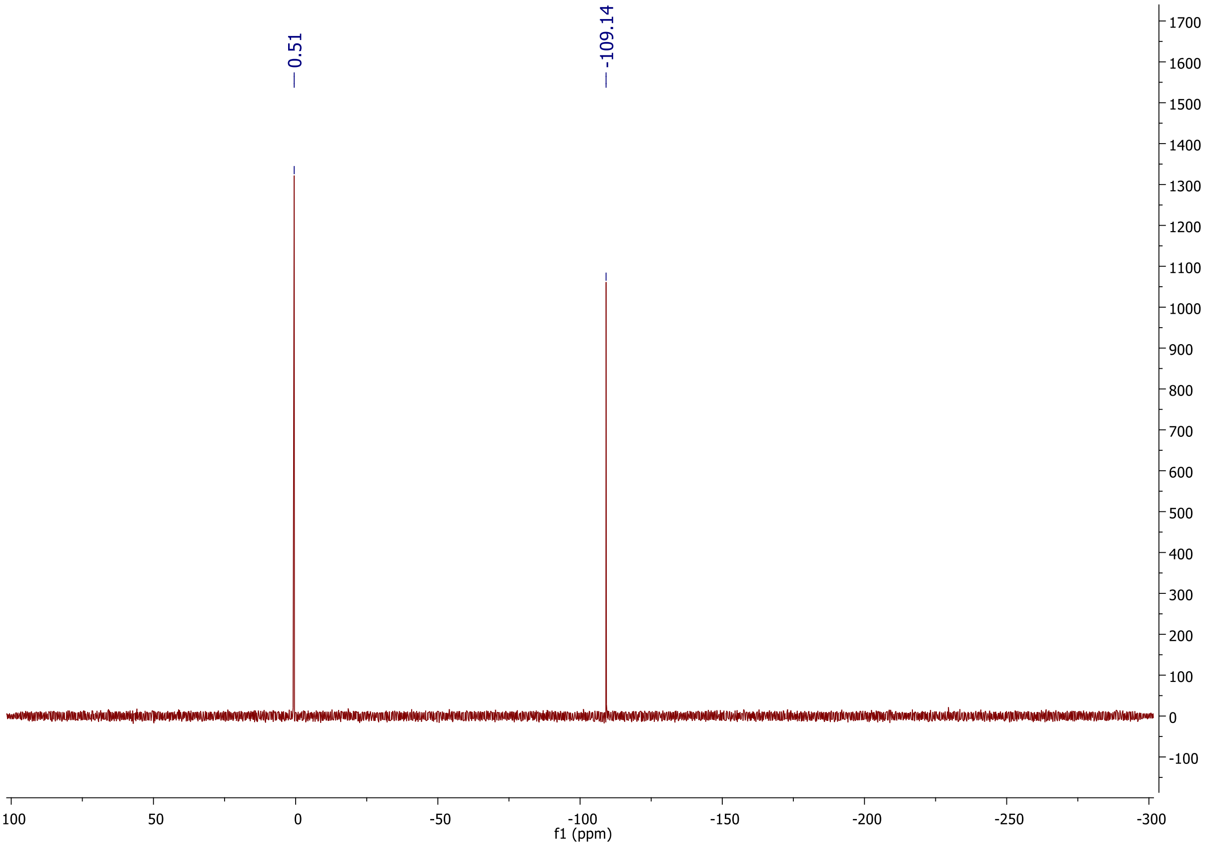

1,3,5,7,9,11,13,15-octa((3-glycidoxypropyl)dimethylsiloxy)-pentacyclo[9.5.1.1^{3,9}.1^{5,15}.1^{7,13}]octasiloxane (3)

^{1}**H NMR** (400 MHz, $CDCl_3$): δ (ppm) = 3.70-3.67 (m, 8H position 3), 3.48-3.40 (m, 16H, position 4), 3.39-3.36 (m, 8H, position 3), 3.14-3.11 (m, 8H, position 2), 2.79-2.77 (m, 8H, position 1), 2.60-2.59 (m, 8H, position 1), 1.65-1.60 (m, 16H, position 5), 0.62-0.58 (m, 16H, position 6), 0.14 (s, 48H, $SiMe_2$);
^{13}**C NMR** (101 MHz, $CDCl_3$): δ (ppm) = 74.23 (position 4), 71.58 (position 3), 50.96 (posirion 2), 44.44 (position 1), 23.31 (position 5), 13.80 (position 6)(glycidoxypropyl\l group), -0.25 ($SiMe_2$);
^{29}**Si NMR** (79,5 MHz, $CDCl_3$): δ (ppm) = 12.92 ($SiMe_2$), -109.09 (core).
FT-IR (ATR): 3053, 2996-2870, 1479-1390, 1343, 1253, 1160-1070, 909-729, 631, 546.

^{1}*H NMR* *($CDCl_3$, 400MHz):*

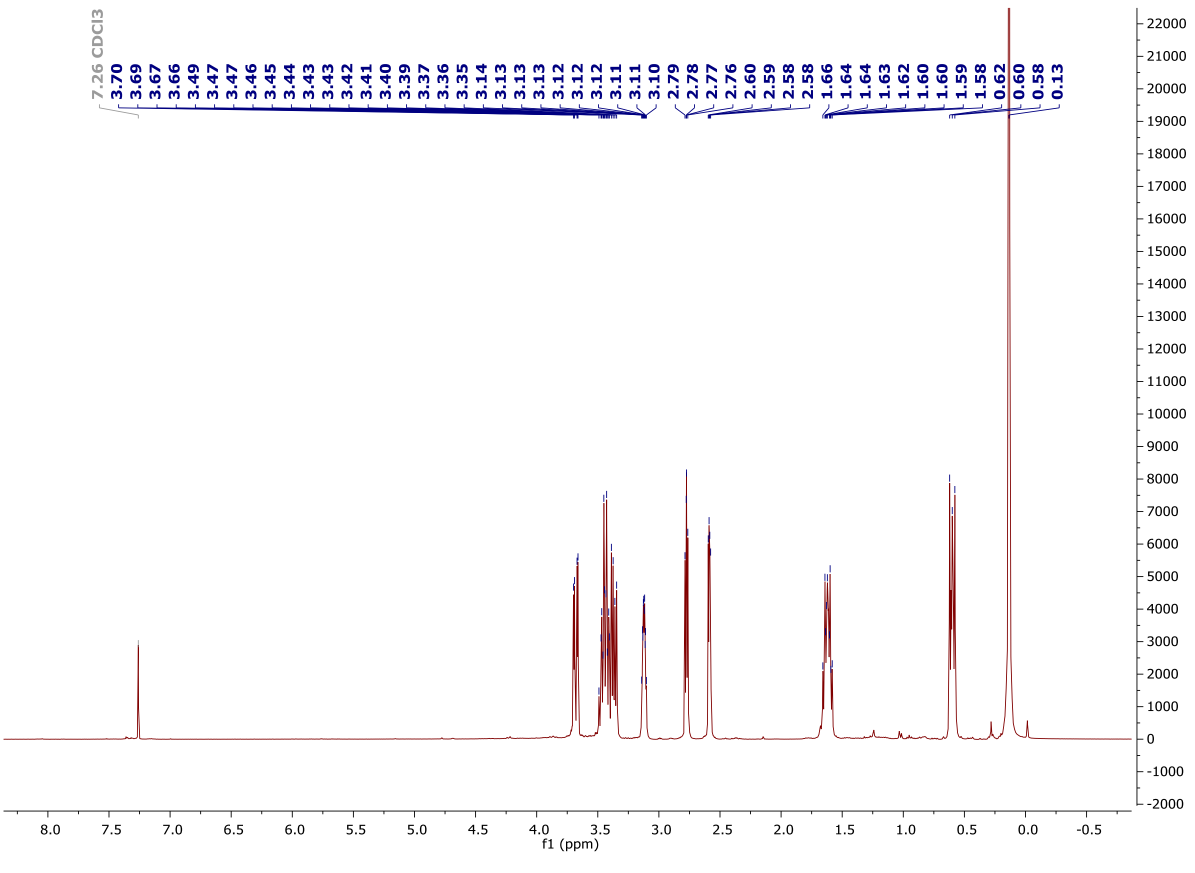

***13*C NMR** (CDCl$_3$, 101 MHz):*

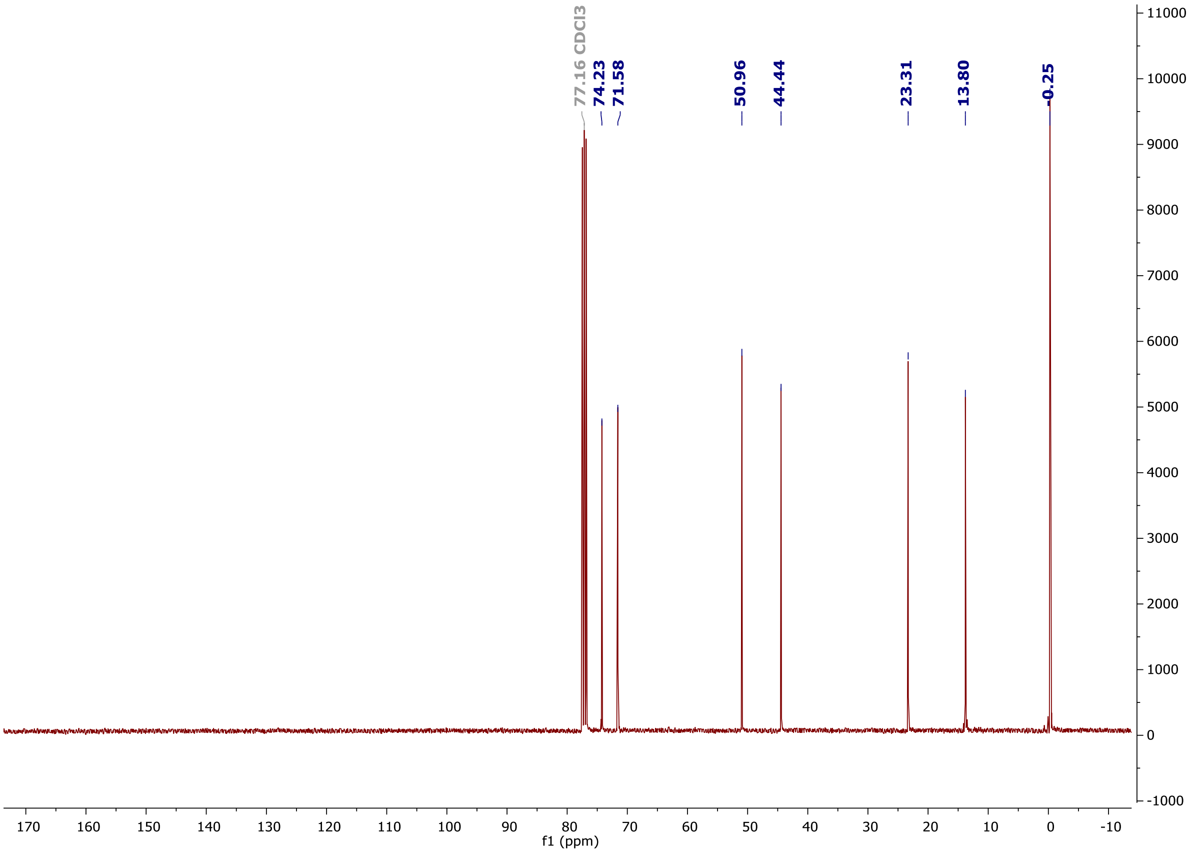

***29*Si NMR** (CDCl$_3$, 79.5 MHz):*

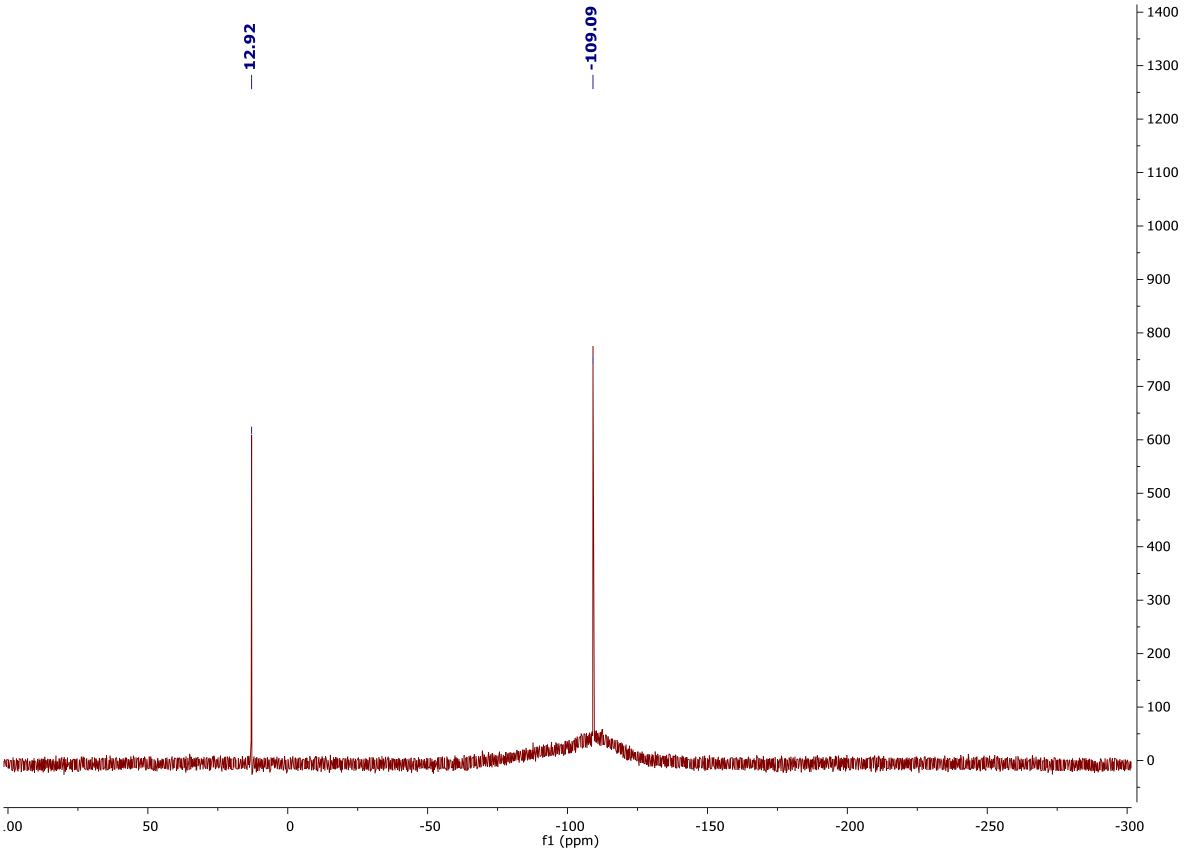

1,3,5,7,9,11,13,15-octa(dimethyl((2-(4-methylcyclohex-3-en-1-yl)propyl)silyl)-pentacyclo[9.5.1.1^{3,9}.1^{5,15}.1^{7,13}]octasiloxane (4)

^{1}H NMR (400 MHz, $CDCl_3$): δ (ppm) = 5.36 (s, 8H, position **3**), 2.00-1.87 (m, 24H, positions **1, 2, 5**), 1.78-1.56 (m, 24H, positions **2, 5, 8**), 1.63 (s, 24H, position **7**), 1.34-1.18 (m, 16H, position **6**), 0.90 (d, 24H, position **9**), 0.75-0.69 (m, 8H, position **10**), 0.52-0.44 (m, 8H, position **10**), 0.15 (s, 48H, $SiMe_2$);
^{13}C NMR (101 MHz, $CDCl_3$): δ (ppm) = 133.96, 121.28, 121.26, 41.48, 41.33, 33.08, 32.93, 31.19, 31.12, 28.80, 28.24, 26.71, 25.71, 23.64, 23.63, 22.87, 22.48, 19.52, 19.22, 0.83, 0.72, 0.66;
^{29}Si NMR (79,5 MHz, $CDCl_3$): δ (ppm) = 12.78 ($SiMe_2$), -109.10 (core).
FT-IR (ATR): 2980-2867, 1252, 1169-1069, 869-734, 549.

*^{1}**H NMR** ($CDCl_3$, 400MHz):*

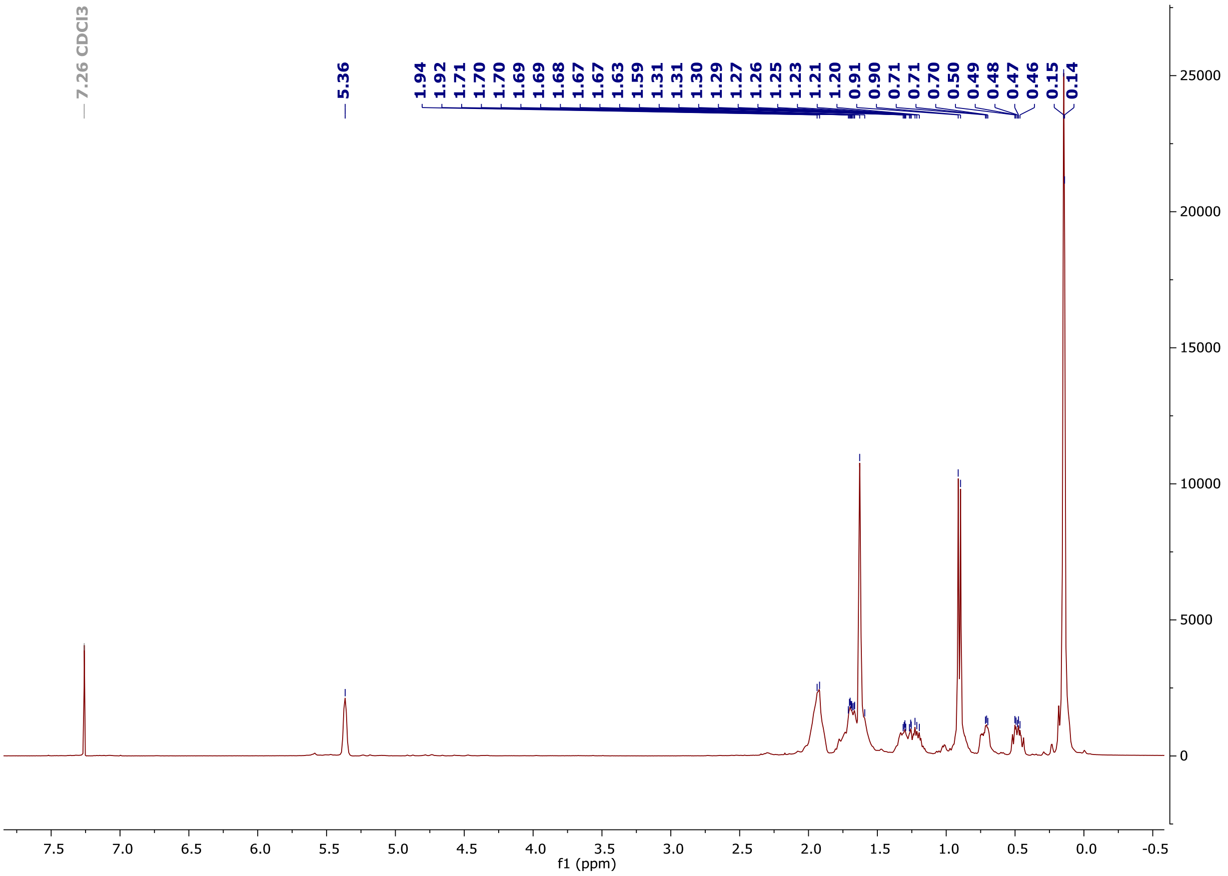

^{13}C NMR *($CDCl_3$, 101 MHz):*

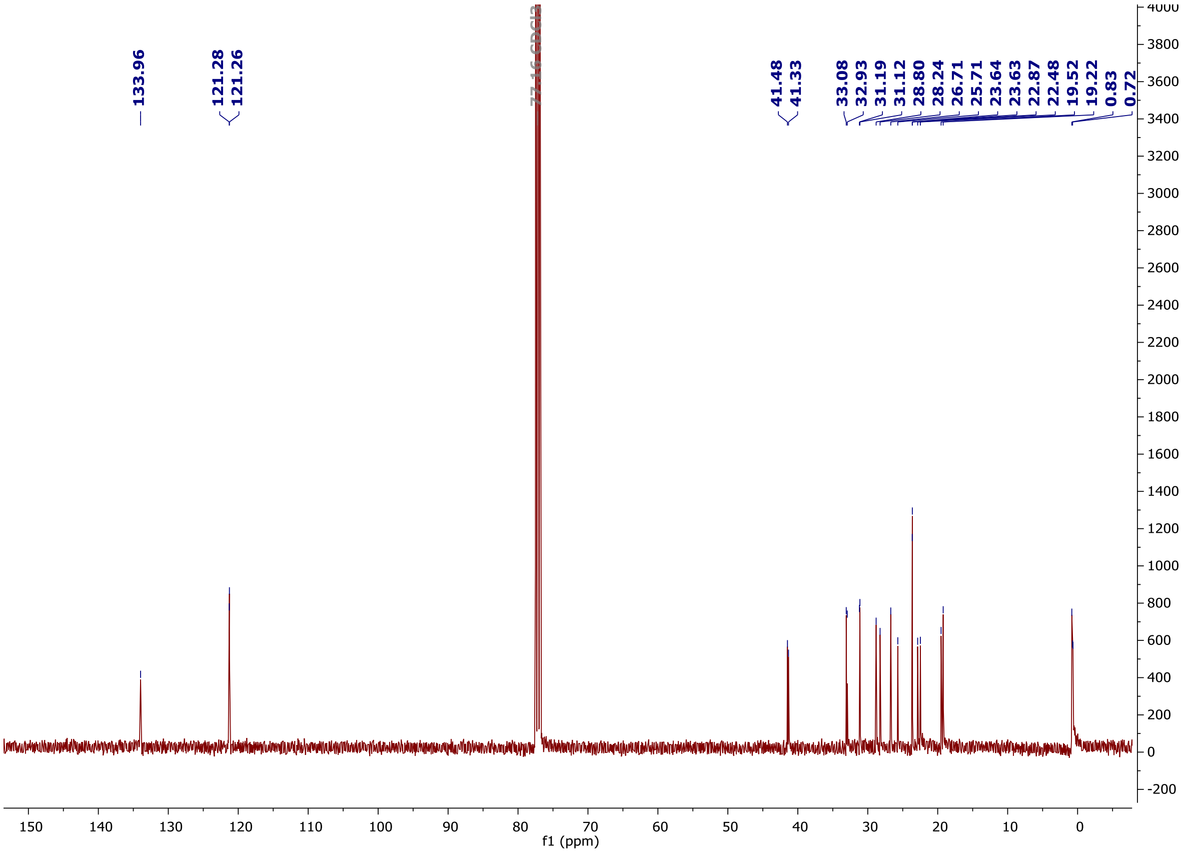

^{29}Si NMR *($CDCl_3$, 79.5 MHz):*

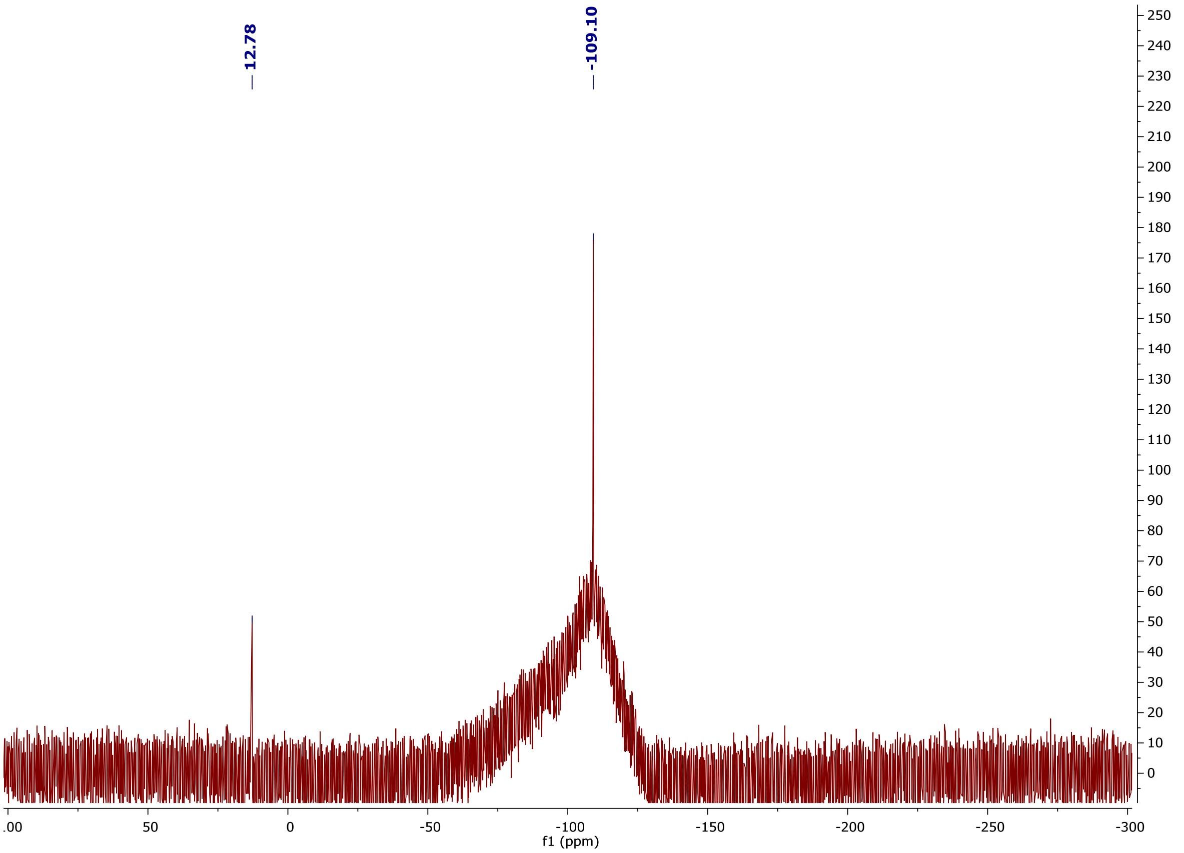

1,3,5,7,9,11,13-heptaisobutyltricyclo[5.5.1.1^{3,9}.1^{7,13}]heptasiloxane-1,5,13-triol (5)

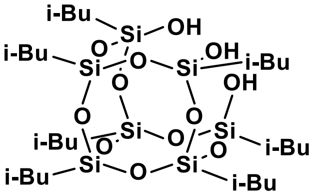

^{1}H NMR (400 MHz, $CDCl_3$): δ (ppm) = 6.83 (s, 3H, -OH), 1.92-1.77 (m, 7H, (-$CH_2C\underline{H}(CH_3)_2$), 0.97-0.94 (m, 42H, -$CH_2CH(C\underline{H_3})_2$), 0.61-0.56 (m, 14H, -$C\underline{H_2}CH(CH_3)_2$);

^{13}C NMR (101 MHz, $CDCl_3$): δ (ppm) = 25.94, 25.90, 25.80, 24.10, 24.07, 23.36, 22.98, 22.63;

^{29}Si NMR (79,5 MHz, $CDCl_3$): δ (ppm) = -58.82, -67.46, -68.66.

FT-IR (ATR) = 2953, 2928, 2906, 2871, 1464, 1398, 1383, 1366, 1332, 1228, 1168, 1085, 955, 838, 804, 746, 683, 556.

***^{1}H NMR** ($CDCl_3$, 400MHz):*

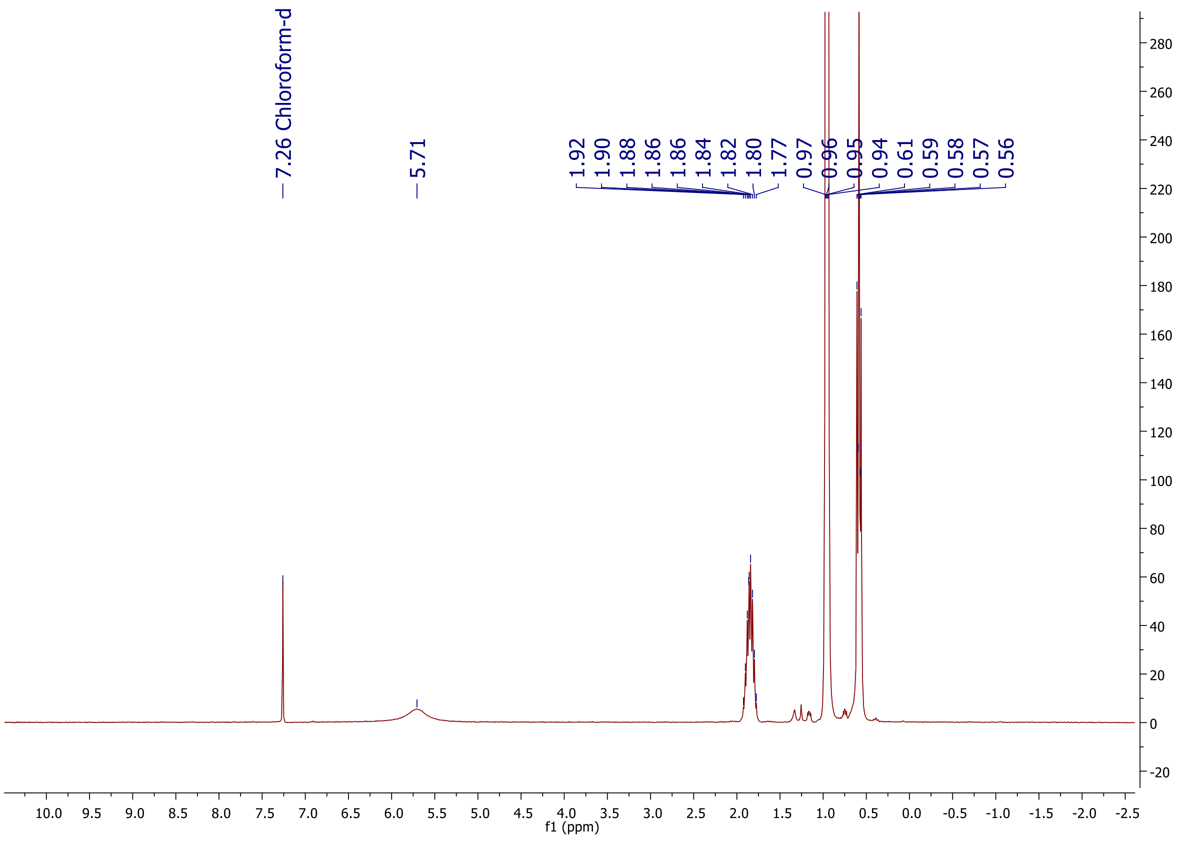

***13C NMR** (CDCl3, 101 MHz):*

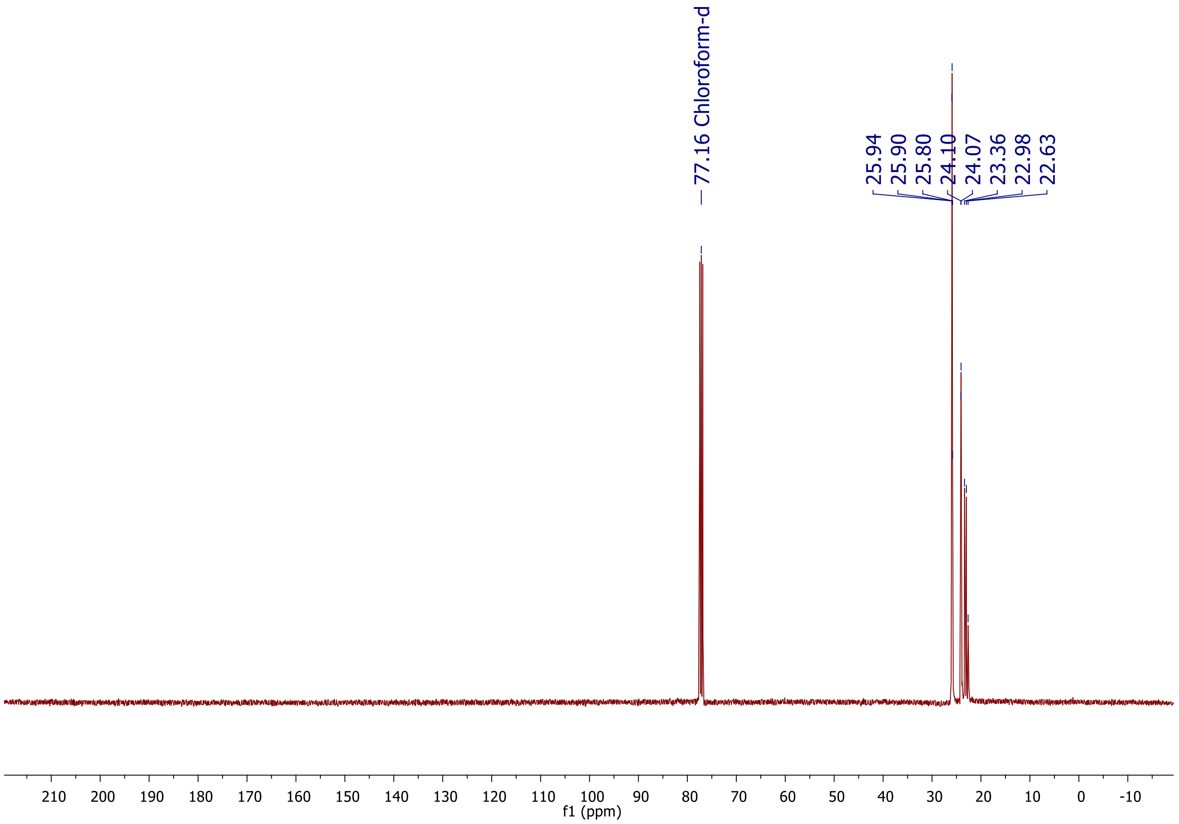

***29Si NMR** (CDCl3, 79.5 MHz):*

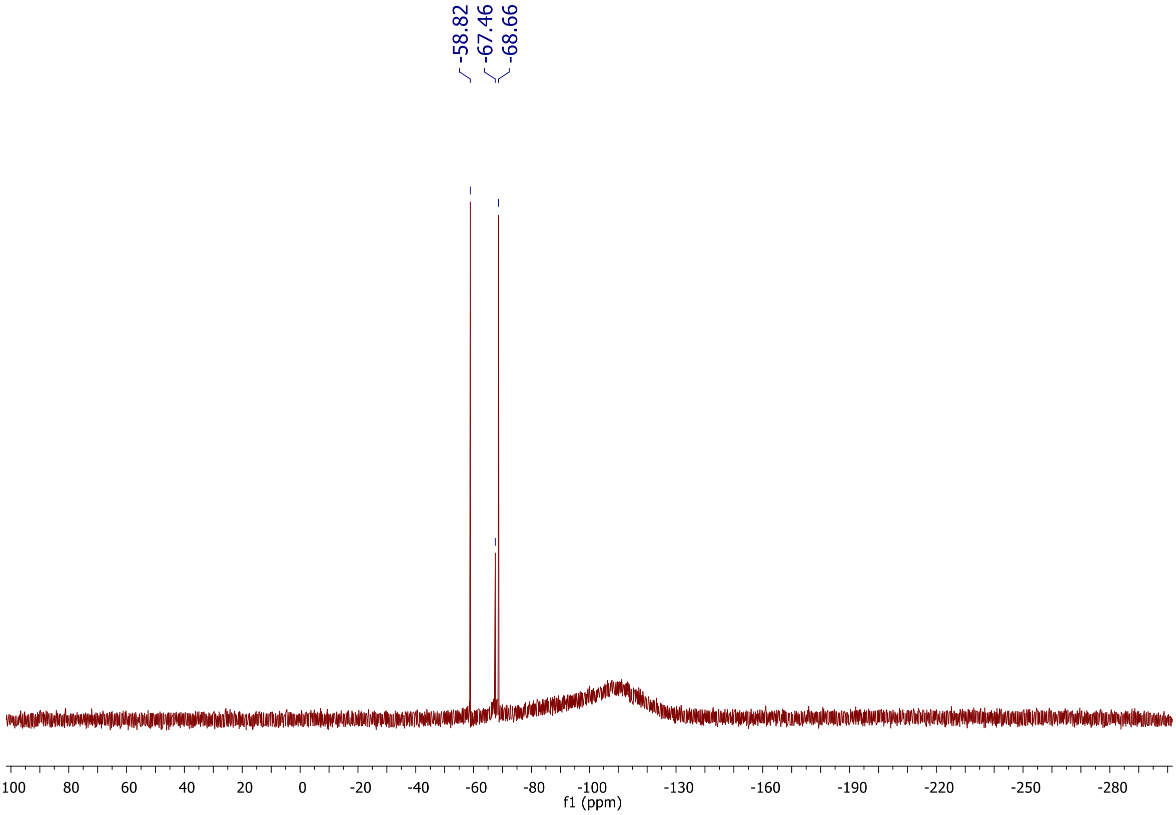

1-(3-chloropropyl)-3,5,7,9,11,13,15-heptaisobutylpentacyclo[9.5.1.1^{3,9}.1^{5,15}.1^{7,13}]octasiloxane (6)

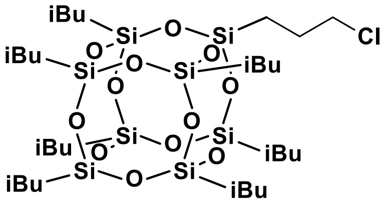

^{1}H NMR (400 MHz, $CDCl_3$): δ (ppm) = 3.52 (t, J = 8.1Hz, 2H, $-CH_2CH_2C\underline{H_2}Cl$), 1.92-1.79 (m, 9H, $-CH_2C\underline{H}(CH_3)_2$, $-CH_2C\underline{H_2}CH_2Cl$), 0.96 (d, J = 6.6Hz, 42H, $-CH_2CH(C\underline{H_3})_2$), 0.74 (t, J = 8.1Hz, 2H, $-C\underline{H_2}CH_2CH_2Cl$), 0.62-0.59 (m, 14H, $-C\underline{H_2}CH(CH_3)_2$);
^{13}C NMR (101 MHz, $CDCl_3$): δ (ppm) = 47.41 ($-CH_2CH_2\underline{CH_2}Cl$), 26.63 $-CH_2\underline{CH_2}CH_2Cl$), 25.85, 24.03, 22.65, 22.59 (iBu), 9.94 ($-C\underline{H_2}CH_2CH_2Cl$);
^{29}Si NMR (79,5 MHz, $CDCl_3$): δ (ppm) = -67.59, -67.87, -68.12.
FT-IR (ATR) = 2953, 2928, 2905, 2871, 1464, 1398, 1383, 1366, 1332, 1229, 1168, 1081, 955, 916, 838, 804, 745, 680, 556.

^{1}H NMR ($CDCl_3$, 400MHz):

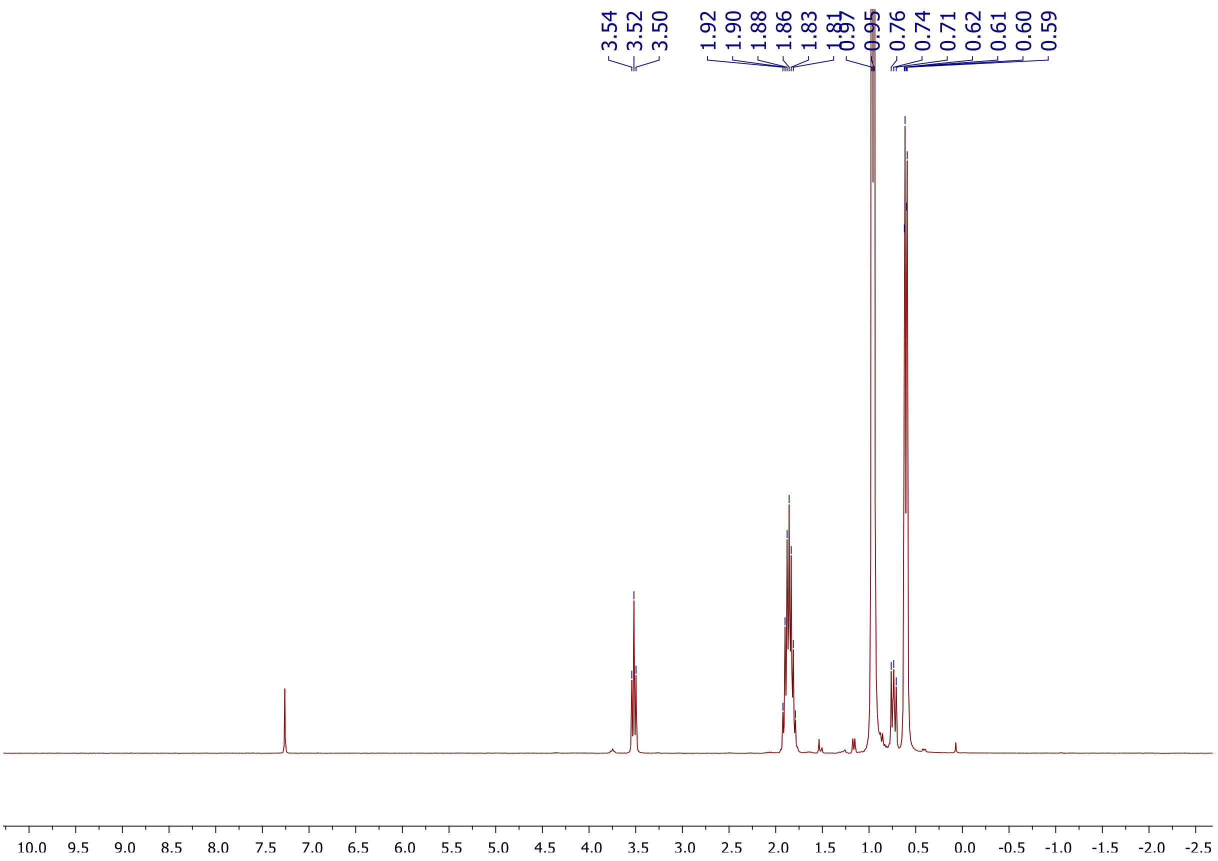

^{13}C NMR *($CDCl_3$, 101 MHz):*

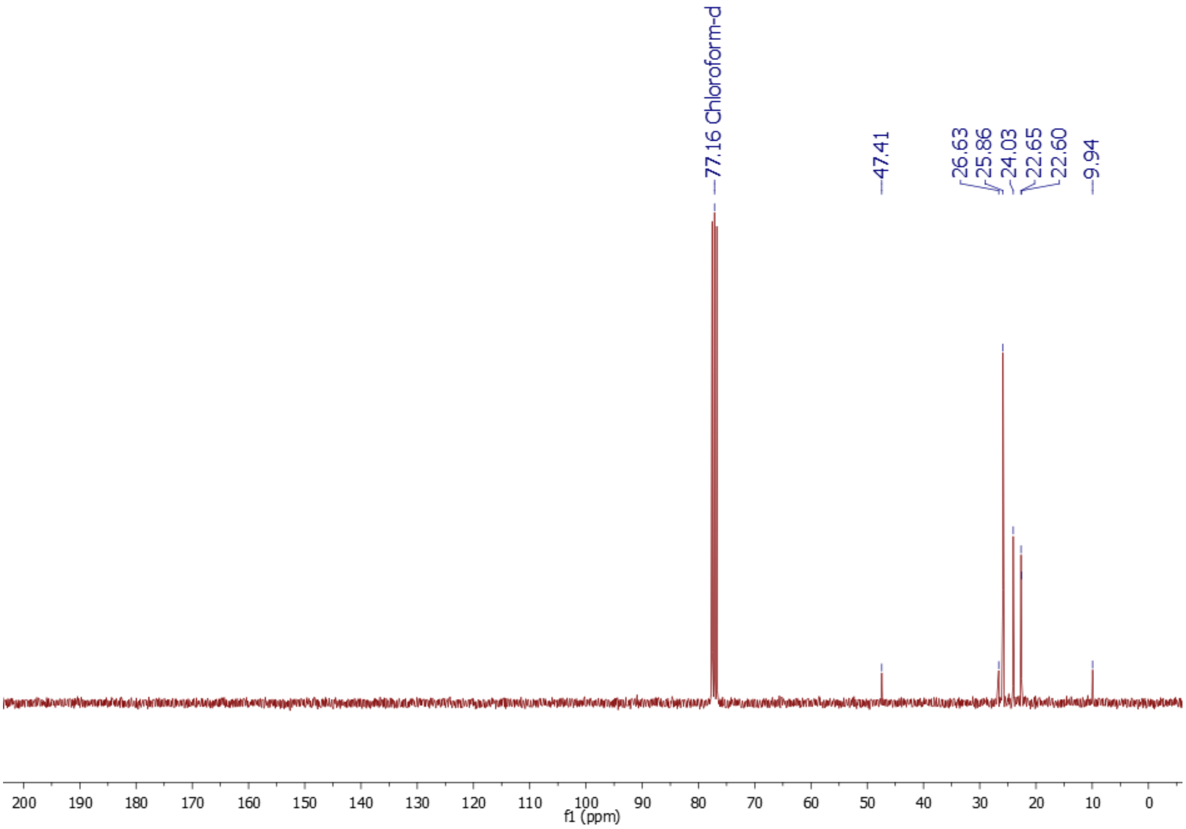

^{29}Si NMR *($CDCl_3$, 79.5 MHz):*

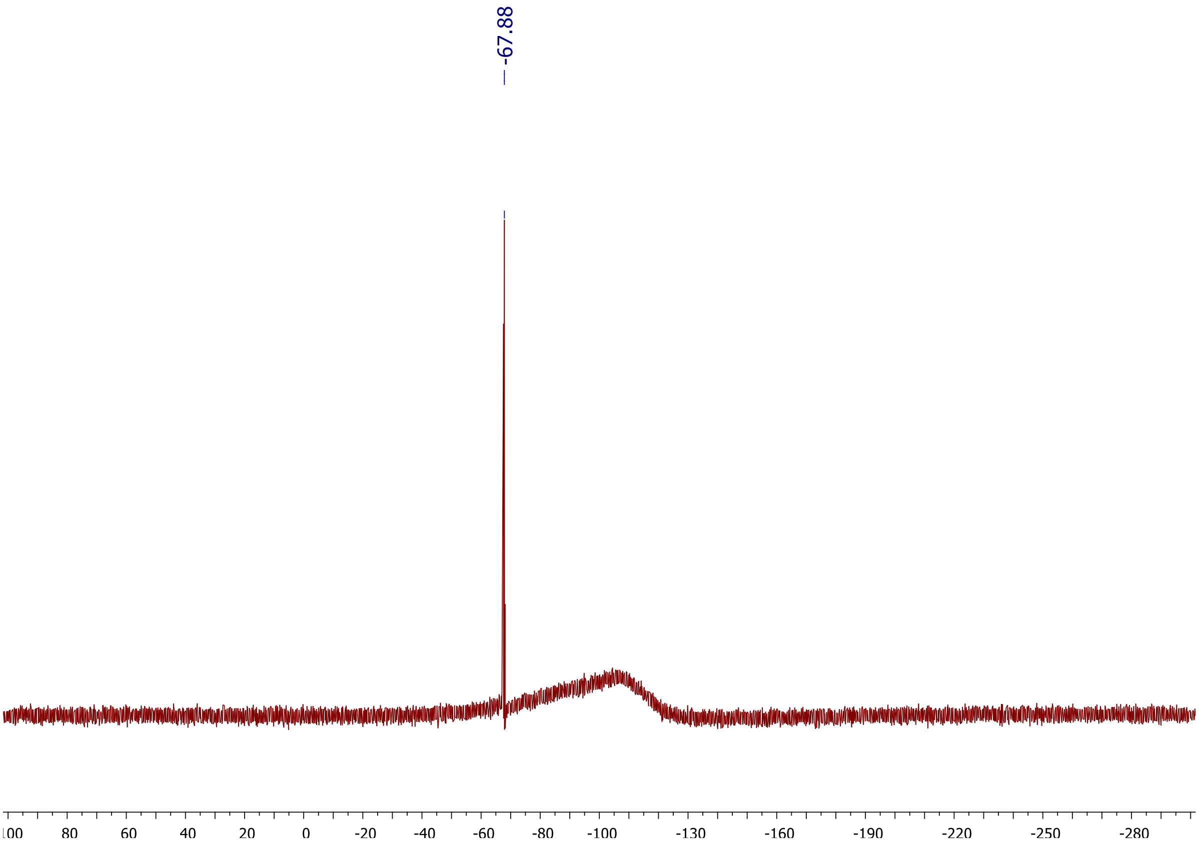

1-(dimethylvinylsiloxy)-3,5,7,9,11,13,15-heptaisobutylpentacyclo[$9.5.1.1^{3,9}.1^{5,15}.1^{7,13}$]octasiloxane (7)

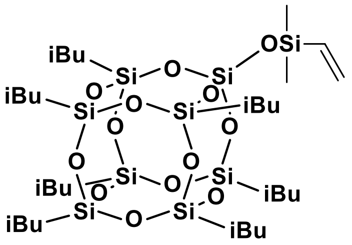

^{1}H NMR (400 MHz, $CDCl_3$): δ (ppm) = 6.12 (dd, J1 = 20.0Hz, J2 = 14.9Hz, 1H, $-C\underline{H}=CH_aH_b$), 5.95 (dd, J1 = 14.9 Hz, J2 = 4.3Hz, 1H, $-CH=C\underline{H_a}H_b$), 5.77 (dd, J1 = 20.0Hz, J2 = 4.3Hz, 1H, $-CH=CH_a\underline{H_b}$), 1.93-1.79 (m, 7H, $-CH_2C\underline{H}(CH_3)_2$), 0.96 (d, J = 6.6Hz, 42H. $-CH_2CH(C\underline{H_3})_2$), 0.62-0.59 (m, 14H, $-C\underline{H_2}CH(CH_3)_2$), 0.19 (s, 6H, $SiMe_2$);
^{13}C NMR (101 MHz, $CDCl_3$): δ (ppm) = 138.50, 132.25 (Vi), 25.85, 25.82, 24.02, 23.96, 22.62, 22.57, 22.54, 22.45 (iBu), -0.03 ($SiMe_2$);
^{29}Si NMR (79,5 MHz, $CDCl_3$): δ (ppm) = -0.71 ($SiMe_2$), -67.01, -67.87, -67.89 (cage), -109.59 (SiO_4).
FT-IR (ATR) = 2953, 2926, 1905, 2869, 1465, 1401, 1383, 1366, 1332, 1253, 1229, 1208, 1074, 954, 901, 837, 787, 769, 738, 626, 561.

^{1}H NMR ($CDCl_3$, 400MHz):

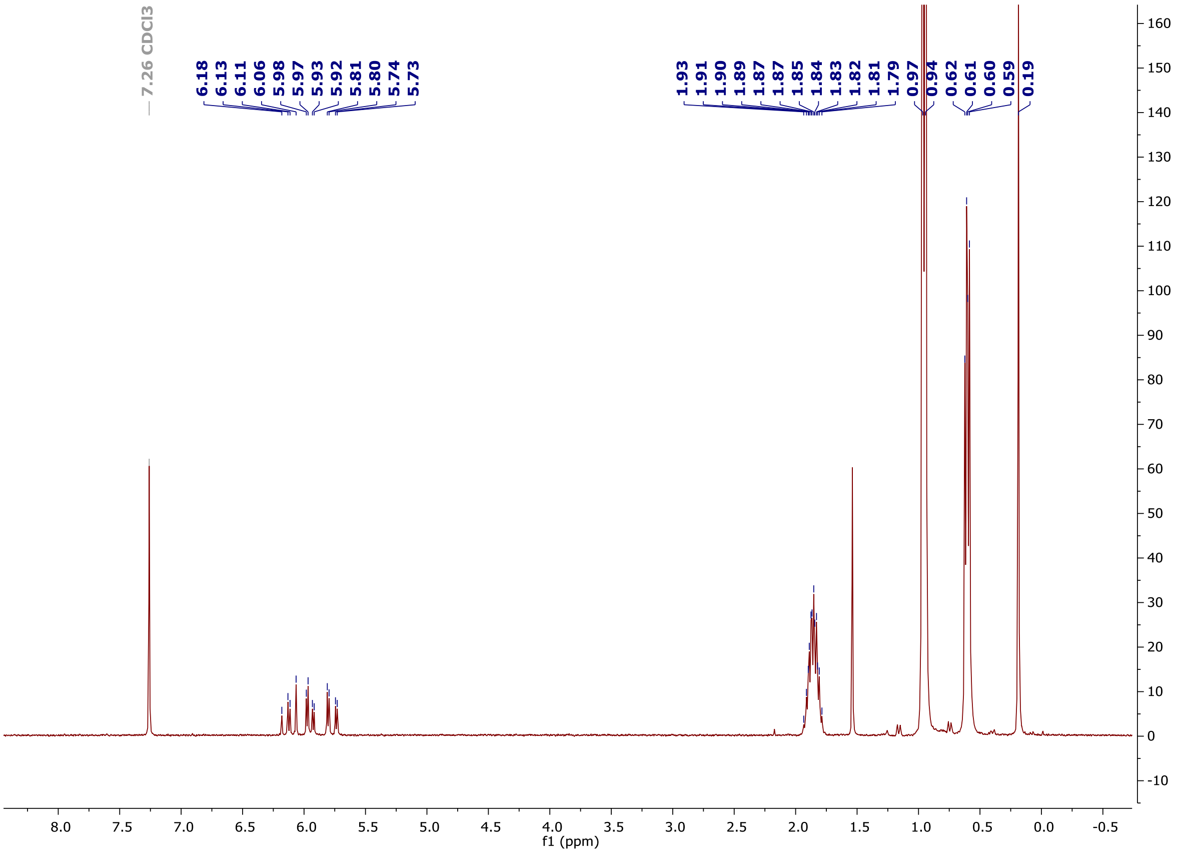

^{13}C NMR *(CDCl3, 101 MHz):*

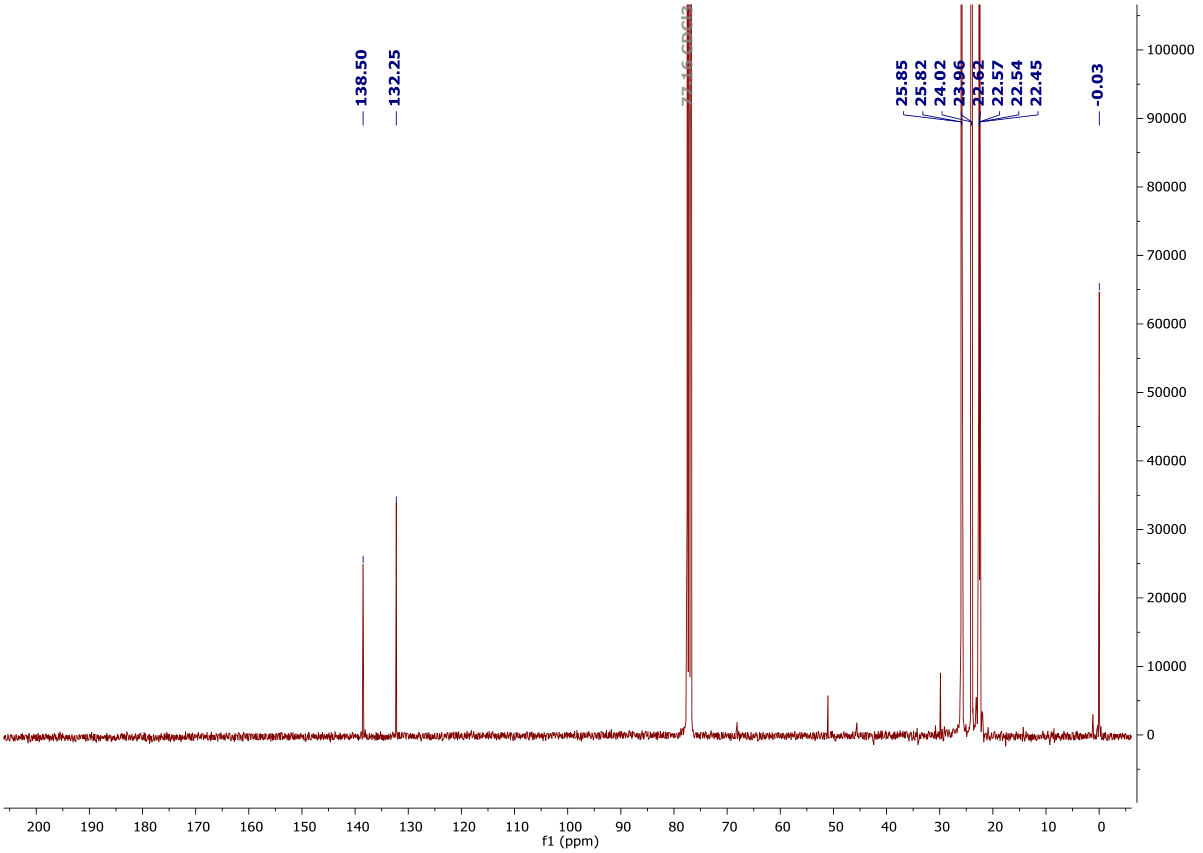

^{29}Si NMR *(CDCl3, 79.5 MHz):*

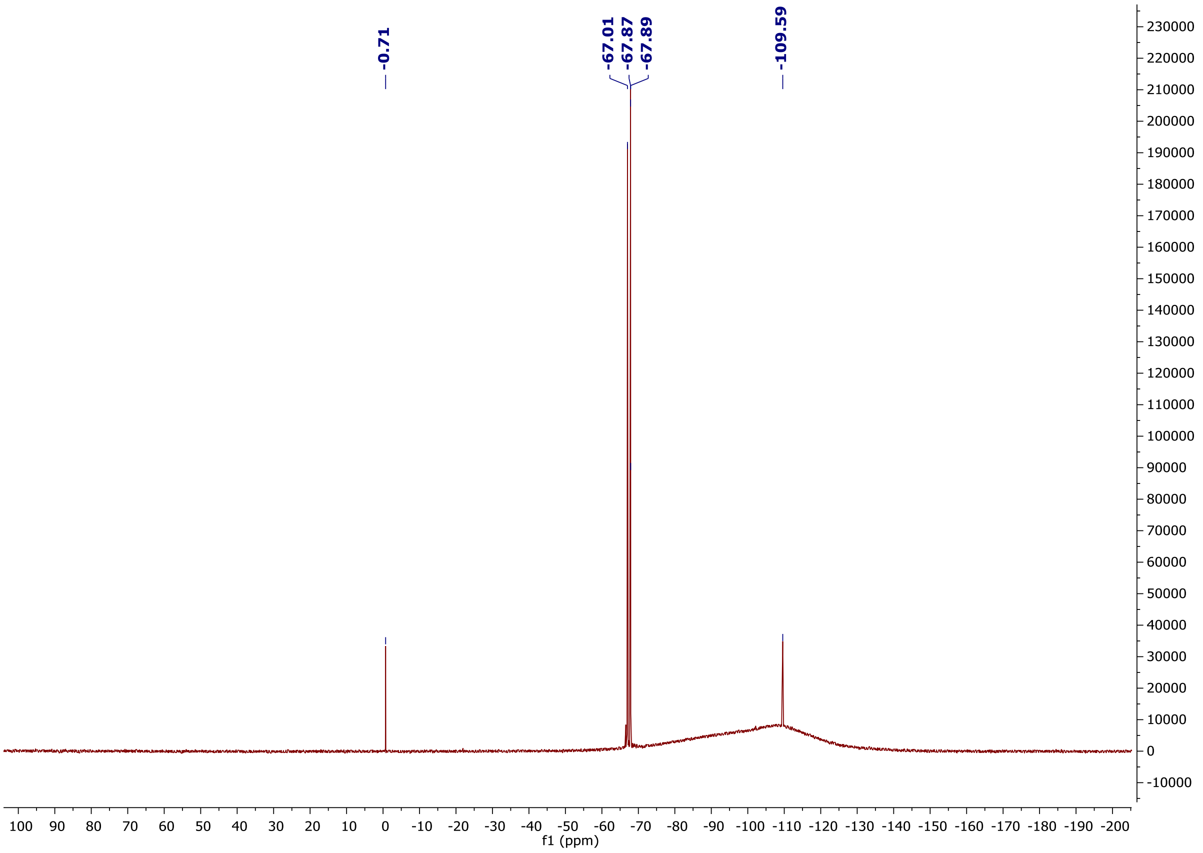

1-(dimethylsiloxy)-3,5,7,9,11,13,15-heptaisobutylpentacyclo[9.5.1.1^{3,9}.1^{5,15}.1^{7,13}]octasiloxane (8)

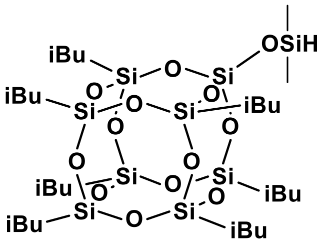

^{1}**H NMR** (400 MHz, $CDCl_3$): δ (ppm) = 4.72 (sept, J = 2.9Hz, 1H, SiH), 1.93-1.81 (m, 7H, -$CH_2C\underline{H}(CH_3)_2$), 0.98-0.95 (m, 42H. -$CH_2CH(C\underline{H_3})_2$), 0.64-0.60 (m, 14H, -$C\underline{H_2}CH(CH_3)_2$), 0.23 (d, J = 2.9Hz, 6H, $SiMe_2$);
^{13}**C NMR** (101 MHz, $CDCl_3$): δ (ppm) = 25.87, 25.85, 24.04, 24.00, 22.68, 22.63, 22.53 (iBu), 0.36 ($SiMe_2$);
^{29}**Si NMR** (79,5 MHz, $CDCl_3$): δ (ppm) = -3.00 ($SiMe_2$), -66.93, -67.86, -67.88 (cage), -109.05 (SiO_4).
FT-IR (ATR) = 2953, 2927, 2906, 2870, 2143, 1464, 1430, 1383, 1366, 1332, 1253, 1229, 1074, 955, 899, 838, 770, 740, 697, 627, 561, 529.

^{1}H NMR ($CDCl_3$, 400MHz):

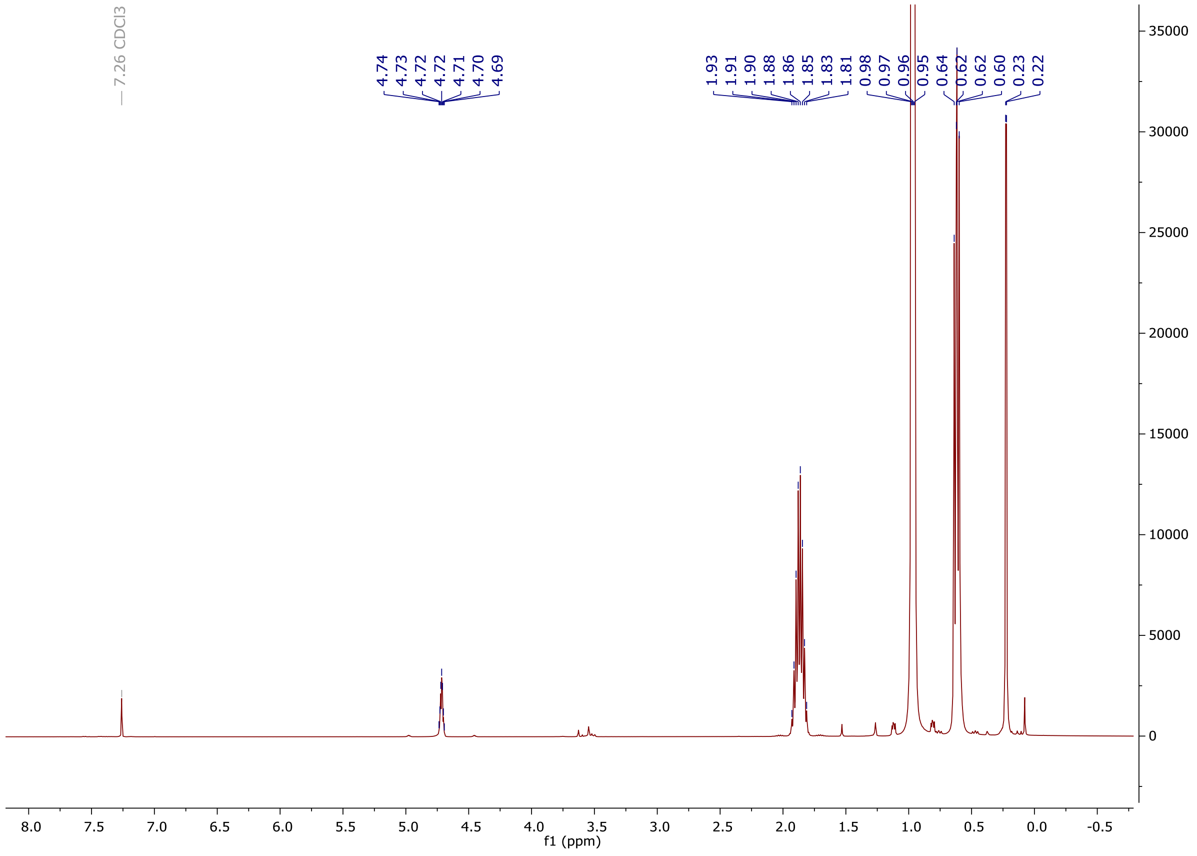

^{13}**C NMR** *(CDCl$_3$, 101 MHz):*

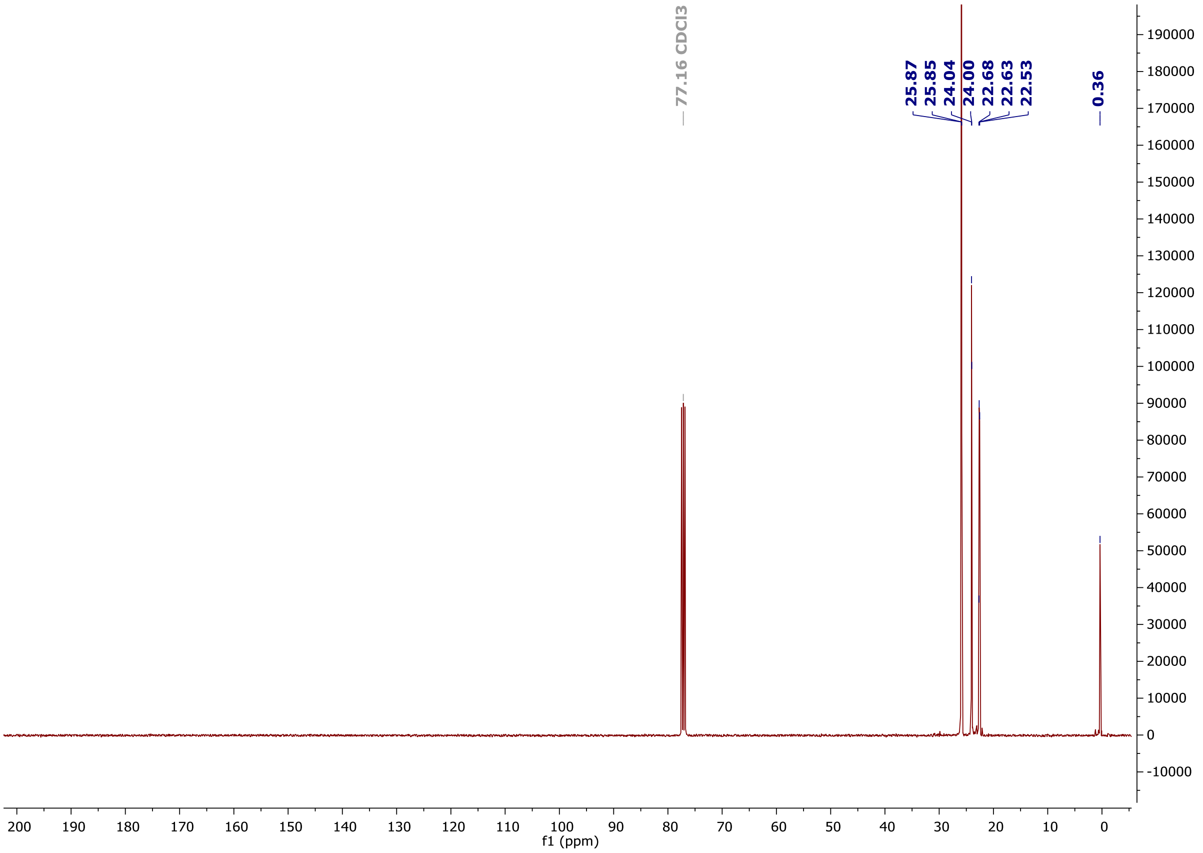

^{29}**Si NMR** *(CDCl$_3$, 79.5 MHz):*

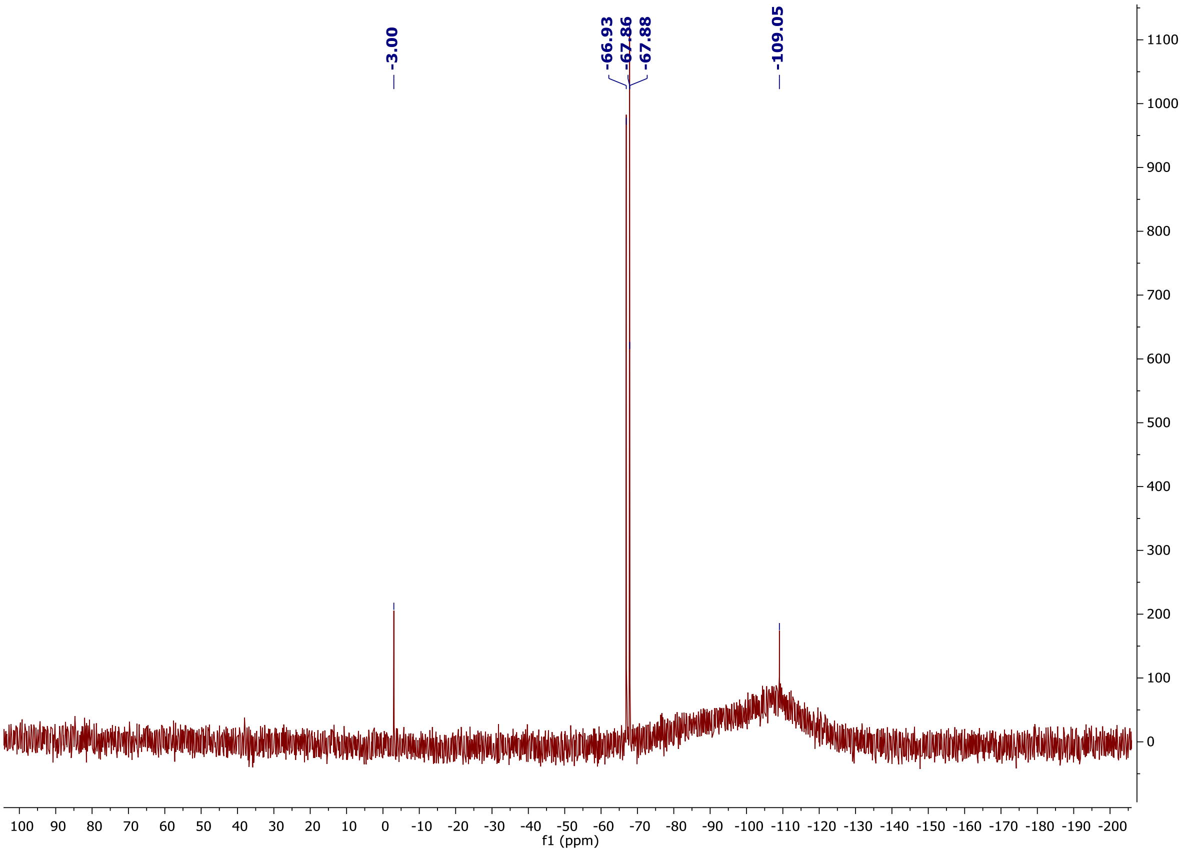

3. SEM and EDS images of the SS/PP composites

0.1% SS-Vi/PP

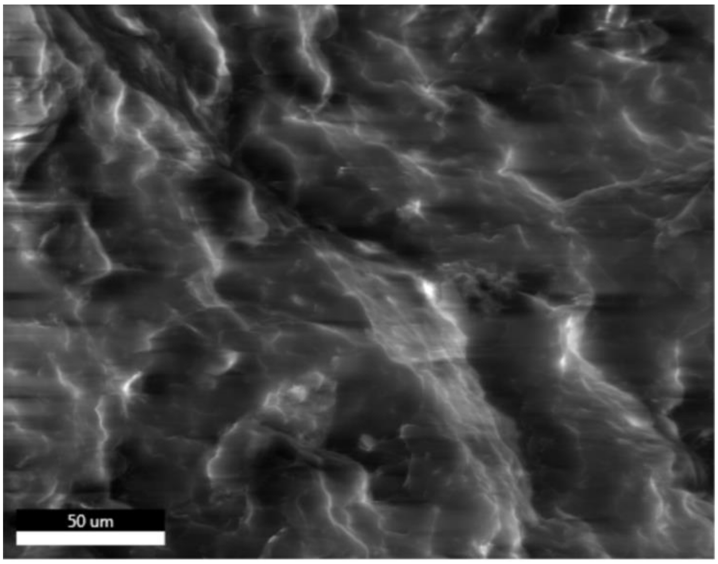

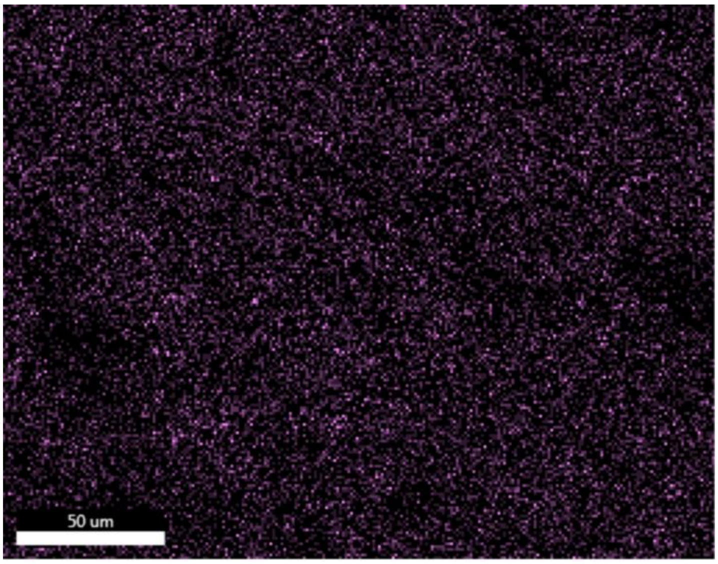

0.25% SS-Vi/PP

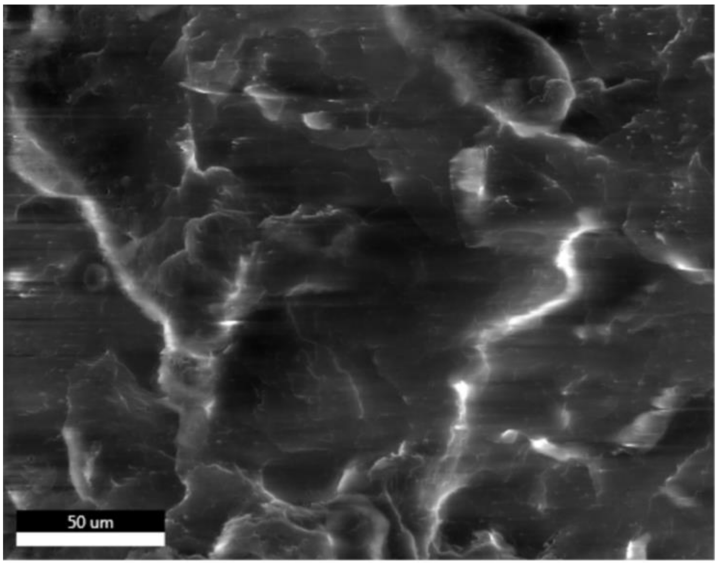

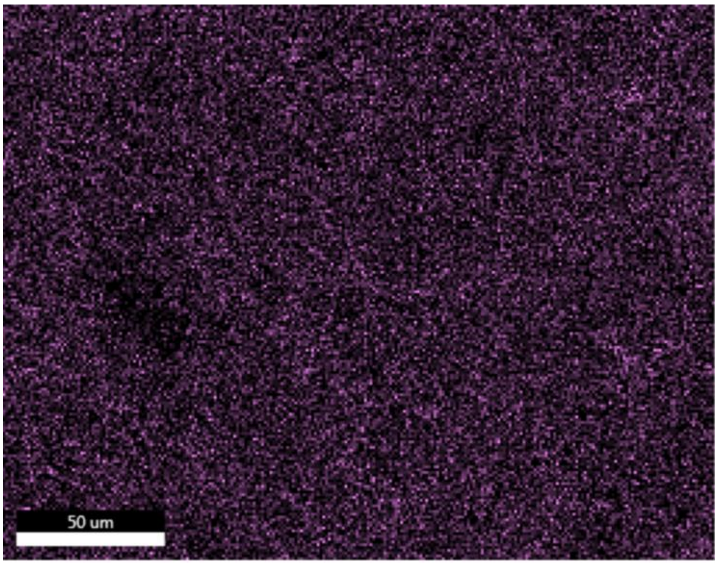

0.5% SS-Vi/PP

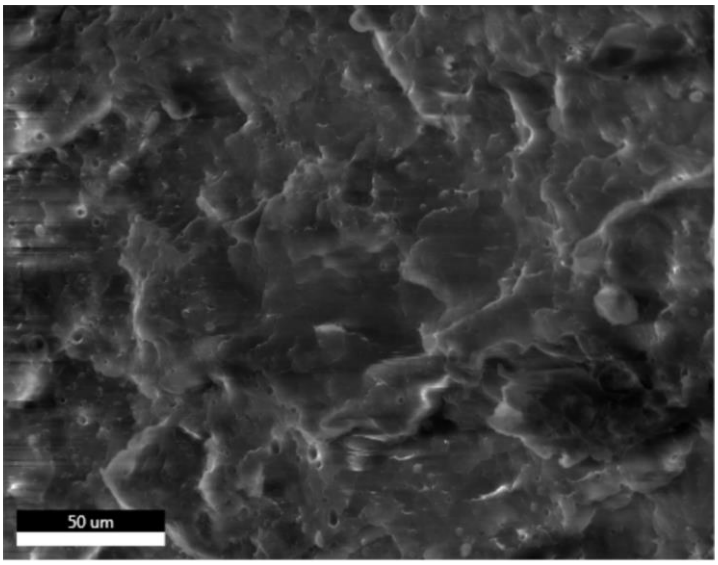

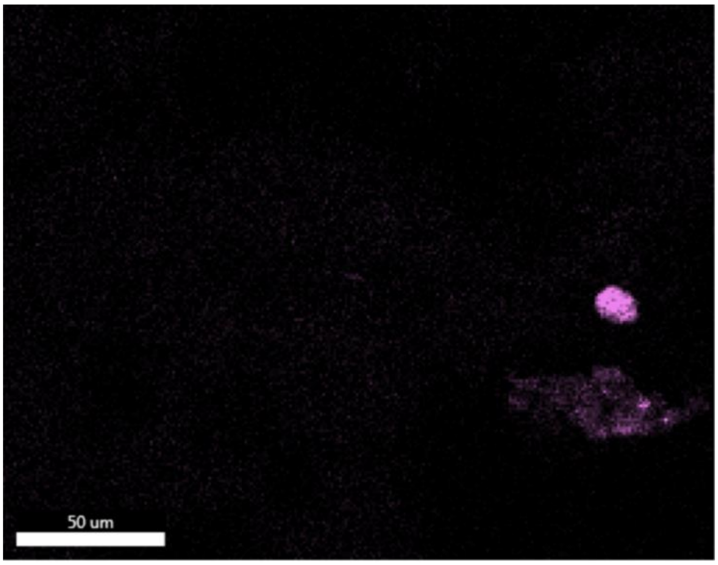

1% SS-Vi/PP

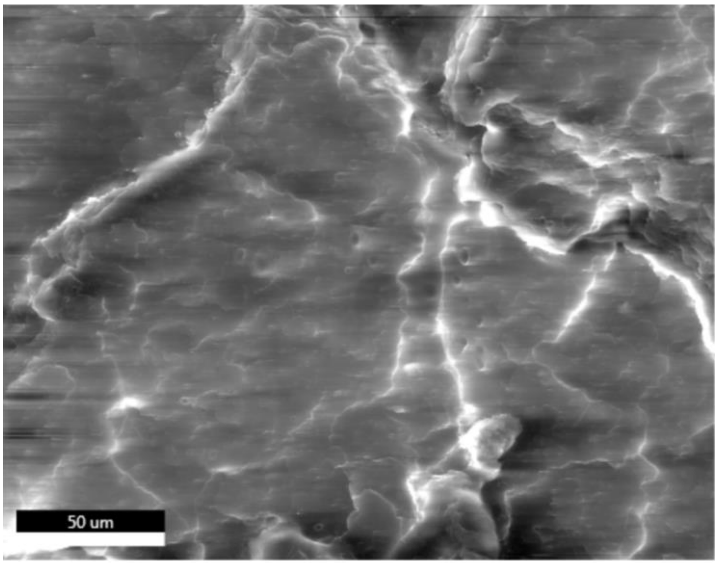

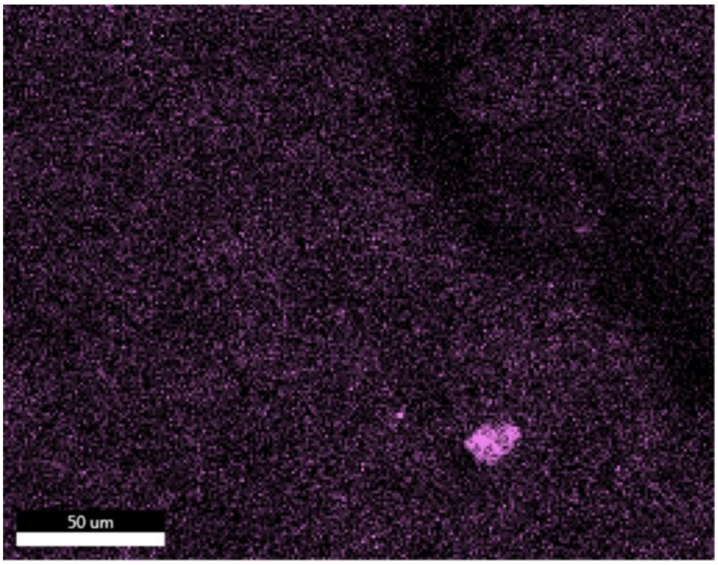

0.1% SS-Glycidyl/PP

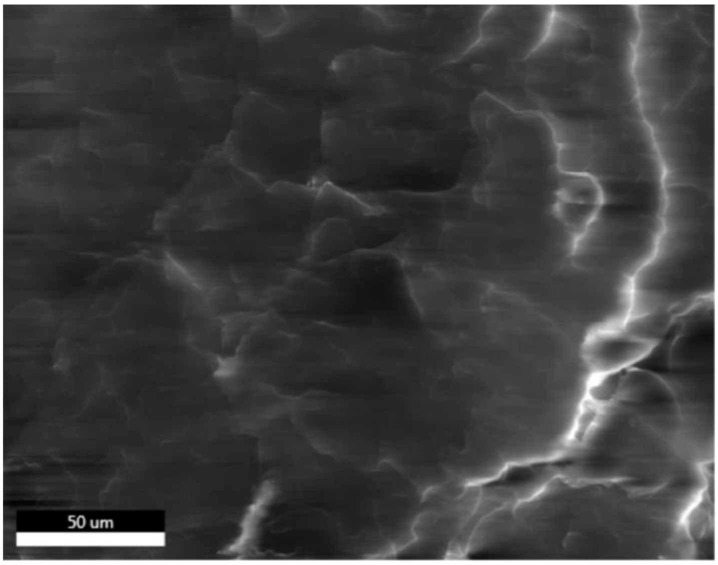

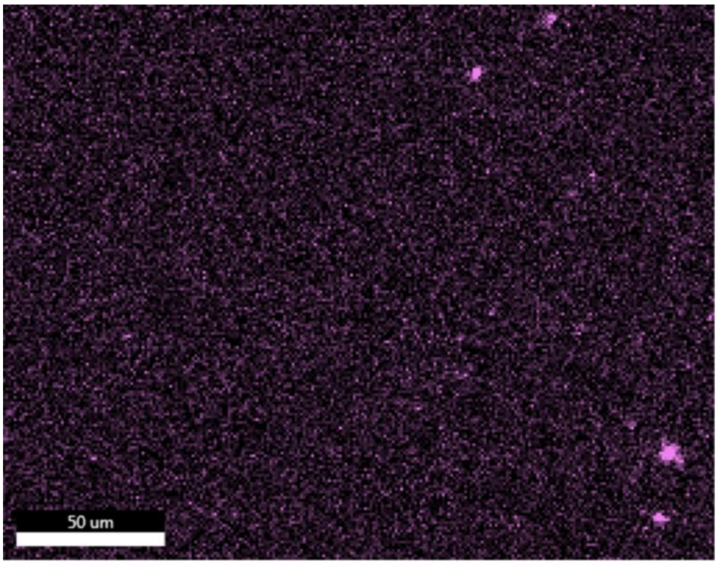

0.25% SS-Glycidyl/PP

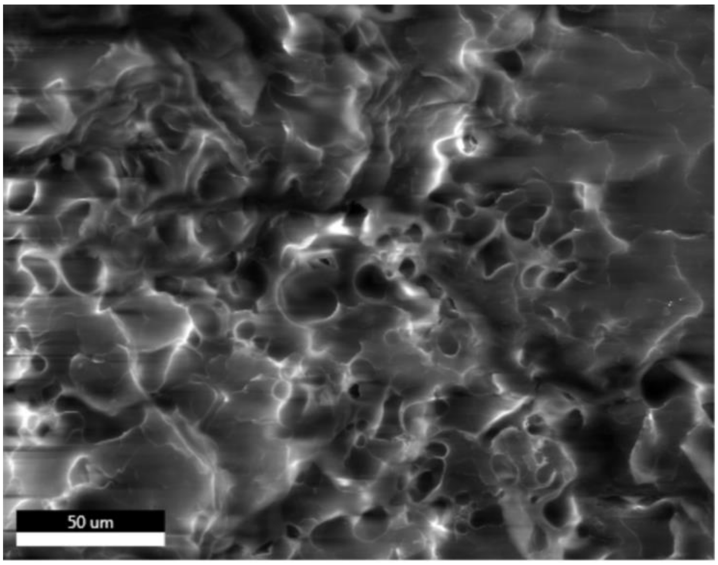

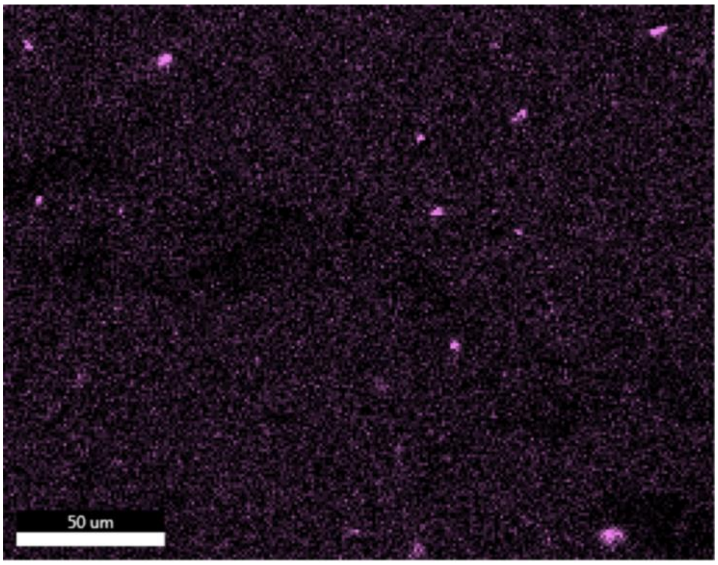

0.5% SS-Glycidyl/PP

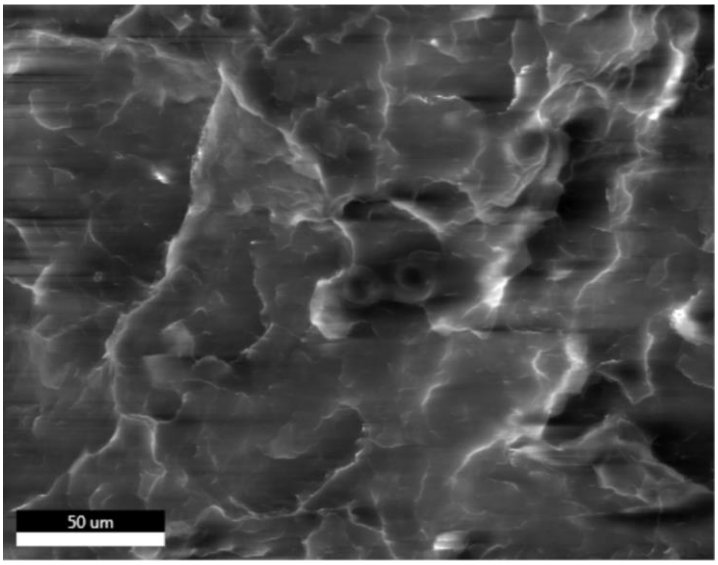

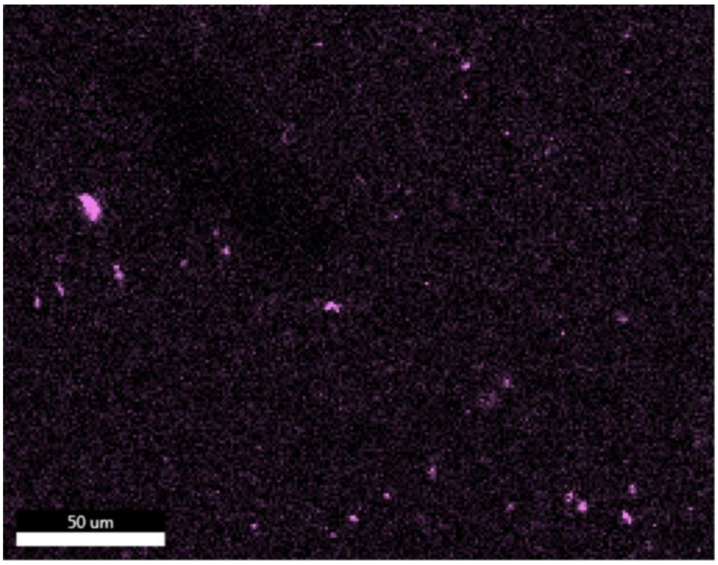

1% SS-Glycidyl/PP

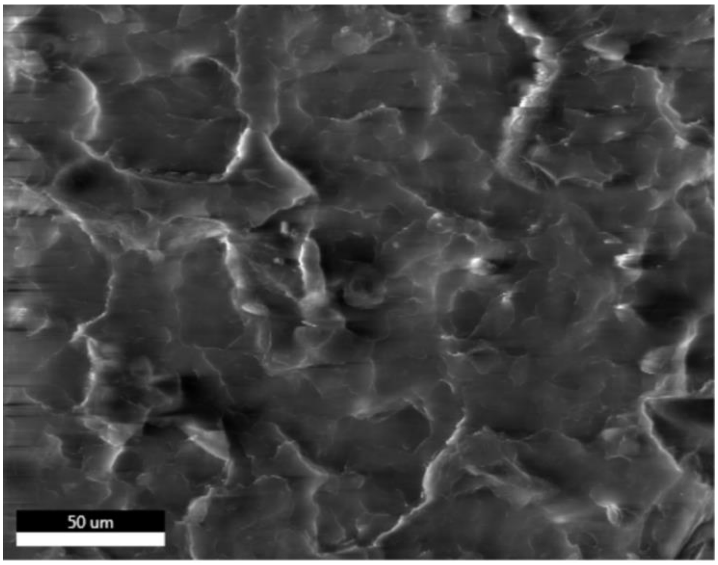

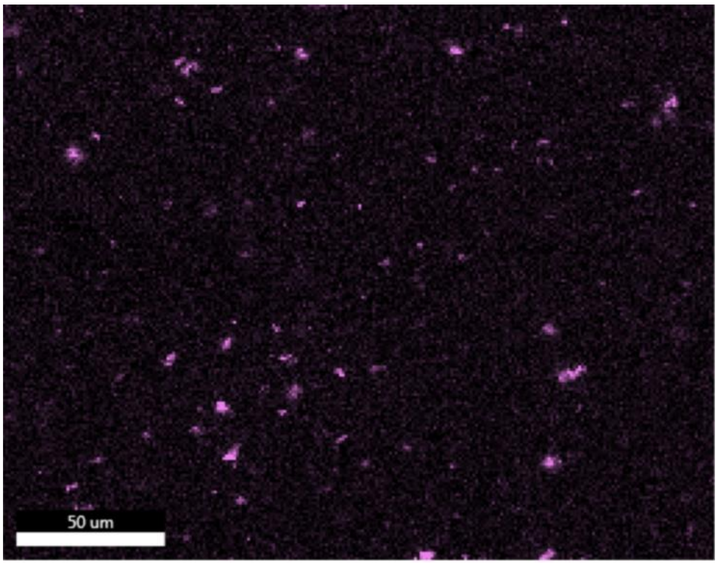

0.1% SS-Limonene/PP

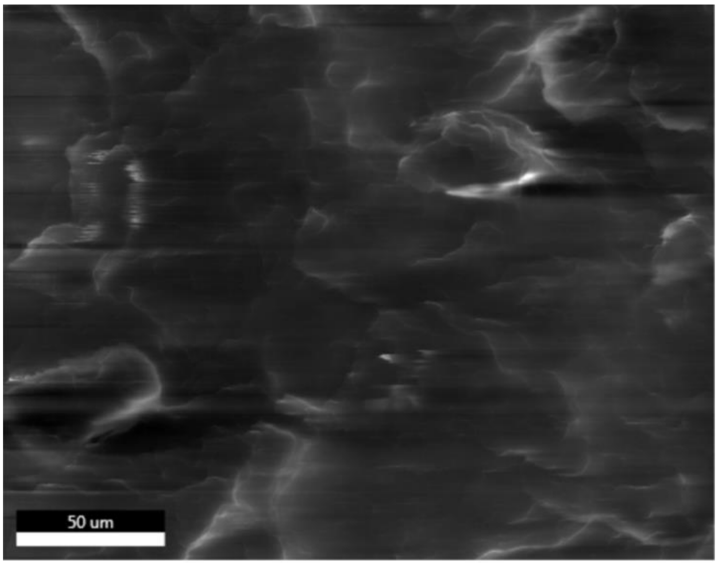

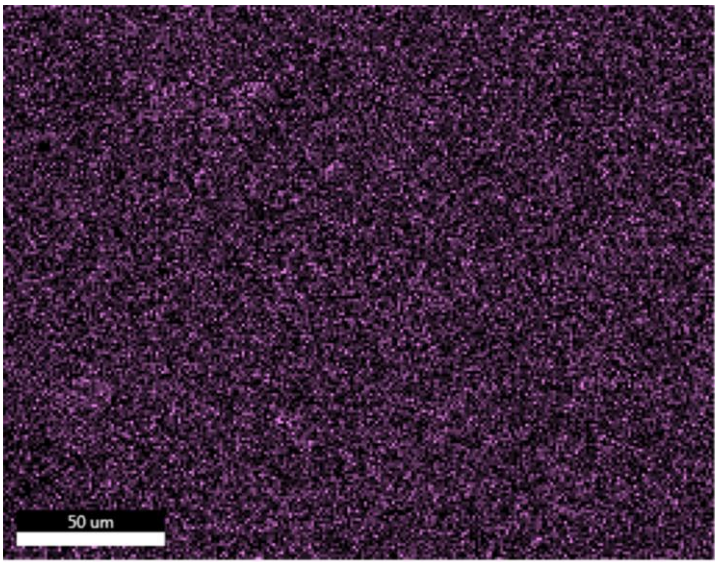

0.25% SS-Limonene/PP

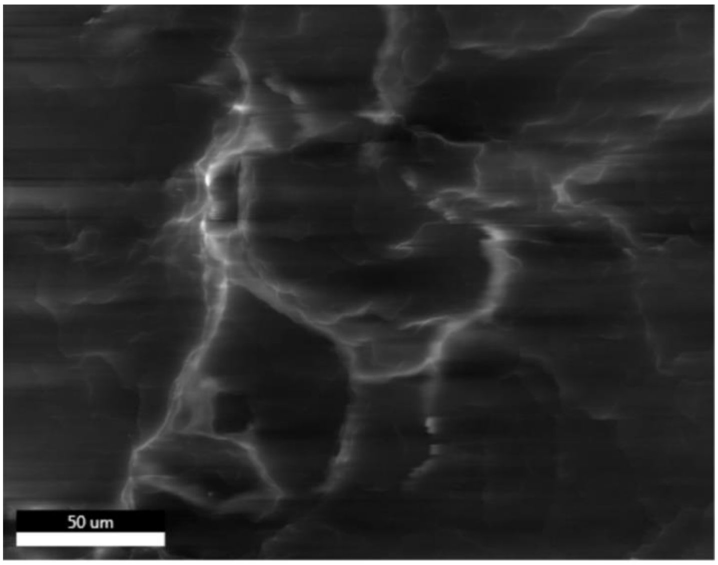

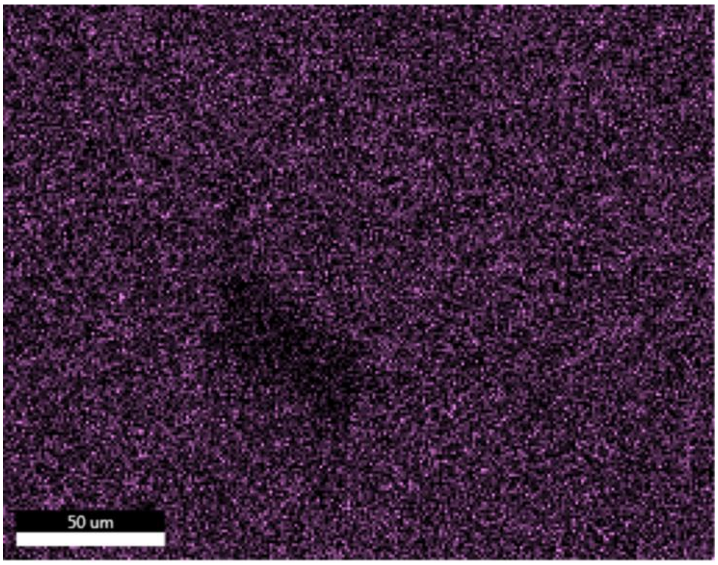

0.5% SS-Limonene/PP

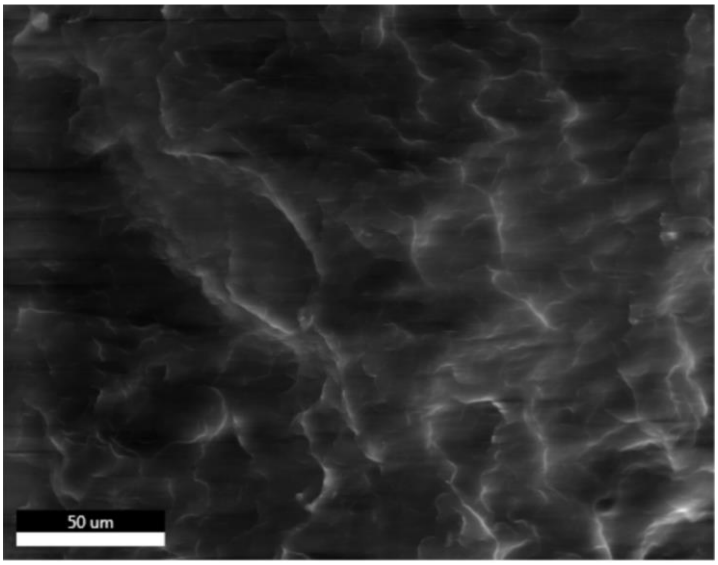

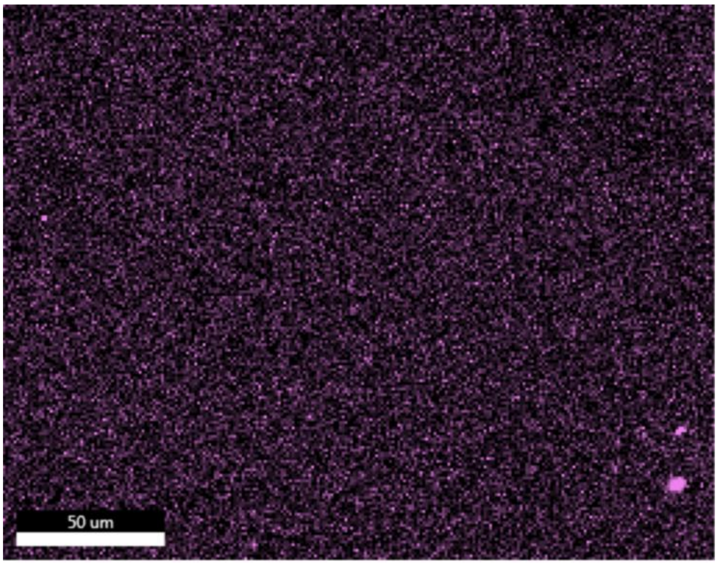

1% SS-Limonene/PP

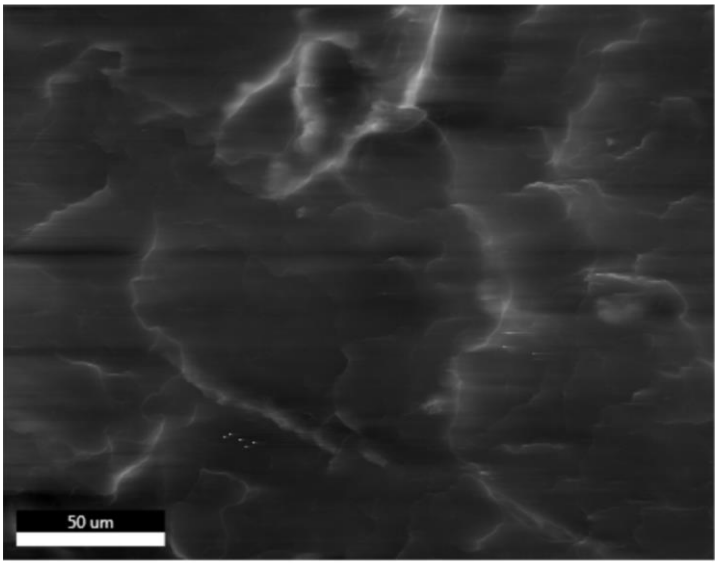

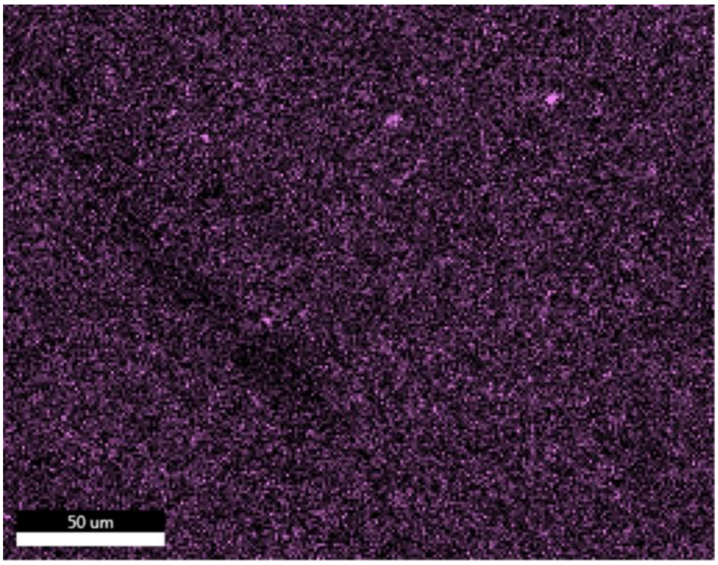

0.1% iBu_7SSQ-3OH/PP

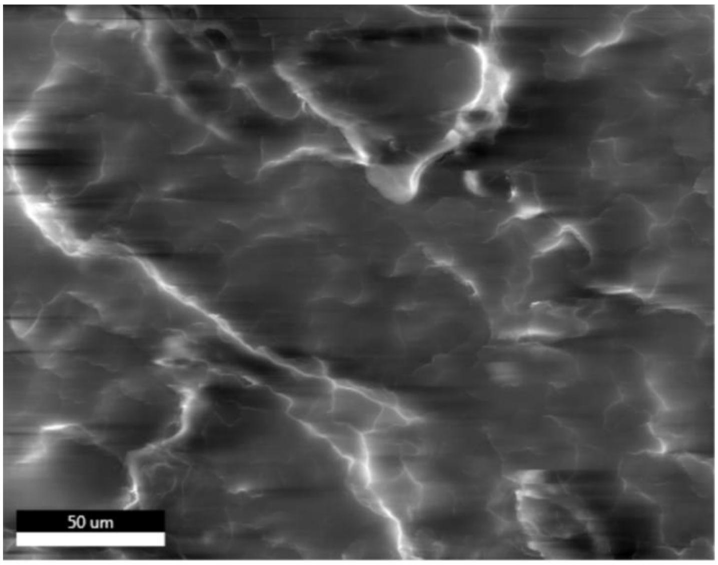

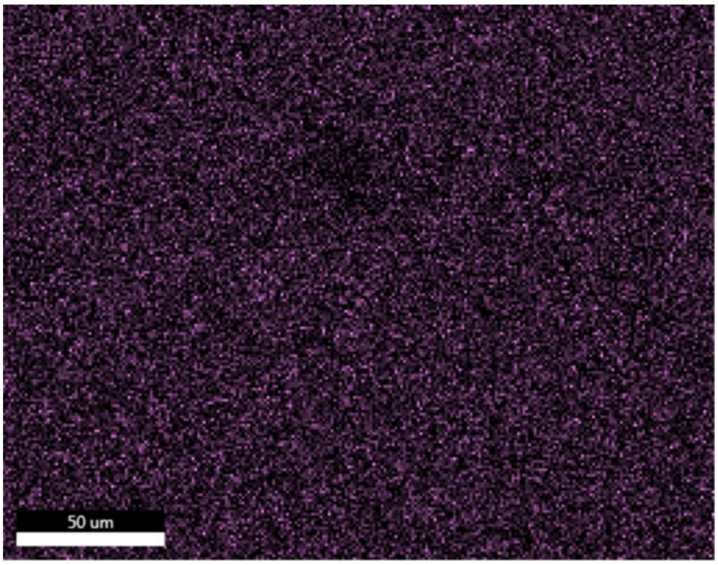

0.25% iBu_7SSQ-3OH/PP

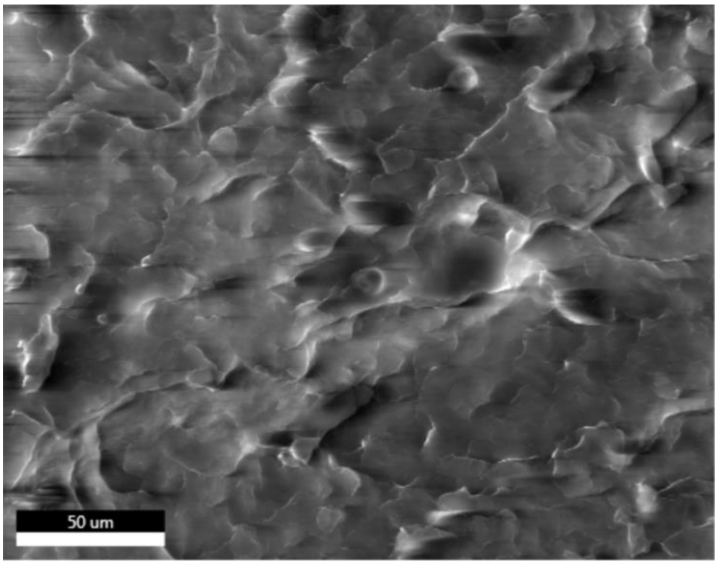

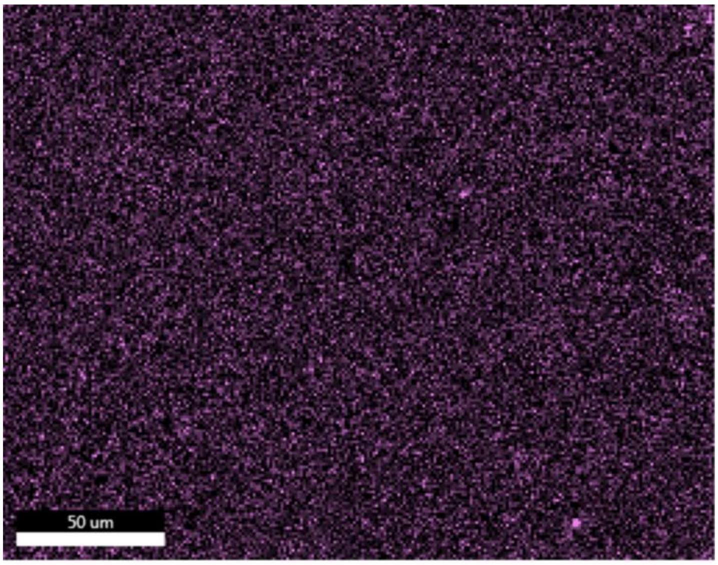

0.5% iBu$_7$SSQ-3OH/PP

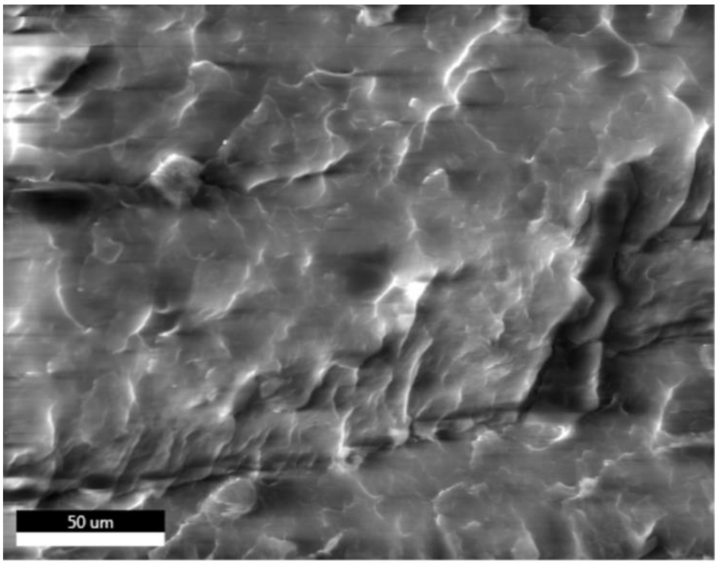

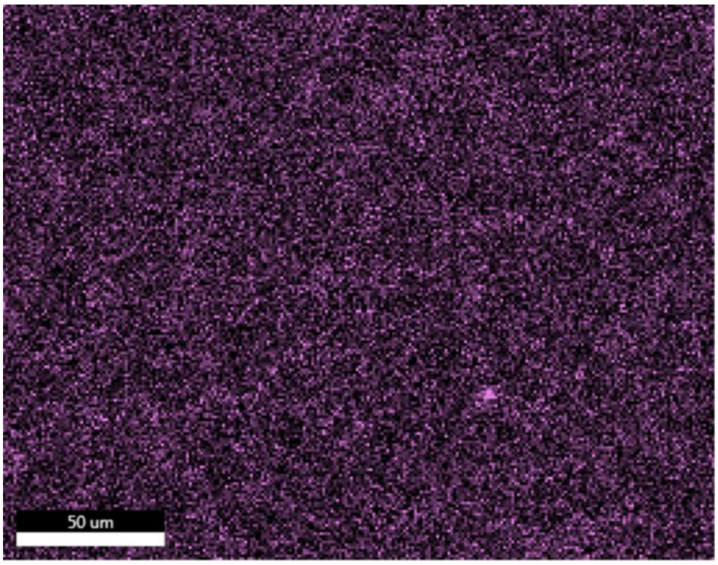

1% iBu_7SSQ-3OH/PP

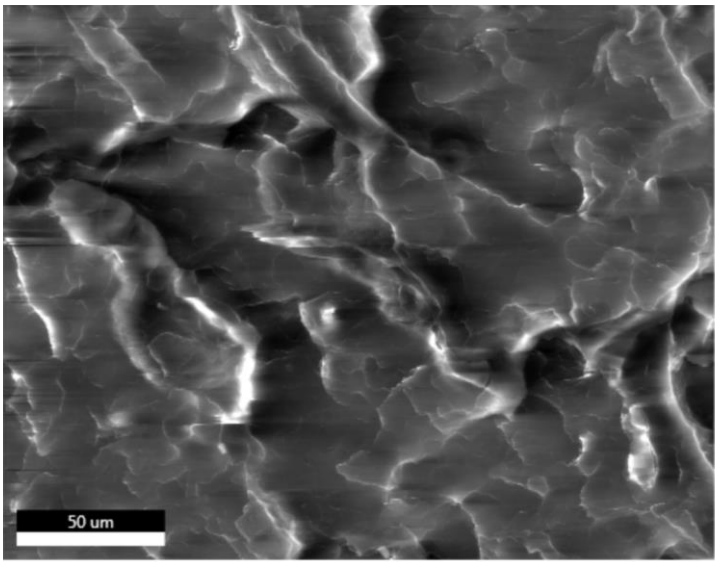

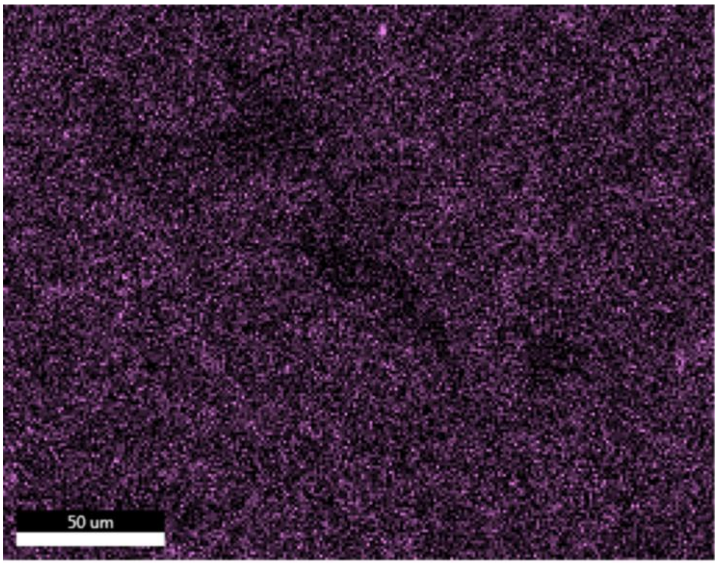

0.1% iBu_7SSQ-Cl/PP

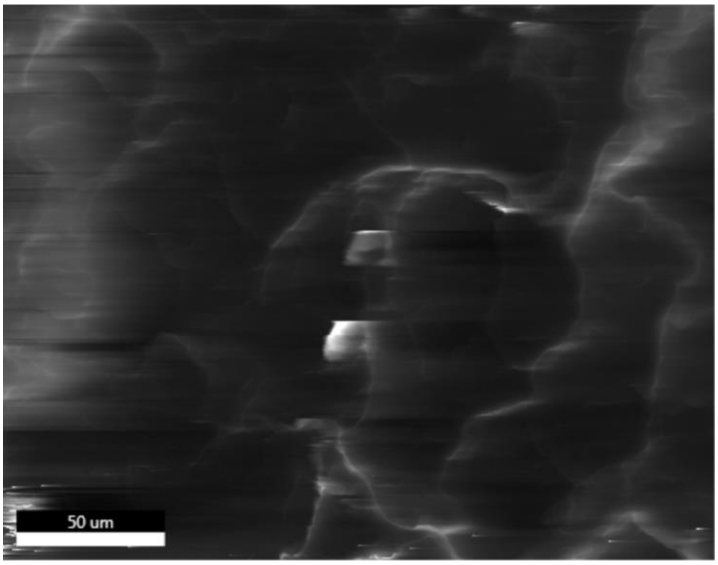

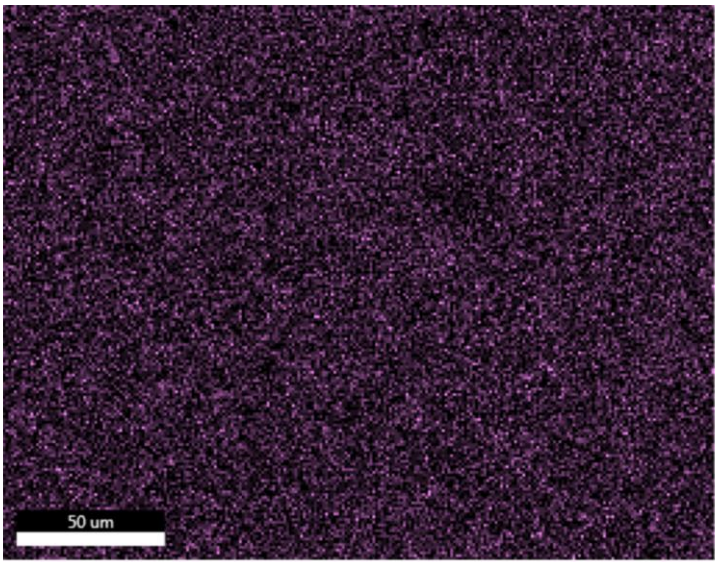

0.25% iBu_7SSQ-Cl/PP

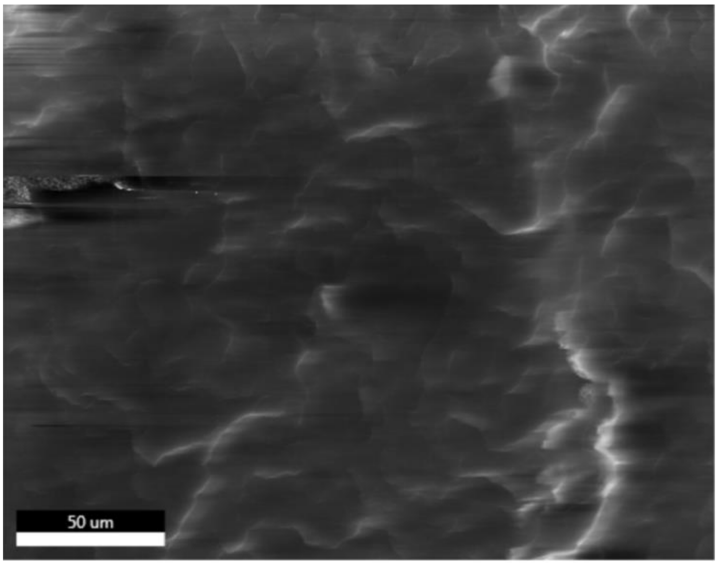

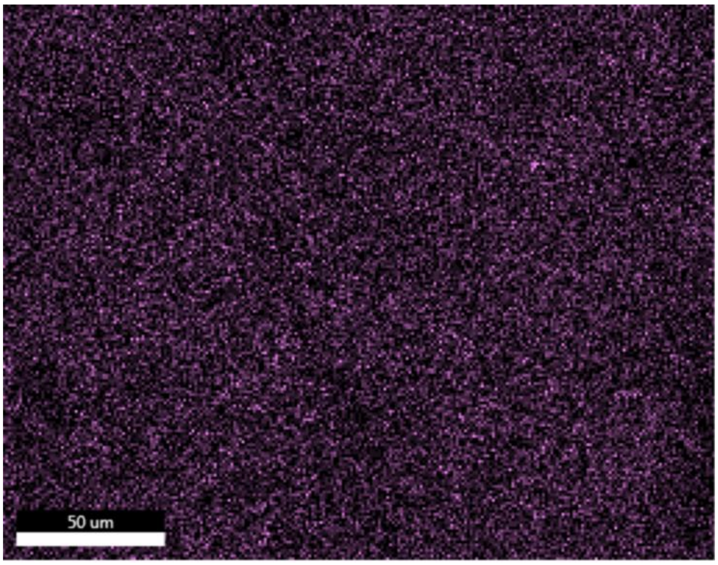

0.5% iBu_7SSQ-Cl/PP

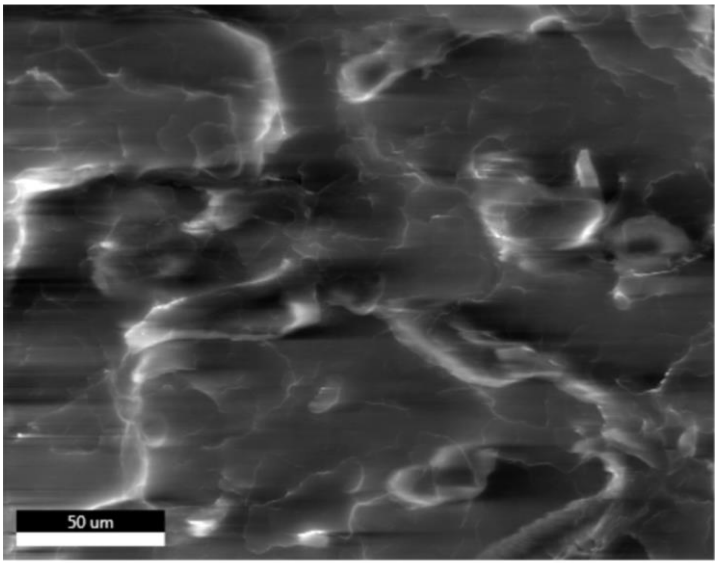

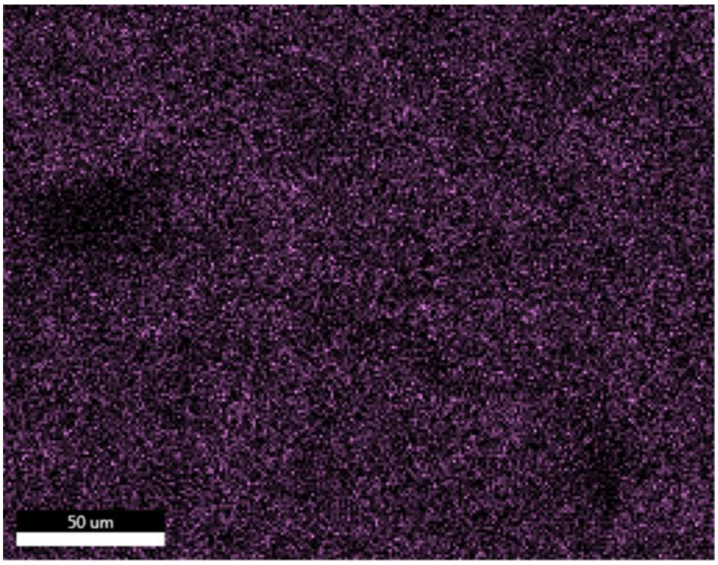

1% iBu_7SSQ-Cl/PP

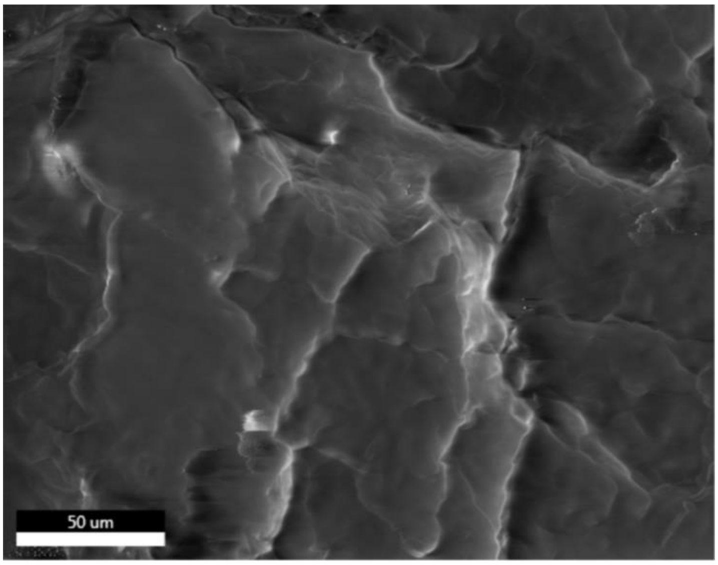

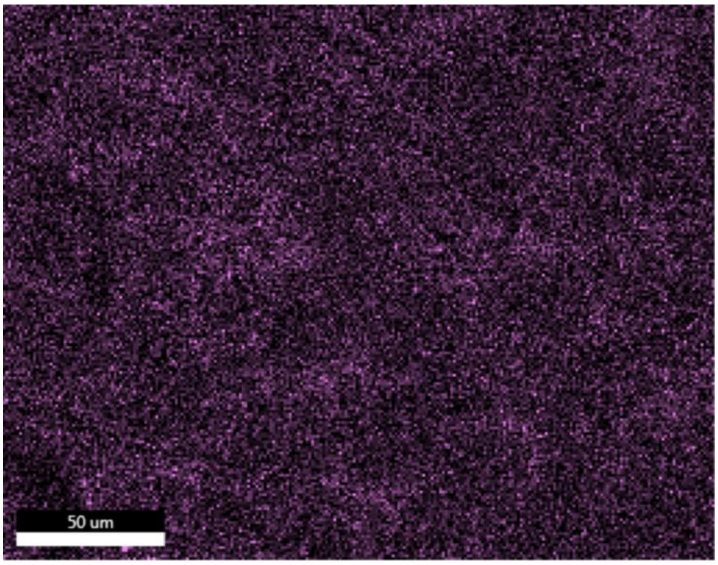

0.1% iBu_7SS-Vi/PP

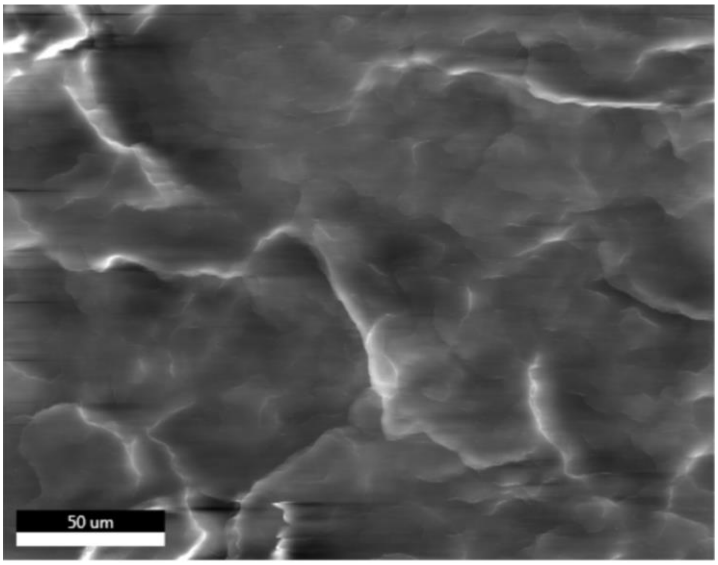

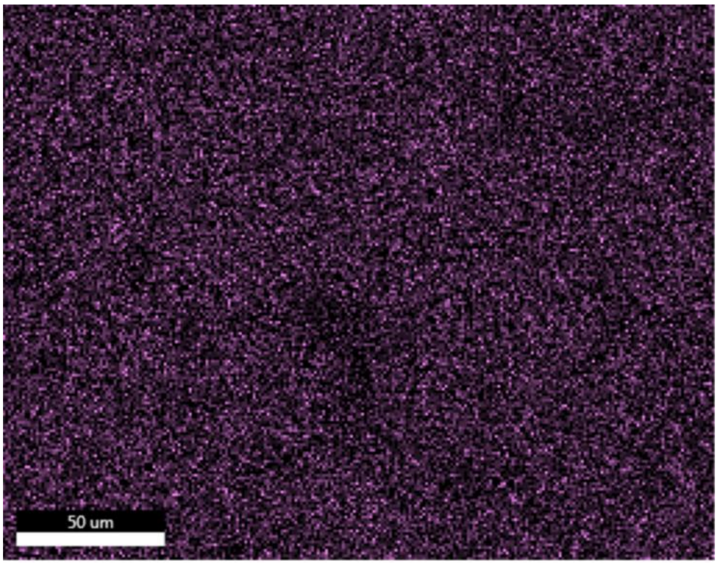

0.25% iBu_7SS-Vi/PP

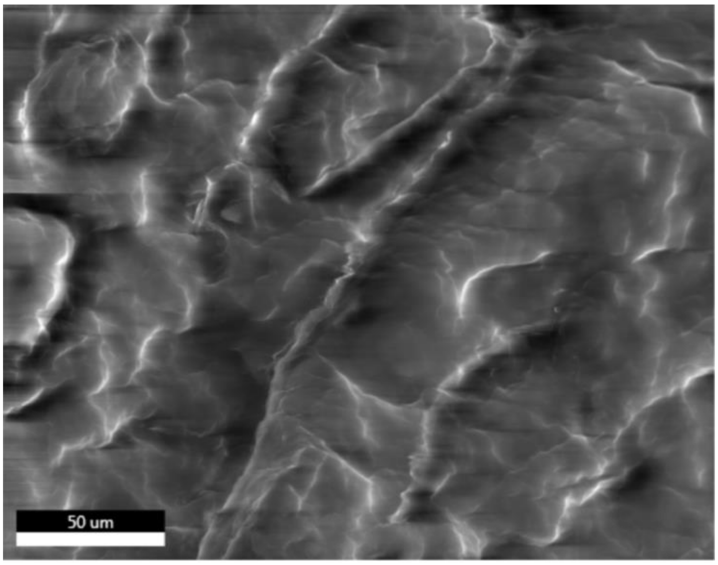

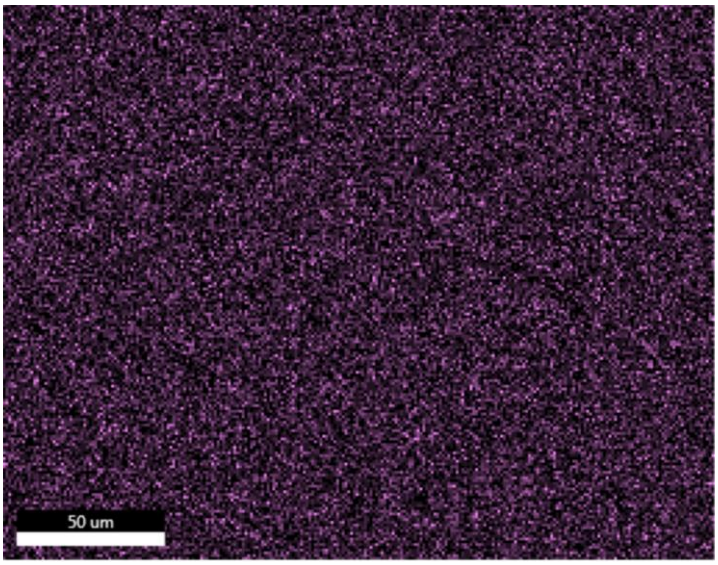

0.5% iBu_7SS-Vi/PP

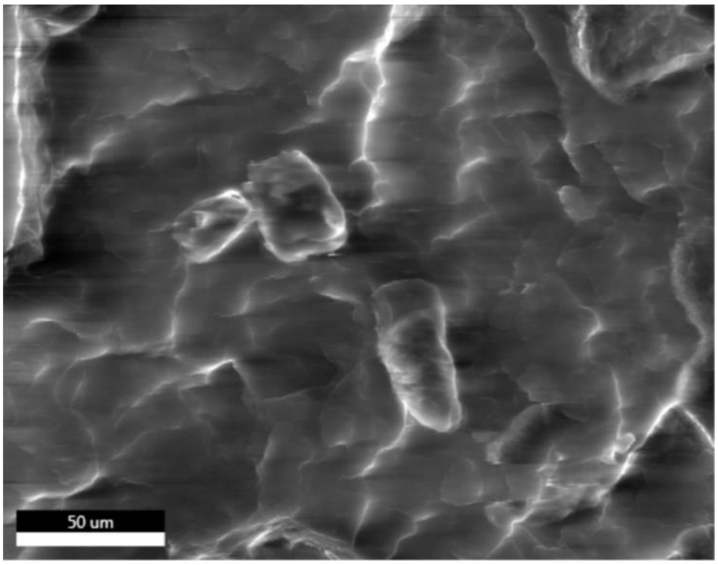

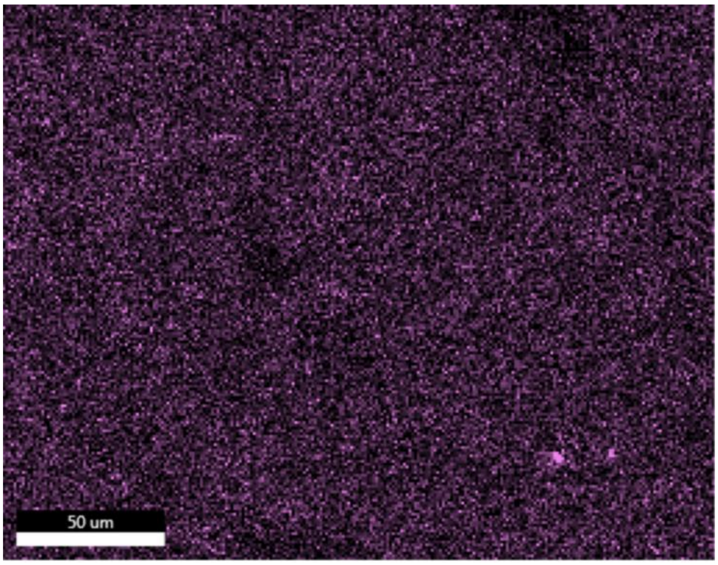

1% iBu_7SS-Vi/PP

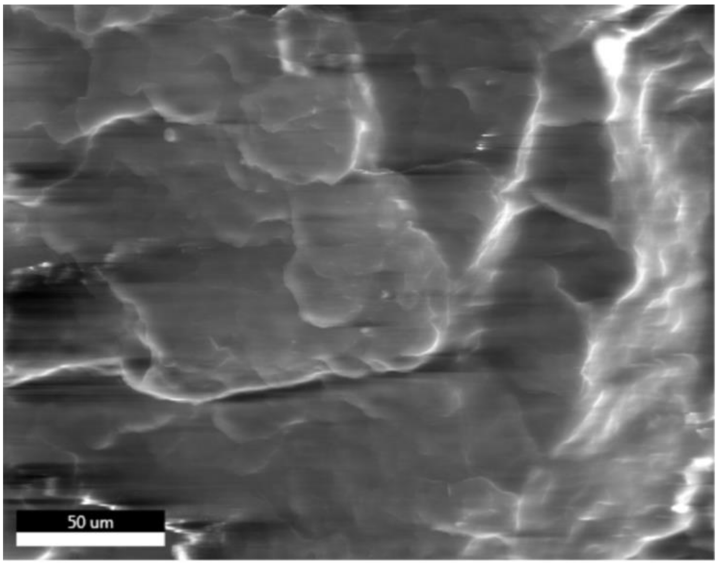

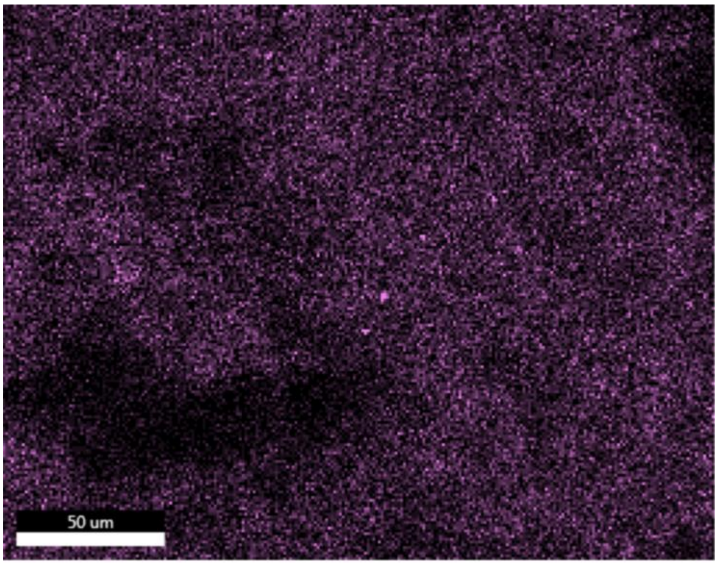

0.1% iBu_7SS-H/PP

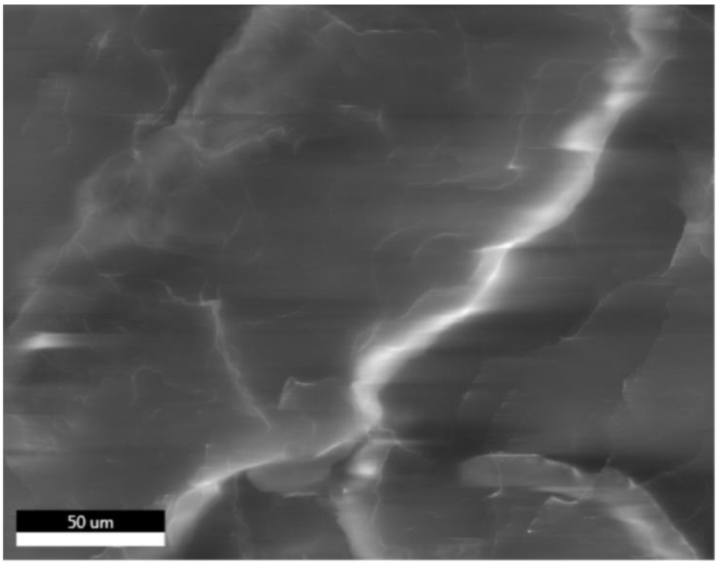

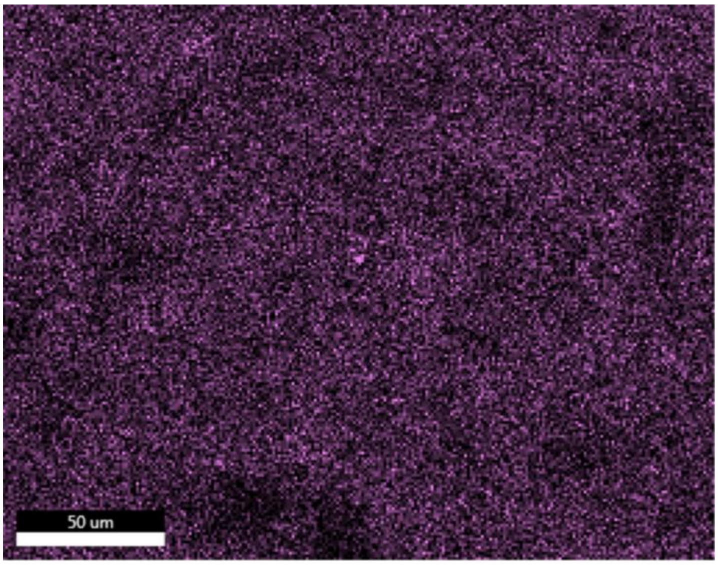

0.25% iBu_7SS-H/PP

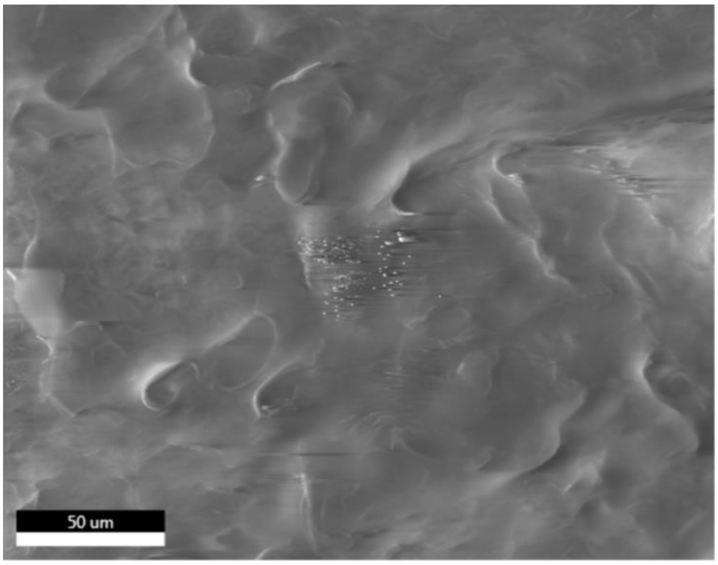

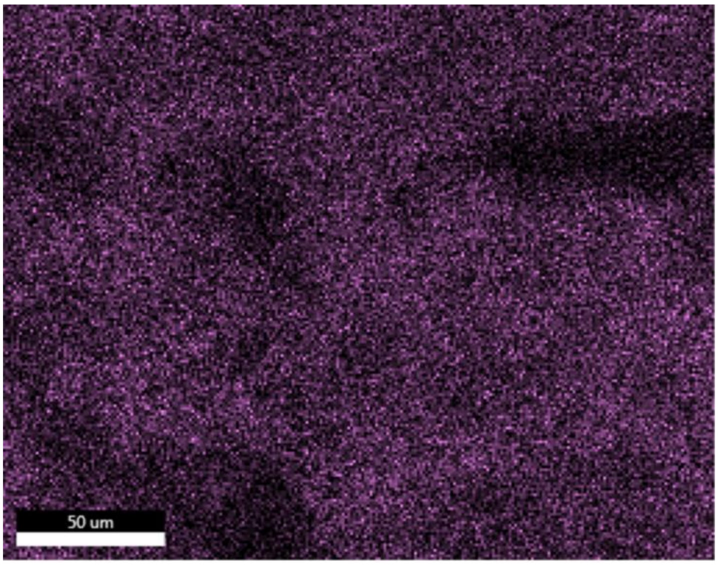

0.5% iBu_7SS-H/PP

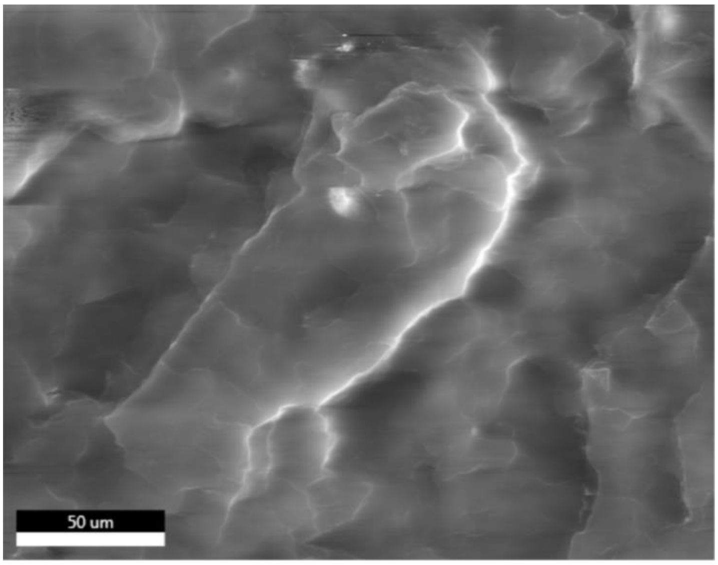

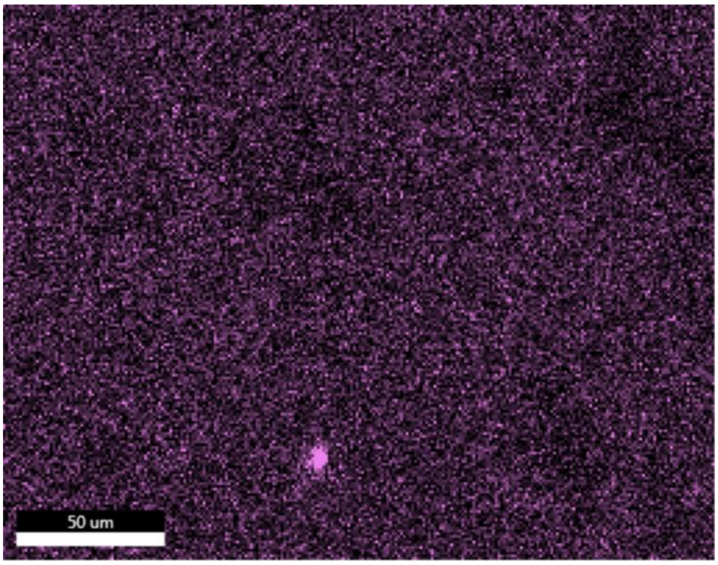

1% iBu_7SS-H/PP

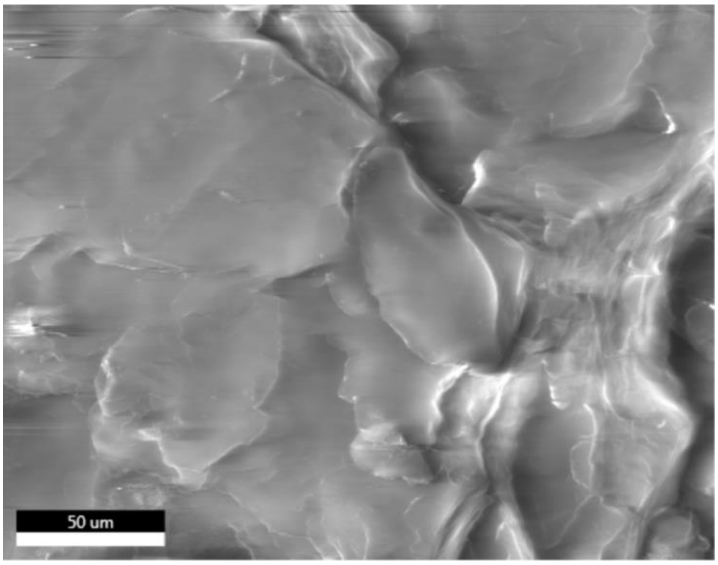

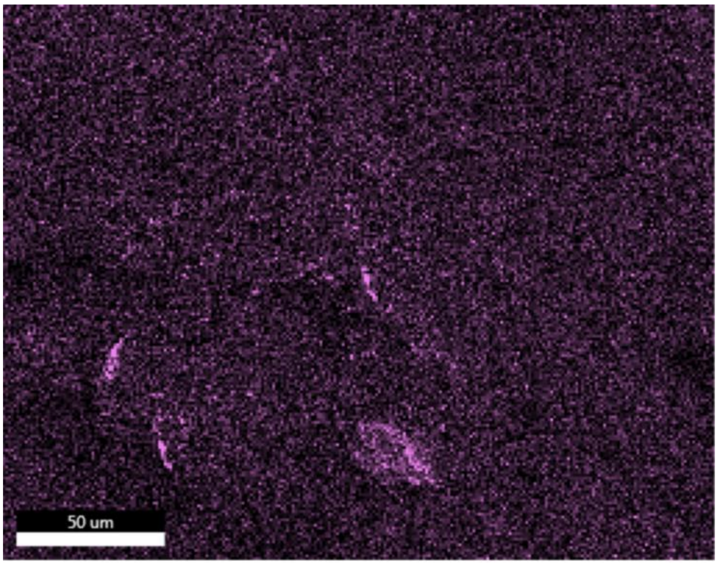

Supplement: Supplementary file 1 [file polymers-13-02124-s001.zip › polymers-1257313-supplementary.pdf]
